# Bioinformatic Analysis of the Perilymph Proteome to Generate a Human Protein Atlas

**DOI:** 10.3389/fcell.2022.847157

**Published:** 2022-04-28

**Authors:** Alina van Dieken, Hinrich Staecker, Heike Schmitt, Jennifer Harre, Andreas Pich, Willi Roßberg, Thomas Lenarz, Martin Durisin, Athanasia Warnecke

**Affiliations:** ^1^ Department of Otolaryngology, Hannover Medical School, Hannover, Germany; ^2^ Department of Otolaryngology, Head and Neck, Surgery, University of Kansas School of Medicine, Kansas City, KS, United States; ^3^ Core Facility Proteomics, Hannover Medical School, Hannover, Germany

**Keywords:** cochlea, perilymph, hearing loss, hereditary inner ear disease, proteome, bioinformatics

## Abstract

The high complexity of the cellular architecture of the human inner ear and the inaccessibility for tissue biopsy hampers cellular and molecular analysis of inner ear disease. Sampling and analysis of perilymph may present an opportunity for improved diagnostics and understanding of human inner ear pathology. Analysis of the perilymph proteome from patients undergoing cochlear implantation was carried out revealing a multitude of proteins and patterns of protein composition that may enable characterisation of patients into subgroups. Based on existing data and databases, single proteins that are not present in the blood circulation were related to cells within the cochlea to allow prediction of which cells contribute to the individual perilymph proteome of the patients. Based on the results, we propose a human atlas of the cochlea. Finally, druggable targets within the perilymph proteome were identified. Understanding and modulating the human perilymph proteome will enable novel avenues to improve diagnosis and treatment of inner ear diseases.

## Introduction

The cochlea is a fluid-filled organ with a complex and highly organised cellular architecture (Glueckert et al., 2018) and consists of functional subunits including the organ of Corti neuroepithelium (hair cells and glial like supporting cells), the spiral ligament and the stria vascularis (ion transport, energy metabolism and immune response) as well as the spiral ganglion (glial cells and neurons that connect the hair cells to the central auditory pathway). These unique regions within the inner ear are susceptible to individual disease processes. As shown in a recent overview of rare diseases that affect hearing, different acquired and inherited diseases result in hearing loss in combination with defects in different organ systems such as integument, renal, heart/vascular, nervous and immune system (Warnecke and Giesemann, 2021). Identifying patterns, which connect the inner ear with other organ systems and the understanding of inner ear diseases on a cellular level might be a promising novel avenue for improved diagnosis and treatment. The different cells of the inner ear are contained in a unique bony otic capsule and are surrounded by perilymph which is a liquid similar to cerebrospinal fluid (CSF). In addition to understanding the genetic disorders that affect the inner ear, we also need to consider numerous proteins that are involved in the physiology of hearing, and how they could be linked with the pathophysiology of different types of hearing impairment (Alawieh et al., 2015196; Lysaght et al., 2011; Thalmann, 2001270).

The main reason for the limited understanding of the diverse causes of hearing loss is the poor accessibility of the inner ear. Since tissue biopsies from the inner ear for in depth molecular analysis are not possible without causing significant structural permanent damage, the accessibility of the cochlea during surgical procedures offers the unique opportunity to gain a “fluid biopsy” by perilymph sampling. Perilymph is one of the two types of inner ear fluids and shows a similar electrolyte composition as the blood serum but differs in its protein contents. Due to the smallness of the organ, the human cochlea contains only a very limited amount (in a microliter range) of perilymph. Thus, only a tiny volume of perilymph samples can be obtained. Improving technologies like mass spectrometry-based protein analysis enable the detection and analysis of hundreds of proteins in only a few microliters of body fluids (Walther and Mann, 2010; Gaspari and Cuda, 2011; Ong et al., 2015) making investigations of the human inner ear proteome possible. Indeed, others and we provided solid proof of the feasibility of perilymph sampling and analysis to define the cochlear microenvironment (Edvardsson Rasmussen et al., 2018; Lin et al., 2019a; Trinh et al., 2019; Wichova et al., 2019) not only by analysing the proteome (Schmitt et al., 20171923), but also the inflammasome (Warnecke et al., 2019), miRNA profile (Shew et al., 2018) and proteins related to the heat shock (Schmitt et al., 2018) as well as the BDNF pathway (de Vries Schmitt et al., 2019) in humans by combining the proteomic profile with gene expression arrays, residual organ function and bioinformatic analysis. Based on our previous investigations, we detected 203 proteins in patients who underwent perilymph sampling during cochlear implantation (Schmitt et al., 20171923). We assume that these proteins are derived from the tissue since they were detected in perilymph samples, but not in CSF or blood samples (Schmitt et al., 20171923). The cochlea is a closed fluid-filled system surrounded by a bony capsule preventing the escape of the perilymph fluid to other parts of the body. Therefore, we assume that the origin of the perilymph proteins is very probably the inner ear tissue or the body fluids blood and CSF. The proteins only identified in perilymph samples but not in listed in the blood and CSF databases for this reason were defined as tissue-specific proteins. However, little is known about the function and significance of these proteins. Most importantly, proteome profiles could not only provide insight into the molecular fingerprint of the human inner ear (Alawieh et al., 2015196) but also aid in developing therapies targeted to individual inner ear cells. Progress in the field of artificial intelligence and the deployment of machine learning in scientific research offer new approaches to characterise proteins (Bausch-Fluck et al., 2018) and to set a framework for a molecular fingerprint specifically of complex organs like the inner ear (Jackler and Jan, 2019). Bioinformatic analysis in combination with systematic literature review can be a valuable tool to identify genes and protein targets for hearing disorders such as sudden sensorineural hearing loss (Nelson et al., 2021). Based on data collected from human and animal studies, the National Institute of Health established the “Illuminating the Druggable Genome” programme to enable a better understanding of the function of poorly studied genes especially of receptors, ion channels and kinases (Nguyen et al., 2017; Berginski et al., 2020). Especially when considering the lack of inner ear specific drugs, the proteomic approach might be of significance in order to identify druggable targets among the perilymph proteins.

The aim of the present study was to provide a comprehensive landscape of human inner ear proteins. Using proteomic and bioinformatic analysis, we sought to elucidate which cells might be responsible for the presence of individual proteins in the perilymph and whether these proteins can function as druggable targets. This way, a first approach to identify targets for inner ear specific therapies may be found. Since the bulk of small molecules that interact with proteins are inhibitors, we also have the opportunity to use this data to design *in vitro* and *in vivo* studies of inhibiting particular proteins in normal inner ear tissue allowing us to dissect the effect of individual or groups of proteins on hearing rather than correlating the effects of hundreds of proteins at a time. This approach might be the groundwork for further investigation on the molecular signature of the inner ear, providing an overview of specific proteins secreted from inner ear tissue, and hopefully new opportunities for the treatment of hearing impairment in the future.

## Materials and Methods

Sampling and proteome analysis of the specimen has been performed previously and the results have been made available in a prior publication (Schmitt et al., 20171923). The methods for sampling and proteome analysis have been also published accordingly (Schmitt et al., 20171923) and are summarised below. In the present study, the proteome profile resulting from the previous study was subjected to further in depth bioinformatic analysis.

### Sampling

Sampling of perilymph was performed as described previously (Schmitt et al., 20171923). Briefly summarised, a total of 41 perilymph samples were collected from 38 patients. Of these, 34 patients underwent cochlear implantation (CI) and four patients suffered from vestibular schwannoma (VS). Three patients received bilateral CI. The cochlear implantations during which perilymph sampling was performed were exclusively performed by the round window approach. The bony overhang when present was drilled to enable access to the round window. Prior to perilymph sampling, the surgical area was washed and all fluids were suctioned carefully to avoid contamination. The round window membrane was punctured manually under the microscope using a modified micro glass capillary with a bevelled sharp tip. Thereby, the tip of the micro glass capillary was inserted into the perilymphatic space and perilymph was taken in by capillary forces (Schmitt et al., 20171923). For VS removal, a translabyrinthine approach was chosen via the semicircular canal. The perilymph samples were macroscopically controlled for possible blood contamination and additionally by centrifugation of the sample. In case of a minor red pellet (red blood cells) only the supernatant was used for analysis and the contamination was documented. In addition to the perilymph sample, also four human cerebrospinal fluid (CSF) samples could be collected via puncture of the dura during VS removal. Three blood samples of both, patients with CI and VS, were collected for comparison of protein content and exclusion of possible contamination of perilymph samples. All samples were immediately cooled on ice and stored at −80°C until analysis by mass-spectrometry. The perilymph sample volumes ranged from 0.5 to 12 µL. Patients aged 8 months up to 80 years were included in this study. The protocols for the collection of perilymph were approved by the Institutional Ethics Committee (approval no. 1883-2013). Written informed consent was obtained from every patient included in this study.

### Proteome Analysis

The perilymph samples were analyzed by an in depth shot gun proteomics approach as outlined in detail in our prior study (Schmitt et al., 20171923). Proteins expressed in perilymph samples were detected by nanoscale liquid chromatography coupled to tandem mass spectrometry (nano-LC-MS), allowing the analysis of many proteins simultaneously. Raw data was processed using Max Quant Software and human entries of the Swissprot/Uniprot data base (RRID:SCR_002380), identifying names and Uniprot IDs of the expressed proteins, their relative quantification values by label free quantification (LFQ), as well as their corresponding genes.

### Bioinformatical Analysis

**FIGURE 1:**
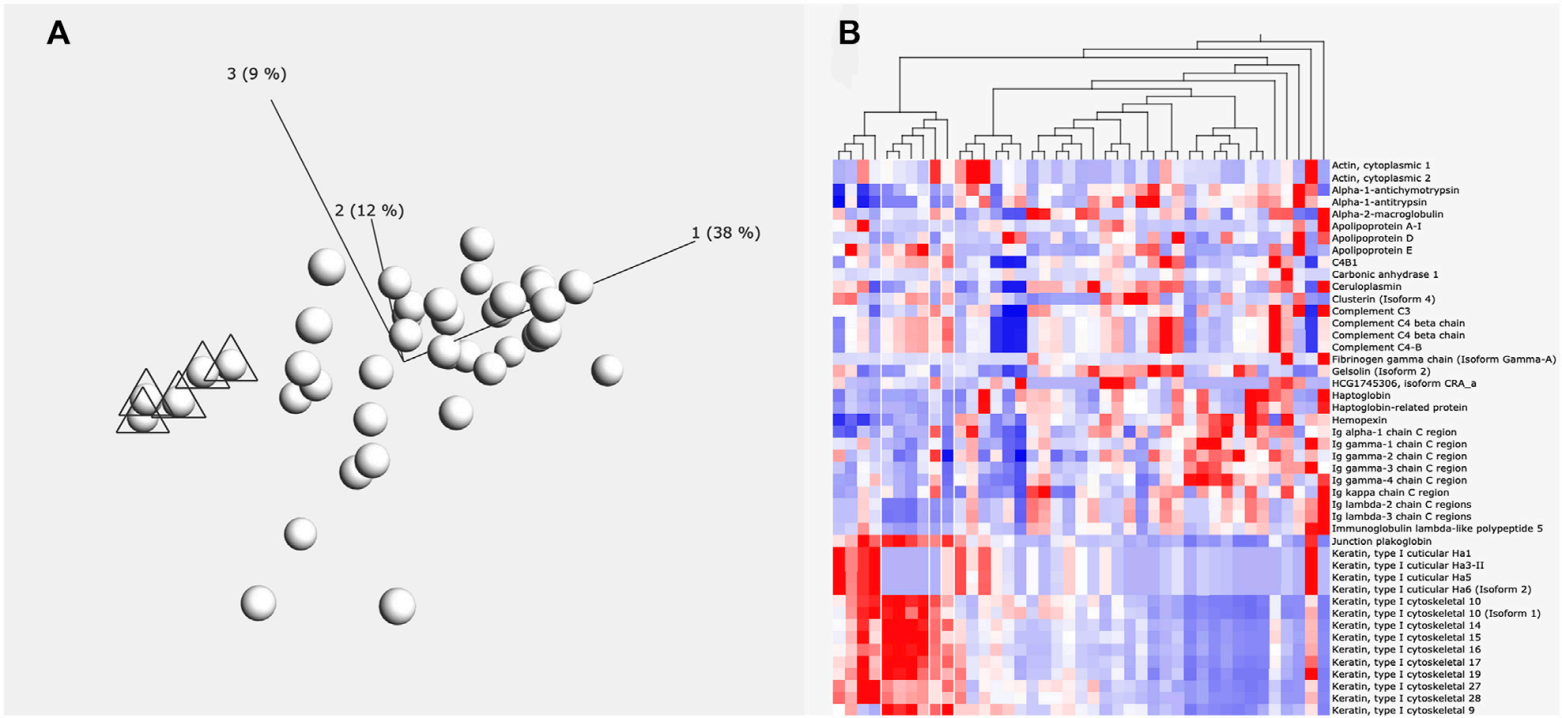
**(A)** Principle component analysis of proteins. Six individuals marked with triangles are grouped by the levels of protein expression (principle components) using Qlucore Omics Explorer. **(B)** Heatmap. This heatmap shows how the protein expression pattern of the six individuals identified in A differs from the other patients. White lines indicate the six grouped individuals. Red colour indication increased expression and blue colour reduced expression levels.

For initial cluster analysis, LFQ data for each identified protein was uploaded into Qlucore Omics Explorer (Malmo, Sweden). A principal component analysis (PCA) was carried out to allow visualization of each patient’s total perilymph proteome (Figure 1A). Variance filtering using the standard deviation (S/Smax) was carried out to identify a subset of proteins that maximised the PCA projection allowing assessment of relatedness of the individual samples. These data were then converted to a heat map (Figure 1B). Individual groups of patient data could then be selected to identify the protein data that characterises that particular selected group.

The protein content of the perilymph samples was compared with proteins detected in CSF and blood samples to identify tissue-specific proteins, i.e., proteins that were detected in the perilymph but not in CSF or blood samples as described previously (Schmitt et al., 20171923). To rule out possible bias due to the small sample size of CSF and blood, the proteins identified as tissue-specific were compared with the proteome database of blood (http://www.plasmaproteomedatabase.org/) and CSF (Zhang et al., 2015107) published by different groups. These tissue-specific proteins and their characteristics (Uniprot ID, protein and gene name) were used for further bioinformatic in depth analysis.

The characterisation of individual proteins was carried out using open access platforms based on artificial intelligence like Pharos (https://pharos.nih.gov; RRID:SCR_016924). The Pharos interface offers data from the “Illuminating the Druggable Genome” programme, which was initiated in 2014 by the National Institutes of Health (NIH) Common Fund. The Pharos website provides information not only on the druggability of proteins, i.e., whether the protein can be targeted by an approved drug or another known active ligand, but also on protein function, expression sites, associated diseases, protein structure, interactions and more. Pharos was created to enable “serendipitous browsing,” which served as a valuable tool to gather information on proteins which were initially only described by their name and UniProt ID (Nguyen et al., 2017). It was therefore our primary source to characterise proteins including their function, possible associated diseases, and their expression sites. In addition, a short abstract about each protein’s characteristics and significance was provided together with a solid summary on each protein’s related publications and information on druggability and related targets.

Next, we enhanced the data found on Pharos with information found on the UniProt website (https://www.uniprot.org; RRID:SCR_002380). This website provides specific information on the biochemical function of each protein. It also allows the further differentiation into specific isoforms (The UniProt Consortium, 2017). It was therefore specifically useful, whenever the UniProt ID identified by LC-MS did not have a corresponding entry on the Pharos website. Some proteins identified are associated to a series of rare, genetic diseases. The OMIM website, i.e., Online Mendelian Inheritance in Man (OMIM): A knowledgebase of human genes and genetic phenotypes (https://www.omim.org; RRID:SCR_006437), provides solid information on the corresponding gene to each of the identified proteins (Amberger and Hamosh, 2017). We were therefore able to enrich our information basis with information on corresponding genes and genetic disorders, where the identified proteins may have a key role in pathophysiology. A profound literature research was performed to relate our detected human perilymph proteome with the body of knowledge about the cochlear research already gathered in animal models, cell culture investigations and post-mortem samples. For this purpose, each individual protein was researched with respect to a cochlear or inner ear relating context.

The proteins were classified according to their druggability status as identified on the Pharos website, different functional groups reflecting their biochemical functions and whether information about the protein in an inner ear context was available. For certain protein clusters, we performed an ingenuity pathway analysis (IPA; RRID:SCR_008653) to identify and visualise patterns of protein-protein and protein-drug interactions, pathways, diseases and disorders, in which the proteins are predominantly involved. Using this structured information, we derived a map of the human cochlea depicting suggested protein expression patterns, i.e., which cell could be the origin of the protein identified in human perilymph.

## Results

A total of 878 proteins were identified overall in the 41 perilymph samples. In average, 328 proteins were detected per perilymph sample showing clearly a variable protein composition between individuals. Analysis by PCA plot and clustering demonstrates that multiple different patient groups can be identified based on their profile in perilymph proteins (Figures 1A,B). There was no correlation to age or gender. Evaluating the identified proteins, keratin subtypes, immunoglobulins and complement factors dominate the resulting clusters with some patients expressing large amounts and others expressing only low levels of these proteins in perilymph. These of course are not specific to inner ear function. To focus more specifically on inner ear function, we compared our data to identify peptides or proteins that are expressed into perilymph but are not detected in our samples of CSF or serum.

For the remaining 203 perilymph specific proteins, we performed a bioinformatic analysis. Through Pharos, Uniprot and OMIM we acquired information about localization, function, expression, associated diseases and druggability of selected proteins. Where possible, proteins were assigned to the 23 different functional groups as proposed by Nishio et al. (2015) in a previous work evaluating gene expression in the cochlea. For each protein, these data were then matched with information regarding the cochlea as found in the literature. The results are summarised in Supplementary Table S1. This table contains all 203 proteins, including the 138 proteins for which information regarding the cochlea was available from animal studies of the cochlea, from cell culture experiments or from analysis of post-mortem samples. If known, a possible cell of origin in the cochlea was assigned assuming that this may be the source for the secreted protein that was detected in the perilymph. Many of the perilymph specific proteins were present in only a few patients -meaning proteins were identified in 1–12 perilymph samples - making cluster analysis less effective.

**FIGURE 2:**
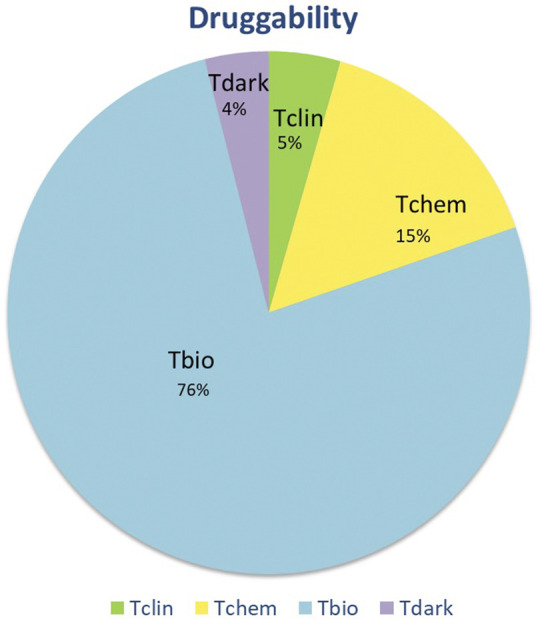
Druggability status of the 203 tissue-specific proteins. Based on Pharos, targets are divided into four categories. Of all tissue specific proteins, 5% were identified as targets for clinically approved drugs. For 15% of the proteins, active ligands (Tchem) were identified. Seventy six percent of perilymph proteins have an identifiable gene ontology and can be functionally classified but do not have available inhibitors (Tbio). Finally, a small percentage of identified proteins are unclassified (Tdark).

The proteins were also classified according to their druggability status. Pharos uses four terms to categorise proteins/targets according to their IDG development level: **Tclin:** Targets have at least one active drug. **Tchem:** Targets have at least one active ligand compound with an activity cutoff of <30 nM. **Tbio**: Targets do not have any drug or small molecule activity, do not follow the criteria for Tdark and have known gene ontology terms. **Tdark**: Targets about which virtually nothing is known. They do not have known drug or small molecule activities. Table 1 summarises the proteins with a druggability status “Tclin”, i.e., targets with at least one approved drug. Of the 203 proteins found to be tissue-specific, 9 were classified as Tclin (5%), 31 as Tchem (15%), 155 as Tbio (76%) and 8 were classified as Tdark (4%) as shown in Figure 2. The proteins classified as Tclin, e.g., carbonic anhydrase 3 (CA3), Janus Kinase 1 (JAK1) and others, are summarised in Table 1, including their molecular and cochlear function, their targeting drug and the mode of action of this drug.

**TABLE 1:** Proteins classified as “Tclin” and their targeting drug, including mode of action.

Protein/Gene	Function of protein, especially in the cochlea	Drugs	Mode of action
Carbonic anhydrase 3 (CA3)	Function: Reversible hydration of carbon dioxide	Acetazolamide, Ethoxzolamide, Imatinib, Nilotinib, Lacosamide	All approved drugs are inhibitors
	Cochlea: Facilitating mitochondrial ATP synthesis, detoxifying free radicals, HCO3—secretion into the endolymph		
Thymidine phosphorylase (TYMP)	Function: Reversible phosphorolysis of thymidine, angiogenic factor	Tipiracil	Inhibitor
	Cochlea: Associated with MNGIE-syndrome (Hearing loss in 61%)		
Glucosylceramidase (GBA)	Function: Glycolipid metabolism, hydrolysis of glucosylceramidase	Ambroxol	Metabolite of Bromhexine that stimulates mucociliary action and clears the air passages in the respiratory tract. It is usually administered as the hydrochloride
	Cochlea: Associated with Gaucher disease (case reports of hearing loss)		
Isocitrate dehydrogenase [NADP] cytoplasmic (IDH1)	Function: Catalyses the oxidative decarboxylation of isocitrate to 2-oxoglutarate	Ivosidenib	Inhibitor
	Cochlea: Protects inner ear from oxidative stress during K + Recycling, participates in K + -Transport. Downregulated in age related hearing loss and by lead exposure		
ATP-citrate synthase (ACLY)	Function: Primary enzyme responsible for the synthesis of cytosolic acetyl-CoA in many tissues. In nervous tissue it may be involved in the biosynthesis of acetylcholine	Bempedoic acid	Inhibitor
	Cochlea: Either hair cell specific or strongly upregulated in hair cells compared to non-sensory cells		
Lysosomal alpha-glucosidase (GAA)	Function: Essential for the degradation of glycogen in lysosomes	Miglustat	Inhibitors: Miglustat, Miglitol, Voglibose
	Cochlea: Mutations are associated with Pompe’s disease (associated with hearing loss in 21%)	Miglitol	
		Migalastat	Activator: Migalastat
		Voglibose	
Alcohol dehydrogenase 1B	Function: Oxidoreductase in ethanol metabolism Cochlea: No information available	Fomepizol	Inhibitor
Janus Kinase 1 (JAK1)	Function: Tyrosine kinase that phosphorylates STAT proteins, involved in interferon signal transduction	Sunitinib	All approved drugs are inhibitors
	Cochlea: Regulates proliferation in support cells after hair cell death, STAT3 signalling is important during cochlear hair cell differentiation, downregulated by cisplatin treatment	Nintedanib	
		Tofacitinib	
		Ruxolitinib	
		Fedratinib	
		Baricitinib	
		Upadacitinib	
Aldehyde dehydrogenase, mitochondrial (ALDH2)	Function: Oxidoreductase in the major oxidative pathway of alcohol metabolism	Disulfiram	Inhibitor
	Cochlea: Expressed in surrounding cells		

**FIGURE 3:**
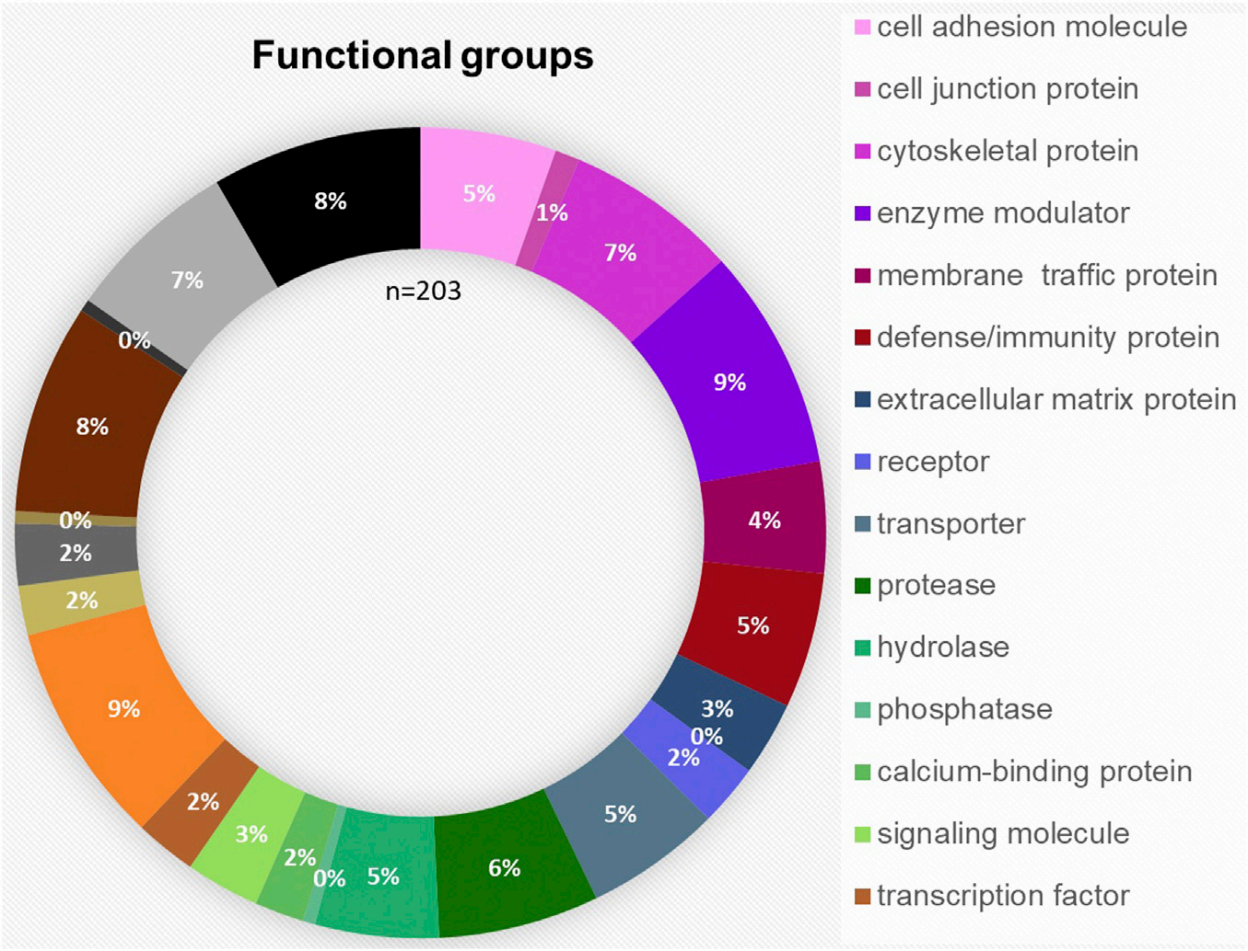
Functional distribution of the 203 proteins classified as tissue-specific after comparison with blood and CSF samples taken from four patients. The most common functional groups among the proteins were “enzyme modulator,” “nucleic acid binding,” “transferase,” “cytoskeletal protein” and “protease.”

The bioinformatic analysis of the 203 proteins allowed a classification in the following 23 different protein families: cell adhesion molecule, cell junction protein, cytoskeletal protein, enzyme modulator, membrane traffic protein, defense/immunity protein, extracellular matrix protein, surfactant, receptor, transporter, protease, hydrolase, phosphatase, calcium-binding protein, signaling molecule, transcription factor, nucleic acid binding, transmembrane receptor, regulatory/adaptor protein kinase, ligase, transferase, lyase and oxidoreductase. The distribution of the functional groups is illustrated in Figure 3. Nucleic acid binding (9%), transferases (8%) and enzyme modulators (9%) were prominently featured. IPA was used to define the potential functional impact of the perilymph proteome. Many of the identified proteins are involved in intracellular signaling pathways and exert specific functions. The most common signaling pathways identified include Glycogen Degradation III, Glycogen Degradation II, EIF2 Signaling, Integrin Signaling and Hepatic Fibrosis Signaling Pathway.

The proteome could further be classified as being involved in Inflammatory Response (*n* = 81), Infectious Diseases (*n* = 50), Cancer (*n* = 188), Organismal Injury and Abnormalities (*n* = 188) and Immunological Disease (*n* = 75). The classification demonstrates the prominence of metabolism processes and injury response.

Some of the tissue specific proteins were found to be involved in pathophysiology of rare, genetic, or syndromic diseases associated with hearing loss, e.g., Collagen type 2 alpha 1 chain (COL2A1) in Stickler syndrome 1 or Myosin heavy chain 9 (MYH9) in various syndromes. These proteins, as well as their function and druggability, are shown in Table 2. Others, such as JAK1 and Cytochrome b-245 heavy chain (CYBB) seem to play a fundamental role in neuronal health. More tissue specific proteins were found to be important for various processes essential for cochlear development and function, among them axogenesis, potassium transport and protection of oxidative stress. These proteins are summarised in Table 3.

**TABLE 2:** Proteins involved in syndromic and hereditary hearing loss.

Gene	Protein	Associated disease	Protein function
GBA	Glucosylceramidase	Gaucher’s disease	Glycolipid metabolism, hydrolysis of glucosylceramidase
		Microtia-atresia	
GAA	α-Glucosidase	Pompe’s disease	Essential for the degradation of glycogen in lysosomes
LPR2	Megalin	Donnai-Barrow syndrome Facio-oculoacoustico renal syndrome (FOAR)	Multi-ligand endocytic receptor, essential for uptake of various ligands (e.g. albumin, apolipoprotein B/E, lipoprotein lipase), involved in cell signalling, essential for normal hearing (Estrogen interaction)
MYH9	Myosin heavy chain 9	DFNA17, Epstein syndrome, Alport sydrome, Sebastian syndrome, Fechtner syndrome, macrothrombocytopenia	Non-muscle myosin, involved in cytokinesis, cell motility and maintenance of cell shape
COL2A1	Collagen type 2 alpha 1 chain	Stickler syndrome I (STL I)	Structural components of the extracellular matrix of chondrocytes and the tectorial membrane
COL11A2	Collagen alpha-2 (XI) chain	Stickler syndrome III, OSMED syndrome, Weissenbacher-Zweymuller syndrome, DFNA13, DFNB53	Structural components of the extracellular matrix of the tectorial membrane, involved in fibrillogenesis
MDH2	Malate dehydrogenase 2	Candidate gene for DFNB39	Malate dehydrogenase catalyses the reversible oxidation of malate to oxaloacetate in citrate cycle
LAMB1	Laminin subunit beta	Alport syndrome, candidate gene for DFNB14	Implicated in a wide variety of biological processes including cell adhesion, differentiation, migration, signalling, neurite outgrowth and metastasis
CDH11	Cadherin 11	Elsahy Waters syndrome	Calcium-dependent cell adhesion protein, highly expressed during cochlear development
CDC42	Cell division control protein 42 homolog	Takenouchi-kosaki sydrome	Cdc42 influenced the maintenance of stable actin structures through elaborate tuning of actin turnover, and maintained function and viability of cochlear hair cell, part of hair cell polarity establishment and required for stereociliogenensis
TYMP	Thymidine phoshorylase	Mitochondrial neurogastrointestinal encephalomyopathy (MNGIE syndrome)	Reversible phosphorolysis of thymidine, angiogenic factor

**TABLE 3:** Other proteins.

Gene	Protein	Function	Function in cochlea	Druggability
Neuronal health and protection from oxidative stress				
JAK1	Janus Kinase 1	Tyrosine kinase that phosphorylates STAT proteins, involved in interferon signal transduction	Regulates proliferation in support cells after hair cell death, STAT3 signalling is important during cochlear hair cell differentiation, downregulated by cisplatin treatment	Tclin
TGFBR2	Transforming growth factor receptor 2	Transmembrane serine/threonine kinase forming withTGFBR1, the non-promiscuous receptor for the TGF-beta cytokines TGFB1, TGFB2 and TGFB3	Associated with scarless wound healing, downregulated after electrode insertion (CI-Insertion), and after noise exposure	Tchem
CYBB/NOX2	Cytochromeb-245 heavy chain	Critical component of the membrane-bound oxidase of phagocytes that generates superoxide	Ginkgolide B (reduces NOX2 expression) decreased ROS generation and therefore reduction of cisplatin induced ototoxicity	Tchem
TYMP	Thymidine phosphorylase	Reversible phosphorolysis of thymidine, angiogenic factor		Tclin
HMGB1	High-mobility group box 1	one of the major chromatin-associated non-histone proteins and acts as a DNA chaperone; involved in immune response, extracellular component: acts as chemokine	May play an important role in cochlea development, possibly influences SGNs’ survival following ototoxic exposure; in stressful conditions liberated from Deiters’ cells to regulate the epithelial reorganization of injured organ of Corti	
TIMP1	Tissue inhibitor of metallo-proteinases	Metalloproteinase inhibitor, also functions as a growth factor that regulates cell differentiation, migration and cell death and activates cellular signalling cascade	early downregulated and subsequent upregulated during sensory cell degeneration. Inhibits MMP, which might participate in cochlear response to acoustic overstimulation and can serve as a novel therapeutic target	Tbio
Axonogenesis				
PTPRS	Protein tyrosine phosphatase receptor type S	Cell surface receptor that binds to glycosaminoglycans, required for normal brain development	involved in primary axonogenesis, and axon guidance during embryogenesis, also implicated in the molecular control of adult nerve repair	Tchem
NAMPT	Nicotinamide phosphoribosyl-transferase	Catalyses the condensation of nicotinamide with 5-phosphoribosyl-1-pyrophosphate to yield nicotinamide mononucleotide, an intermediate in the biosynthesis of NAD.	Neuroprotective Nampt Inhibitor P7C3 Demonstrates Otoprotection in an Age Related Hearing Loss Model	Tchem
UCHL1	Ubiquitin carboxyl-terminal hydrolase isozyme	involved both in the processing of ubiquitin precursors and of ubiquitinated proteins. This enzyme is a thiol protease that recognises and hydrolyses a peptide bond at the C-terminal glycine of ubiquitin	Downregulated after Gentamycin exposure, Deficiency accelerates Gentamycin-induced ototoxicity	Tchem
Otoprotection and cell adhesion molecules				
GLUL	Glutamine synthetase	Glutamine synthetase that catalyses the ATP-dependent conversion of glutamate and ammonia to glutamine	May function to limit the perilymphatic glutamate concentrations, the most important afferent neurotransmitter in the cochlea	Tchem
CEACAM16	Carcino-embryonic antigen-related cell adhesion molecule 16	secreted glycoprotein, adhesion protein	Interacts with TECTA. May have a role in connecting stereocilia with the tectorial membrane. Required for proper hearing over an extended frequency range, it may play a role in maintaining the integrity of the tectorial membrane	Tbio
CKM	Creatine kinase M-type	Reversibly catalyses the transfer of phosphate between ATP and various phosphogens	Considered to supply ATP for the Na,K-ATPase that mediates the high KCl of endolymph	Tbio
Potassium transport and regulation				
CA3	Carbonic anhydrase	Reversible hydration of carbon dioxide	Facilitates mitochondrial ATP synthesis and detoxifying free radicals resulting from ATP synthesis, mediates HCO3− secretion into the endolymph, effects the endocochlear potential	Tclin
IDH1	Isocitrate dehydrogenase 1	Catalyses the oxidative decarboxylation of isocitrate to 2-oxoglutarate	Participates in K+ transport, protects inner ear from oxidative stress during k*recycling, downregulated in age related hearing loss and lead exposure	Tclin

**FIGURE 4:**
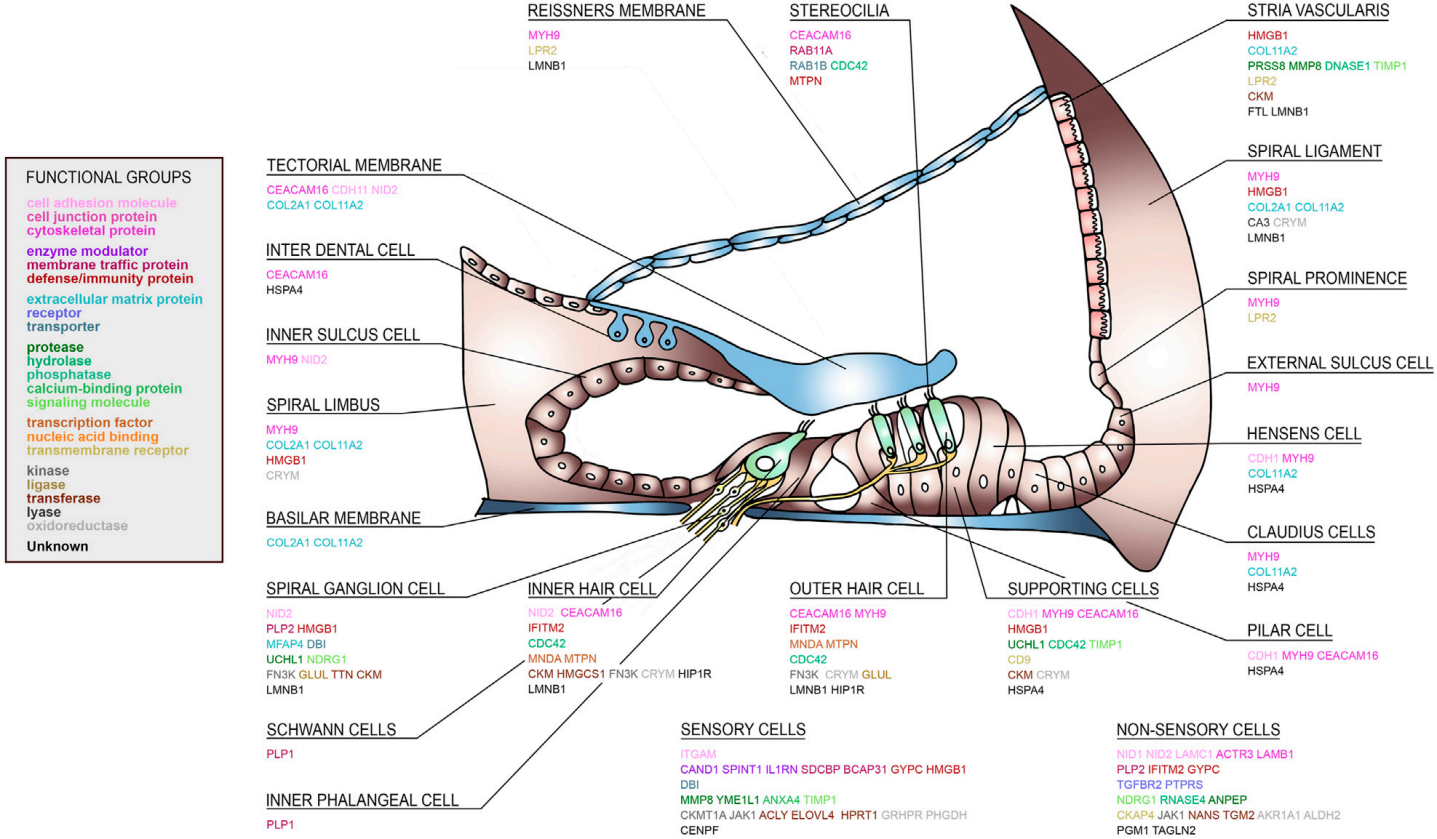
A human cochlear atlas of proteins. Schematic drawing of the human organ of Corti, lateral wall and spiral ganglion showing the complex cellular architecture of the cochlea. Individual proteins were classified into functional groups and assigned to their putative cell source.

Since perilymph samples could potentially be contaminated with blood or CSF, the identified perilymph proteins were then cross referenced to the proteome database of blood (http://www.plasmaproteomedatabase.org/) and CSF (Zhang et al., 2015107). Thus, the number of perilymph-specific proteins was further reduced to 27. A literature search was then carried out and potential source cells for selected proteins identified (Figure 4). The sensory cells, i.e., inner hair cells (IHC) and outer hair cells (OHC), supporting cells and spiral ganglion cells (SGC) express many of the identified perilymph proteins although all functional regions of the inner ear may potentially contribute to the profile of perilymph proteins.

## Discussion

To our knowledge, this is the first study providing a human cochlear atlas of protein expression based on proteome data derived from perilymph analysis. Thus, despite rapid molecular and bioinformatic analysis approaches, only limited information on the molecular changes related to hearing loss is available. This limited information is mainly derived from cochlear cell cultures, animal models and post-mortem investigations and studies using cochlear tissue for research [(Liu et al., 2019), (Edvardsson Rasmussen et al., 2018)]. For example, from investigations on mice supporting cells, main molecular pathways, such as “focal adhesion,” “PI3K-Akt signalling” and “ECM-receptor interaction” were found that could give crucial information on essential processes for supporting cells (Requena et al., 2018).

Identifying tissue specific proteins in fluid biopsies of the human perilymph and correlating proteins and peptides to individual cells within the cochlea might allow to unravel the molecular composition of the human inner ear. This will lead to a better understanding of the underlying pathophysiology of hearing impairment in humans. In the future, new methods to provide tissue samples of the human inner ear could contribute to our understanding of hearing and our knowledge about cochlear protein expression. Of the proteins identified as tissue-specific, approximately 33% had never been reported to be associated with the inner ear in animal studies or from *in vitro* experiments despite a thorough literature investigation. The remaining proteins were related to inner ear physiology and pathophysiology. In the following sections, individual perilymph proteins were grouped according to the information that was gathered from the current state of listed databases.

### Hereditary and Syndromic Hearing Loss

Interestingly, several proteins were found in the human perilymph of individual patients that were coded by genes involved in hereditary and also in syndromic diseases associated with hearing loss as listed in Table 2. Knowledge on the expression pattern of proteins in the human cochlea in of importance not only to exactly understand the molecular mechanisms of hearings loss as shown recently for GJB2 (Del Castillo and Del Castillo, 2017), but also for the development of targeted therapeutics. For example, glucosylceramidase or glucocerebrosidase is an enzyme located in the lysosomes where it degrades the glycosphingolipid glucosylceramide. Deficiencies in this enzyme cause a rare lysosomal storage disease called Gauchers disease, which is characterised by hepatosplenomegaly, anemia, thrombocytopenia, skeletal disease and neuropathology including sensorineural hearing loss. Also, recent evidence suggests a link between mutations in the gene coding for glucocerebrosidase and Parkinson’s disease (Sidransky et al., 2009). Alternatively, ceramide can serve as backbone in the building of glycosphingolipids, the first step being catalyzed by glucosylceramidesynthase (GCS), which produces glucosylceramide, the simplest member of this family (Messner and Cabot, 2010). The protein α-glucosidase (GAA) is essential for the degradation of glycogen in lysosomes. Defects in its gene are associated with Pompe’s disease, another lysosomal storage disorder. Especially infantile Pompe patients suffer from hearing loss (van der Beek et al., 2012a). From animal models, storage of glycogen in the organ of Corti has been assumed as the main cause for hearing impairment associated with GAA gene defects (van der Beek et al., 2012b).

Another tissue specific protein is megalin (LPR2). Mutations in the LPR2 gene can cause facio-oculoacustico-renal (FOAR) and Donnai-Barrow syndrome. It could provide partial otoprotection for hair cells since the endocytosis of aminoglycosides is megalin (LPR2) dependent (Steyger et al., 2018). Also, it might play an important role as a mediator of estrogen dependent effects in the inner ear and must be considered in early presbycusis in estrogen-deficient patients (König, 2008). The receptor is expressed in the stria vascularis in the inner ear (Mizuta et al., 1999; Tauris et al., 2009) and may act as a drug receptor.

Myosin heavy chain 9 (MYH9) is expressed in various cells of the organ of Corti (Mhatre et al., 2006818; Lalwani et al., 2000; Mhatre et al., 2004) and may be involved in reorganization of the actomyosin network (Nishio et al., 2015). Mutations in MYH9 cause DFNA17 and can also result in a series of syndromes associated with hearing loss in up to 50% (e.g., Alport syndrome, Epstein syndrome, Fechtner syndrome and macrothrombocytopenia with progressive sensorineural deafness).

Of the collagens known to be expressed in inner ear tissue, collagen type 2 alpha 1 chain is a structural component of the tectorial membrane, spiral limbus, and other connective tissue structures in the inner ear (Khetarpal et al., 1994; Goodyear and Richardson, 2002; Nishio et al., 2015) as found in the perilymph of some of our patients. Mutations in COL2A1 are associated with Stickler syndrome I, STL I, which presents with hearing impairment in about half of the affected patients (Acke et al., 2012). In addition, collagen type 11 alpha two chain was detected. It is a structural component of the tectorial and basilar membrane, (Shpargel et al., 2004), and can be detected in Hensen’s and Claudius’ cells, in the spiral ligament, the stria vascularis and the spiral limbus (Nishio et al., 2015). Mutations in the COL11A2 gene are associated with type III Stickler syndrome, otospondylomegaepiphyseal dysplasia (OSMED syndrome), Weissenbacher-Zweymuller syndrome, autosomal dominant non-syndromic sensorineural type 13 deafness (DFNA13) (McGuirt et al., 1999), and DFNB 53 deafness (DFNB53).

Another protein detected in the perilymph is malate dehydrogenase 2. The coding gene MDH2 is expressed in the cochlea, but is more abundant in the utricle (Spinelli et al., 2012) and might be a candidate gene for the autosomal recessive hearing disorder DFNB39 (Sajan et al., 2007), in which still no causative gene is identified.

Laminin subunit beta (LAMB1) may be a candidate gene (differentially expressed by > 1,5-fold) in patients suffering from DFNB14, which accounts for non-syndromic human autosomal recessive deafness, for which no causative gene has been identified to date (Sajan et al., 2007). Also, it shows different expression in Alport syndrome, which could be compensatory and/or pathogenic (Funk et al., 2018). Cadherin 11 was also found in the human perilymph. Mutations in CDH11 are associated with Elsahy Waters syndrome and affected individuals show an inter- and intrafamilial variability regarding the presence of mixed hearing loss. Interestingly, CDH11 knockout mice develop only conductive hearing loss, indicating that CDH11 might be involved in middle ear cavitation (Kiyama et al., 2018).

Finally, cell division cycle 42 (CDC42) is a protein coding gene. The resulting protein is a small GTPase and is involved in cell cycle regulation. The protein is expressed in OHC and IHCs and influences the maintenance of stable actin structures through elaborate tuning of actin turnover, and maintained function and viability of stereocilia and cochlear hair cells (Ueyama et al., 2014). Mutations in the CDC42 gene are associated with Takenouchi-Kosaki syndrome, in which some patients develop sensorineural hearing loss.

### Neuronal Health and Cochlear Development

Some of the identified proteins are not known to be involved in genetic hearing loss, but are integral part of pathways that affect neuronal health and protection from oxidative stress and are summarised in Table 3, (subheading: “Neuronal health and protection from oxidative stress”). For example, JAK1 is a component of the STAT3 signalling pathway, which plays an important role during mouse cochlear hair cell differentiation (Chen et al., 2017). The JAK1/STAT3 pathway may be involved in hair cell regeneration via cytokine stimulation of the cell cycle to mediate supporting cell proliferation within the first hours after hair cell death. In addition, activation of the JAK1/STAT3 pathway, e.g., by erythropoietin, can enhance the protective effects of BDNF (Berkingali et al., 2008).

The transforming growth factor receptor 2 (TGFBR2) as well as its ligand TGFB3 are known to be associated with scarless wound healing. Both proteins are downregulated in experimental models of cochlear electrode insertion trauma, revealing a therapeutic target for fibrotic scar prevention in cochlear implantation (Bas et al., 2015). They are also downregulated in cochlear samples after noise exposure (Murillo-Cuesta et al., 2015). Another pathway involved in cochlear health is the Akt-Nrf2-HO-1 pathway.

Cytochrome b-245 heavy chain (CYBB) is coded by the NOX2 gene and is involved in the generation of reactive oxygen species such as superoxide. A targeting ligand is Ginkgolide B, an extract from gingko leaves, which reduces the generation of reactive oxygen species by decreasing NOX2 expression and by activating the Akt-Nrf2-HO-1 pathway, which, in turn, leads to inhibition of mitochondrial apoptosis and attenuation of cisplatin-induced ototoxicity *in vitro* and *in vivo* (Ma et al., 2015). Thus, modulation of CYBB may present a potent mechanism for protecting the inner ear from oxidative stress.

Another potent protector for neurons and glial cells could be thymidine phosphorylase (TYMP) since it has been shown to be increased after ischemic injury (Naples et al., 2020). Several other positive effects of TYMP have been reported such as proangiogenic, anti-apoptotic and pro-thrombotic (Li and Yue, 2018). Some inflammatory cytokines induce TYMP expression, and it has been reported that TYMP promotes the breakdown of the blood-brain barrier (Li and Yue, 2018). It is unclear how TYMP can be released in the perilymph. Since TYMP is expressed intracellular in vascular endothelial cells, its presence in the perilymph could be due to the disruption of the blood-labyrinth-barrier and due to damage to the endothelial cells of the cochlear blood vessels. Another source of TYMP are immune cells in the circulation. Loss-of-function mutations in TYMP cause mitochondrial neurogastrointestinal encephalomyopathy (MNGIE syndrome) (Li et al., 20111016; Nishino et al., 1999), which is associated with hearing loss in 61% of the cases (Li et al., 20111016). Mitochondrial neurogastrointestinal encephalomyopathy and other rare genetic disorders are often associated with hearing loss demonstrating that there are multiple functional subregions of the cochlea that have similarities to very different tissue types (Hiraki et al., 2010). Until now, treatment of patients suffering from MNGIE is based on supportive care and symptom control. There have been some approaches with hemo- or peritoneal dialysis to reduce the high thymidine levels resulting from TYMP deficiency, but their auditive function did not improve (Yadak et al., 2017). Also, approaches with enzyme replacement therapies are currently in development and approved for clinical trials in the United Kingdom. The only approved drug targeting TYMP found in our study is tipiracil, a potent inhibitor of TYMP that is used in the treatment of colorectal carcinoma.

Moreover, factors involved in cochlear development were found in the human perilymph such as the high-mobility group box 1 (HMGB1). HMGB1 is found in the cochlear nucleus throughout postnatal development and is widely distributed in multiple types of cells, including sensory hair cells and supporting cells in the organ of Corti, the stria vascularis, spiral ligament, microvessels within the cochlear lateral wall, spiral limbus, spiral ganglion neurons and glial cells. Through its extra- and intracellular functions, it can influence SGNs after ototoxic exposure (Ladrech et al., 2017). It is also released from Deiter’s cells under stressful conditions to regulate epithelial reorganization through engagement of RAGE (Ladrech et al., 2013). In a murine model of noise-induced hearing loss, inhibition of HMGB1 expression in the cochlea attenuates oxidative stress and inflammation (Shih et al., 2021). Another protein that might be involved in inflammation is the tissue inhibitor of metalloproteinases TIMP1. TIMP1 mediates cochlear response to acoustic overstimulation by regulating the concentration of matrix metalloproteinases (MMP). Some forms of acquired hearing loss are associated with MMP expression. For example, higher concentrations of MMP, especially MMP2 and MMP9, can favour noise induced hearing loss. TIMP1 regulates the concentration of MMP and may therefore serve as a novel therapeutic target. On the other hand, TIMP1 may - in high concentrations or under certain conditions - also cause hair cell death in the cochlea. Therefore, further investigations are needed to understand the optimum concentrations of TIMP1 and MMPs required for optimal cochlear function (Bhargava et al., 2016). In addition, the balance of MMP9 and TIMP1 expression is thought to affect inflammation and corticosteroid response in patients suffering from autoimmune inner ear disease (Eisner et al., 2017).

### Otoprotection

Nicotinamide phosphoribosyltransferase (NAMPT) was also detected in human perilymph and may present an interesting pharmacotherapeutic target. The NAMPT inhibitor P7C3 leads to otoprotection in an age-related hearing loss model and may emerge in a novel therapeutic strategy for presbycusis (Edu et al., 2017). The ubiquitin carboxyl-terminal hydrolase isozyme (UCHL1) is predominantly expressed in SGC (KimYeon Ju et al., 2019) and may modify the aging process in the auditory cortex by regulating ubiquitin protease system (UPS) -related proteins (Zhang et al., 2017). After gentamicin exposure, UCHL1 is down-regulated and UCHL1-deficiency accelerates gentamycin-induced ototoxicity whereas autophagy delays gentamycin-induced ototoxicity. Stimulating UCHL1 and autophagy (e.g., by rapamycin) attenuates gentamycin-induced apoptosis of auditory hair cells (KimYeon Ju et al., 2019).

Another protein involved in cochlear protection is glutamine synthetase (GLUL), which is expressed in OHCs and SGCs. GLUL may function to limit the perilymphatic glutamate concentrations (Eybalin et al., 1996), as it degrades glutamate and ammonia to the harmless glutamine. Glutamate, which is the most important afferent neurotransmitter in the cochlea and might be important for neuronal signaling between IHCs and SGCs induces neural excitotoxity after acoustic overstimulation (Sun, 1991).

### Potassium Transport and ATP Supply

Some of the proteins expressed in the perilymph are involved in ATP supply, potassium transport and regulation, and are therefore important for the creation and stabilization of the endocochlear potential. Creatine kinase M-type (CKM) is an enzyme expressed in the stria vascularis, spiral ganglion neurons and IHC (Spicer and Schulte, 1992; WongAnn Chi Yan et al., 2012) but is most abundant in hair cell bundles (Shin et al., 2007). The enzyme generates ATP from ADP by catalysing the transfer of a high-energy phosphate from phosphocreatine to ADP. Thus, creatine kinase maintains high ATP levels and is essential for high-sensitivity hearing. Consequently, CK-knockout mice suffer from hearing loss and a vestibular phenotype (Shin et al., 2007; Wallimann et al., 2011). Interestingly, creatine supplementation of healthy-wild type mice significantly reduces noise-induced degeneration of inner and especially outer hair cells and the resulting hearing loss (Minami et al., 2007; Wallimann et al., 2011). Extracellular nucleosides and nucleotides are involved in the molecular response of the cochlea to injury as known from experimental nun human models (Lin et al., 2019b). In this context, an immediate increase of ATP in the extracellular space following injury may be the first step in the initiation of the repair processes in the cochlea (Lin et al., 2019b).

Carbonic anhydrase 3 (CA3), which is expressed in type III fibrocytes of the spiral ligament, is thought to play an active role in both the transport of K^+^ and the regulation of inner ear pH (Weber et al., 2001). It may also be important to facilitate mitochondrial ATP synthesis and detoxifying free radicals resulting from this process (Wu et al., 2013). By mediating the HCO_3_^−^ secretion into the perilymph (Ikeda et al., 1987), carbonic anhydrase regulates from K^+^ transport and recycling, and the generation of the endocochlear potential (Prazma, 1978). Since oxidative stress caused by free radicals resulting from K^+^ transport and recycling, the cochlea needs potent antioxidants. An important antioxidant expressed in the cochlea, isocitrate dehydrogenase 1 (IDH1), is involved in potassium transport in the cochlea and protects proteins in the inner ear from oxidative stress during K^+^ recycling (Kim et al., 2017). IDH1 is downregulated in age-related-hearing-loss (Tadros et al., 2014) and in hearing loss due to chronic lead exposure (Jamesdaniel et al., 2018). Under glucose-6-phosphat-dehydrogenasedeficiency (G6PDH) conditions, the expression of IDH1 is increased to replace the function of G6PDH as the principal source of NADPH for cytosolic antioxidant defence in the cochlea (White et al., 2017).

### Axonogenesis and Cell Adhesion Molecules

Proteins involved in axonogenesis are also found in human perilymph. For example, the protein tyrosine phosphatase receptor type S (PTPRS) is involved in primary axonogenesis and axon guidance during embryogenesis. This tyrosine phosphatase has been also implicated in the molecular control of adult nerve repair.

CEACAM16, a protein expressed in various cells of the organ of Corti (Nishio et al., 2015), connects stereocilia with the tectorial membrane (Kammerer et al., 2012; Cheatham et al., 2014). It may be important for the integrity of the tectorial membrane and is required for proper hearing over an extended frequency range. CEACAM16 interacts with TECTA (Zheng et al., 2011) and is associated with autosomal dominant hearing loss (Nishio et al., 2015).

### Proteomic Approach, Bioinformatic Analysis and Drug Repositioning

As shown in the present study, generating a molecular fingerprint of the inner ear based on human data could accelerate our way to a modern, molecular-based and targeted approach for diagnosing and treating inner ear diseases. As discussed above, several of the individual proteins are involved in cochlear physiology, health and disease. Figure 1 demonstrates that the individual patients show specific patterns of protein expression within the inner ear. In order to understand the clinical significance of these patterns, perilymph from normal hearing individuals will be required. Alternately, these molecular fingerprints combined with functional analysis and localization of the peptide/protein and their druggability could identify potential new targets for hearing impairment therapies. A subset of the proteins identified in the perilymph were assigned to their putative cells of origin and to diverse functional groups as depicted in Figure 4. Based on the proteome profile of the inner ear as identified by perilymph mass spectrometry analysis and the use of an existing platform (i.e., Pharos), we identified several proteins as molecular targets for already existing drugs (Table 1). The quest for such targets has been emphasised in a recent review collecting experimental data on available protein targets for a focused therapeutic delivery to selected cell types within the inner ear (Naples et al., 2020). This is clinically important because the cochlea is protected from external influences by the hard bony otic capsule, round and oval window membrane as well as the blood labyrinth barrier (Naples et al., 2020). In addition, even minimal changes of the cochlear environment can have dramatic impact on the residual health of the cochlea (Naples et al., 2020).

Despite rapid biotechnological progress and significant financial investments in drug development, the rate of launching new drugs is far slower than expected and has remained nearly constant over the last 60 years (Munos, 2009). The most crucial step for the development of new drugs is the discovery of a target gene, protein or enzyme (Ma and Zemmel, 2002). Thus, at the end of the last century, most of the new launched drugs had well-established modes of actions and were directed against already known and clinically validated targets (Ma and Zemmel, 2002; Booth and Zemmel, 2003). Thus, improved trial strategies are possible when the targets are already clinically validated (Booth and Zemmel, 2003). Analyzing key features of overly successful and best-selling drugs, a broad indication range was identified as one of the most important attributes that drive success (Booth and Zemmel, 2003). Providing a human molecular signature of the human inner ear in its diseased or healthy state to be matched with the molecular signature of existing drugs could be one possible way to bridge the gap between animal models and humans. Overall, most drug-target interactions involve inhibitors. As pointed out above, the tissue specific proteins we identified were present only in a subset of our samples. We therefore do not know if their presence is a sign of pathology or not. Application of the identified small molecules to normal inner ear tissue cultures or normal hearing animals could be used to specifically identify the role of these proteins in hearing.

There are some limitations associated with the present study. The analysis was performed on 41 perilymph samples collected from 38 patients. The proteins identified as perilymph specific were not identified in all samples, in fact, some were only identified in a small number of samples. To validate our results, the analysis should be recreated with a greater number of patients and therefore, samples. Analysing an increased number of patients would also allow a better clustering of data. Most of the patients suffered from conditions causing hearing impairment. Therefore, their proteome profile might differ from the proteome of healthy individuals. To our knowledge, there is no information in the literature about how proteins are exactly released into the perilymph. Proteins defined as tissue-specific could also be secreted into the perilymph due to pathological conditions. Since the patients included in the present study suffered from different and sometimes unknown diseases of the inner ear, specific proteins may be present in some but not all patients. We speculate that a significant component of the proteins found in perilymph are associated with extracellular vesicles (microvesicles, exosomes and in the case of dying cells apoptosomes). Identifying the physiological molecular fingerprint of the human inner ear requires further studies and new methods to obtain inner ear samples.

## Conclusion

The perilymph proteome of patients with hearing loss was analysed resulting in identification of a diverse set of proteins that can be targeted with small molecules. This provides us with a baseline list of proteins that may be important for hearing or for response to a hearing loss that can be manipulated using a variety of clinically available or research grade drugs. Combining this information will begin to let us develop a molecular fingerprint of inner ear disease that can be derived from molecular perilymph analysis.

## Data Availability

The original contributions presented in the study are included in the article/Supplementary Material, further inquiries can be directed to the corresponding author.
