# Supplementary material for: Bioinformatic Analysis of the Perilymph Proteome to Generate a Human Protein Atlas
Source: Front Cell Dev Biol. 2022 Apr 28;10:847157. doi: 10.3389/fcell.2022.847157 (PMC9096870; doi:10.3389/fcell.2022.847157)
Supplement: Supplementary file 1 [file Table1.DOCX]

**Localization/ Information about the protein regarding the inner ear**

| # | Gene | Protein | ID | Information about Cochlea | Supposed cell of origin | Protein group | Drug  gability | | Further information |
| --- | --- | --- | --- | --- | --- | --- | --- | --- | --- |
| 4 | CRYM | Ketimine reductase mu-crystallin | Q14894 | Common marmoset: CRYM expression was observed in the lateral wall spiral ligament, both inner and outer hair cells, supporting cells, and the spiral limbus.CRYM expression in the supporting cells was broadly observed between the inner sulcus and outer sulcus cells. CRYM mutations may cause auditory dysfunction through thyroid hormone binding effects on the fibrocytes of the cochlea. (1)mu-Crystallin may be involved in the potassium ion recycling system together with Na,K-ATPase. (2) | lateral region of the **spiral ligament** and the fibrocytes of the spiral limbus (3) | oxidoreductase | Tbio | | DFNA40 (3)  CRYM mutations may cause auditory dysfunction through thyroid hormone binding effects on the fibrocytes of the cochlea. (2) |
| 5 | RARRES1 | Retinoic acid receptor responder 1 | P49788 | Highly expressed in base of mouse cochlea of P8 mice compared with apex (4)  “Cochlea signature gene” (5) | ? | enzyme modulator | Tbio | |  |
| 7 | LPR2 | Low-density lipoprotein receptor related protein 2  (Megalin) | P98164 | Expression corresponding to the apical surface of the **strial marginal cells, to epithelial cells at the spiral prominence and to epithelial cells of Reissner’s membrane** facing the cochlear duct. (6,7)  Required for normal hearing, possibly through interaction with estrogen in the inner ear (Q: Pharos)  Estrogen may function via megalin within the inner ear. A crucial role of megalin in hearing should be considered and the **megalin/estrogen interaction needs to be discussed in the context of early presbycusis in estrogen-deficient humans and mice.** (8) | marginal cells of the stria vascularis, near the apical (endolyphatic) membrane, but not expressed in cochlear hair cells (8) | transmembrane receptor/ membrane traffic protein?? | Tbio | | Donnai Barrow Syndrom  Facio-oculoacoustico-renal syndrome (FOAR)  Megalin-dependant endocytosis of aminoglycosides by marginal cells, i.e. clearance from endolymph could provide a partial otoprotection for hair cells.  (Q: Cellular Mechanisms in Ototoxicity)  A novel role of gp330 as a drug receptor was demonstrated, significance for ototoxic antibiotics? (Q:OMIM) |
| 9 | TTN | Titin  (Isoform 5) | Q8WZ42-5 | In non-muscle cells, seems to play a role in chromosome condensation and chromosome segregation during mitosis. Might link the lamina network to chromatin or nuclear actin, or both during interphase.  (Q: Pharos) | Expressed in Type I spiral ganglion neurons (9) | regulatory/adaptor protein kinase/ **transferase**?? | Tbio | | expressed in congenital cholesteatoma matrices, but not in acquired cholesteatomas (10) |
| 12 | MYH9 | Myosin 9 | P35579 | MYH9 encodes a non-muscle myosin and may be involved in actin degeneration and reorganization of the actomyosin network. (11) | Outer hair cell; Pilar, Supporting, Hensen’s, Claudius’, External and Inner Sulcus Cell; Spiral prominence, limbus and ligament, Reissner’s membrane (12-14) | cytoskeletal protein | Tbio | | non-syndromic sensorineural deafness autosomal dominant type 17 (**DFNA17**) (13)  Epstein syndrome (auch häufig Innenohrschwerhörigkeit), Alport syndrome with macrothrombocytopenia (auch Innenohrschwerhörigkeit!), Sebastian syndrome (eher Blutungskomikationen), Fechtner syndrome (progredienter Hörverlust in 50%) and macrothrombocytopenia with progressive sensorineural deafness  (Q: Pharos) |
| 13 | MFAP4 | Microfibril-associated glycoprotein 4 | P55083 | Signature gene for SG Type 1C neurons, compared with 1A and 1B populations (15)  Upregulated in neonatal mice (P3) (16)  Upregulated in “Atoh-1 induced new hair cells” (17)  Could be involved in calcium-dependent cell adhesion or intercellular interactions. May contribute to the elastic fiber assembly and/or maintenance.  (Q: Pharos) | **Subtype 1C of Spiral Ganglion Neurons** (15) | extracellular matrix protein | Tbio | |  |
| 14 | IVL | Involucrin | P07476 | Expressed in middle ear mucosa (18) |  | cytoskeletal protein ?? | Tbio | | Upregulated in cholesteatoma, further investigation in inhibition may have effects in treatment of cholesteatoma (19) |
| 17 | DNASE1 | Deoxyribonuclease 1 | P24855 | Serum endonuclease secreted into body fluids by a wide variety of exocrine and endocrine organs. (Q:Pharos) | Mouse: Expressed in stria vascularis of cochlear duct, vestibular membrane of cochlear duct, epithelium of cochlear duct  (Q:Bgee) | Hydrolase | Tchem | |  |
| 19 | PRSS8 | Prostasin | Q16651 | Possesses a trypsin-like cleavage specificity with a preference for poly-basic substrates. Stimulates epithelial sodium channel (ENaC) activity through activating cleavage of the gamma subunits (SCNN1G) (Q:Pharos)  Although ENaC is expressed in the developing inner ear, a proposed link between defects in peptidase function and ENaC regulation presently is a matter of speculation. (20) | Mice: Cochlea, vestibular membrane of cochlear duct, cochlear duct of membranous labyrinth, epithelium of cochlear duct, stria vascularis of cochlear duct (Q:Bgee) | Protease | Tchem | | Die entscheidene Protease für die Cochlea scheint Hepsin zu sein, ebenfalls ein möglicher Regulator des ENACs.? (21) |
| 23 | GLUL | Glutamine synthetase | P15104 | Glutamine synthetase that catalyzes the ATP-dependent conversion of glutamate and ammonia to glutamine (Q:Pharos)  L-glutamate was identified adjacent to outer and inner hair cells and in the spiral ganglion. Similar distributions were found for glutamine synthetase (22)  Glutamate is thought to act as a neurotransmitter of the sensory hair cells of the organ of Corti. (23) **Glutamine synthetase may function to limit the perilymphatic glutamate concentrations**. (24) | Human: basal part of OHC- area, in spiral ganglion around almost the entire plasma membrane (22) | Ligase/ transferase | Tchem, but 2 active drugs |  | |
| 24 | CRYAB | Alpha-crystallin b-chain (Fragment) | E9PR44  P02511 | Upregulated in adult mice compared with neonatal mice (25)  Here, we tested the oto-protective properties of the small heat shock protein alpha B-crystallin (HspB5) with previously reported **anti-inflammatory, anti-apoptotic and neuroprotective functions, in an experimental model of PM-induced (Pneumococcal meningitis) hearing loss.** These results suggest that **high local concentrations of HspB5 are needed to prevent inner ear damage in acute PM. HspB5 represents a promising therapeutic option to improve the auditory outcome and counteract hearing loss after PM.**  (26) |  | Chaperone | Tbio | ID-> no entry in Pharos, UniProt: Obsolete, Information through protein name | |
| 27 | CKAP4 | Cytoskeleton-associated protein 4  (CLIMP63 | Q07065 | High-affinity epithelial cell surface receptor for antiproliferative factor (APF). Mediates the anchoring of the endoplasmic reticulum to microtubules. (Q: Pharos) | Expressed in surrounding cells in cochlea and vestibular organ (27)  Organ of corti (28) | transmembrane receptor | Tbio | CLIMP-63 dimerization is associated with gentamicin-induced cytotoxicity. (28) | |
| 28 | PGM1 | Phospho-glucomutase 1 | P36871 | Increases in expression were observed after gentamycin + cisplatin exposure (29) | Highly enriched in non-sensory cells versus vestibular hair cells (30) | isomerase | Tbio | SNPs Associated with Cisplatin IC50., Expression differs with different polymorphisms (31) | |
| 29 | BPI | Bactericidal permeability-increasing protein | P17213 | BPI injection prevented disturbance of the mucociliary clearance system of the middle ear. Hence, it is postulated that BPI can be a new therapy for chronic otitis media with effusion (OME) (32) | Middle ear? | Defense/Immunity Protein | Tbio | Middle ear mucosa recovers from inflammatory changes associated with OME after treatment with rBPI21. This suggests that rBPI21 may be useful in the treatment of OME and of mucosal infections of the respiratory tract (33) | |
| 30 | SUMO3 | Small ubiquitin-related modifier 3 | P55854 | The encoded protein is covalently conjugated to other proteins via a post-translation modification known as sumoylation (Q:Pharos)  Important for Thyroid Hormone Receptor β- Induction, which is required for normal cochlear development (34) |  | enzyme modulator? | Tbio |  | |
| 31 | TGM2 | Protein-glutamine gamma-glutamyltransferase 2 | B4DIT7  P21980 | Catalyzes the cross-linking of proteins and the conjugation of polyamines to proteins. (Q:Pharos) | Expressed in surrounding cells (27) | transferase | Tchem | Unter der Uniprot ID: cDNA FLJ58187, highly similar to Protein-glutamine gamma-glutamyltransferase 2  Transglutaminase- Inhibitor Patent:  <https://patents.google.com/patent/WO2012108841A1/en> | |
| 32 | MDH2 | Malate dehydrogenase | G3XAL0 | Malate dehydrogenase catalyzes the reversible oxidation of malate to oxaloacetate, utilizing the NAD/NADH cofactor system in the citric acid cycle. (Q:Pharos) | Expressed in cochlea, but more abundant in Utricle (35) | oxidoreductase | Tchem | ID-> no entry in Pharos, but through gene name  Candidate Gene for DFNB39 (no gene indentified) (36) | |
| 33 | P4HB | Protein disulfide-isomerase | P07237 | This multifunctional protein catalyzes the formation, breakage and rearrangement of disulfide bonds. At high concentrations, functions as a chaperone that inhibits aggregation of misfolded proteins. At low concentrations, facilitates aggregation (anti-chaperone activity). (Q:Pharos) Seems to be involved in regulation of calcium- induced calcium release, protein folding and clearance🡪 involved in regulation of pro- apoptosis in mouse cochlear hair cells.(Q: Current Understanding of Apoptosis: Programmed Cell Death) | Cochlear protein or protein with small, but significant cisplatin-induced changes in expression level (37) | Chaperone, Isomerase | Tbio |  | |
| 34 | CD9 | CD9 antigen | A6NNI4 | “otic lineage-specific”  = relative differences between total otic vs. non-otic expression of genes. (38) | Expressed in deiters cells (39) | transmembrane receptor | Tbio | ID-> no entry in Pharos, but through gene name | |
| 36 | MSLN | Mesothelin (Fragment) | H3BUX1 | Shows weak homology with otoancorin, a protein expressed on the apical surgace of the epithelial cells of the inner ear, which might mediate the attachment of the tectorial membrane (Q: Cochlea Implants: Principles & practice), OTOA mutations cause DFNB2. (40)  Mesothelin and mesothelin precursor proteins are remotely homologous to stereocilin and otoancorin and more closely homologous to the hypothetical protein MPFL (MPF-like). We suggest that all of these function as superhelical lectins to bind the carbohydrate moieties of extracellular glycoproteins. (41) |  | cell adhesion molecule | Tbio | ID-> no entry in Pharos, but through gene name  Several antibody-based therapeutic agents as well as vaccine and T-cell therapies directed at mesothelin are undergoing clinical evaluation. (Q: <https://ascopubs.org/doi/10.1200/JCO.2016.68.3672> ) | |
| 38 | TRIM29 | Tripartite motif-containing protein 29 (Isoform Beta) | Q14134-2 | Upregulated in rat cochlea 28 days after blast overpressure exposure (42)  Regulation of macrophage activation in response to viral or bacterial infection. Induces ubiquitination of Lys48. May act as a transcriptional regulatory factor involved in carcinogenesis and/or differentiation. (Q:Pharos) |  | **defense/immunity protein**  nucleic acid binding/ transcription factor? | Tbio | The results showed that TRIM29 knockdown significantly attenuated while TRIM29 overexpression promoted nasopharyngeal carcinoma (NPC) cell in vitro proliferation, migration and invasion and in vivo metastasis. (43)  Information is not isoform-specific! | |
| 39 | LXN | Latexin | Q9BS40 | On List of Cochlear signature genes detected on macaque array chip platform. (5)  Hardly reversible, non-competitive, and potent inhibitor of CPA1, CPA2 and CPA4. May play a role in inflammation.mhcöstroge (Q:Pharos) |  | enzyme modulator | Tbio |  | |
| 40 | MUC5B | Mucin-5B | Q9HC84 | Gel-forming mucin (Q:Pharos)  Mucin 5B and aquaporin 5 gene expression were associated with effusion viscosity and Mucin 5B with middle ear epithelial thickness and hearing loss. (44) Muc5b, but not Muc5ac, is required for mucociliary clearance (MCC), for controlling infections in the airways and middle ear (45) | Middle ear mucosa,  the MUC5B mucin gene and its product were identified in the middle ear secretory cells of patients with Chronic Otitis media (COM). **Its expression was extensive in pseudostratified mucosal epithelia and related to infiltration of inflammatory cells in the submucosa of the middle ea**r cleft with COM, suggestive that inflammatory cell products are involved in the production of MUC5B. (46,47) | extracellular matrix protein | Tbio |  | |
| 42 | DBI | Acyl-CoA-binding protein | P07108 | Induction of *Dbi*, which is believed to down-regulate GABA-mediated inhibitory responses, might represent a mechanism to increase neuronal activity in either hair cells or spiral ganglion neurons to compensate for decreased signals from nonfunctional hair cells. (48)  Is thought to downregulate the effects of GABA (Q:OMIM) | Hair cell or spiral ganglion | Transporter | Tbio | Identified *Aim1*, *Dbi*, and *Tm4sf3*as genes with increased expression in *sh2/sh2* homozygotes. (48)  (Mutation in MYOXV Gene) | |
| 43 | SDCBP | Synthenin (Isoform3) | O00560-3 | Multifunctional adapter protein involved in diverse array of functions including trafficking of transmembrane proteins, neuro and immunomodulation, exosome biogenesis, and tumorigenesis | Found in Hair cells (GFP +), but not in Inner Ear Samples or Sensory epithelia dataset (Hair-Cell-only) (49) | signaling molecule/ **membrane traffic protein**/ cell junction protein?? | Tbio | Stable gene (=no change in expression) after acoustic trauma. (50)  Information not isoform specific. | |
| 44 | CEACAM16 | Carcinoembryonic antigen-related cell adhesion molecule 16 | Q2WEN9 | Glycoprotein that interacts with TECTA. (51) CEACAM16 may have a role in connecting stereocilia with the tectorial membrane (52,53) ckm  (Q:Pharos) | Inner and Outer Hair Cell, Pillar, Supporting and Inter Dental Cell, Tectorial Membrane  Stereocilia Tips (11) | cytoskeletal protein | Tbio | AD-NSHL (11)  DFNA4? (Q:Pharos) | |
| 45 | COL2A1 | Collagen alpha 1 (II) chain (Isoform1) | P02458-1 | Structural components of the extracellular matrix of chondrocytes and the tectorial membrane. (11,54) | Tectorial Membrane, Spiral limbus (11,54)  Only connective tissue structures including spiral ligament, spiral limbus, tectorial and basilar membranes, modiolar and spiral lamina cartilage contained type II collagen. (55) | extracellular matrix protein | Tbio | Stickler syndrome 1, STL I, associated with hearing impairment in 52.2%. (56)  Information not isoform specific. | |
| 47 | CKM | Creatine kinase M-type | P06732 | Marginal cells of the cochlear stria vascularis and dark cells and transitional cells of the vestibular system were found to contain an abundance of the MM isozyme (MM- CK).CK in these cells concurs with that which is coupled to Na,K-ATPase in other cells and is considered to supply ATP for the Na,K-ATPase that mediates the high KCl of endolymph. Inner hair cells revealed content of the BB isozyme and in this respect resembled the energy-transducing photoreceptor cells in retina. In addition, outer phalangeal (Deiters’) cells stained for both MM-and BB-CK whereas inner phalangeal cells evidenced content of only the BB isozyme. (57) | low level of CKM immunoreactivity in the spiral ganglion neurons and inner hair cells in the adult mouse and gerbil cochleae was found (57,58) | transferase | Tbio | Thus, diffusion limitations of ADP and ATP are overcome by PCr/Cr shuttling, as most clearly seen in polar cells such as sensory hair bundles of the inner ear. CK knockout mice presented with hearing loss and a strong vestibular phenotype (59,60). Interesting in this context is the fact that Cr supplementation of healthy wild-type mice significantly attenuates noise-induced destruction of inner and more so of outer hair cells and the concomitant hearing loss (60,61) | |
| 49 | COL11A2 | Collagen alpha-2 (XI) chain | Q6ZNN4 | The expression patterns suggest essential roles for Col11a1 and Col11a2 in the basilar or tectorial membranes. (62)  Structural components of the extracellular matrix of the tectorial membrane. (11) | Hensen’s and Claudius’ Cell, Spiral Ligament, Stria Vascularis, Spiral Limbus (11) | extracellular matrix protein | Tbio | ID-> no entry in Pharos, Information through protein name.  Mutations in this gene are associated with type III Stickler syndrome, otospondylomegaepiphyseal dysplasia (OSMED syndrome), Weissenbacher-Zweymuller syndrome, autosomal dominant non-syndromic sensorineural type 13 deafness (DFNA13) (63), and autosomal recessive non-syndromic sensorineural type 53 deafness (DFNB53) (Q:Pharos) | |
| 50 | FHL1 | Four and a half LIM domains protein 1 | Q13642-1 | Downregulated in avian utricles and cochleae 48h after neomycin exposure (64)Iinvolved in many cellular processes (Q:Pharos) |  | Unknown | Tbio | Information not isoformspecific | |
| 51 | CA3 | Carbonic anhydrase 3 | P07451 | Therefore, CAIII may play dual roles in otic fibrocytes: facilitating mitochondrial ATP syn- thesis as well as detoxifying free radicals resulting from active ATP synthesis. (65) We postulate that the HCO3− secretion into the endolymph is mediated by carbonic anhydrase (CAH) and is decreased by acetazolamide (ACZ) due to the inhibition of CAH. The endocochlear potential, at 81.6 ± 1.5 mV under normal conditions, was reduced by 4.9 ± 0.9 mV in 30 min following the ACZ injection (66) It was observed for the first time that carbonic anhydrase affects the generation of EP. (67) | Anti-CA III stained intensely type I and type III fibrocytes in the spiral ligament. This finding, in conjunction with the distribution patterns for Na,K-ATPase and NKCC, suggests an active role for spiral ligament fibrocytes in both the transport of K+ and the regulation of inner ear pH. (68) | lyase | Tclin |  | |
| 54 | ATP5F1A | ATP synthase subunit alpha, mitochondrial (Fragment) | ATP5F1A | Seems to be an inner ear-specific interaction partner of LMO7 (component of the cuticular plate) (69)  Alpha subunit of the catalytic core, F1 |  | Transporter | Tbio | ID->no entry in Pharos, but through gene name | |
| 55 | SPINT1 | Kunitz-type protease inhibitor 1 (Fragment) | H3BTQ8 | Seems to be part of supporting cell genes which are enriched in P1 (70)  Inhibitor of matriptase (ST14), inhibitor specific for HGF activator. (Q:Pharos) | Expressed in embryonic and postnatal hair cells (27) | enzyme modulator | Tbio | ID->no entry in Pharos, but through gene name | |
| 56 | AGA | Asparty-lglucosaminidase | P20933 | Involved in the catabolism of N-linked oligosaccharides of glycoproteins (Q:Pharos) |  | Protease/ hydrolase | Tbio | Mutation in this gene cause severe vacuolation of the cells in vestibular and cochlear Nuclei (u.a.) (71)  Aspartylglycosaminuria, seems to be associated with recurrent respiratory and ear infections.  (Q: livingagu.com) | |
| 57 | ARPC4 | Actin-related protein 2/3 complex subunit 4 | F8WDD7 | mRNA found in inner ear,  identified in inner ear EST database (72), involved in cell motility (Q:Pharos) |  | cytoskeletal protein | Tbio | ID-> no entry in pharos, but through gene name  Downregulated in developing rat cochlea at E6 (73) | |
| 58 | TPM3 | Tropomyosin alpha-3 chain | Q5VU66 | TPM3, variant 1 expressed in cochlea, same as NM-1? (=non-muscle)  Binds to actin filaments in muscle and non-muscle cells. (Q:Pharos) |  | cytoskeletal protein | Tbio | Information about Tropomyosin, not specific for TMP3: Tropomyosin, which stabilizes actin filaments, is present in all the actin filament assemblies except for the stereocilia. (74) Tropomyosin colocalizes with actin and tubulin in the regions of the tunnel pillar and Deiters cells (75) present within stereocilia rootlets | |
| 60 | GPHA2 | Glycoprotein hormone alpha-2 | Q96T91 | Alpha subunit of glycocoprotein hormone (not 2): Change in expression in murine cochlear culture in the presence of dexamethasone in the cochlear culture (76) |  | signaling molecule | Tbio |  | |
| 63 | TYMP | Thymidine phosphorylase | C9JGI3 | Mitochondrial neurogastrointestinal encephalomyopathy (MNGIE syndrome): loss-of-function mutations in TP cause the disease (77,78)  Associated with hearing loss in 61% (77) Catalyzes the reversible phosphorolysis of thymidine, angiogenic factor (Q:Pharos) |  | transferase  signaling molecule | Tclin | ID->no entry in Pharos, but through gene name  Otolaryngologists should be aware of mitochondrial neurogastrointestinal encephalomyopathy and other rare genetic disorders when managing patients with progressive hearing loss (79)  Review über das Krankheitsbild und vorhandene Therapieoptionen (80) | |
| 65 | GBA | Glucosylceramidase | J3KQG4 | Mutations in this gene cause Gaucher disease, a lysosomal storage disease characterized by an accumulation of glucocerebrosides, with case reports of sensoneurinal hearing loss (81)  Glycolipid metabolism: hydrolysis of glucosylceramide/GlcCer into free ceramide and glucose (Q:Pharos) |  | Hydrolase  transferase | Tclin | Mutations in (u.a.) GBA are associated with microtia-atresia (82) | |
| 67 | SERPINB2 | Plasminogen activator inhibitor 2 | P05120 | Overexpression of individual AID (Activity-regulated Inhibitors of Death) genes is neuroprotective both in vitro and in vivo, which also is relevant for the auditory system, where ablation of the cochlea or transection of the eighth nerve causes [neuronal cell death](https://www.sciencedirect.com/topics/neuroscience/neuronal-cell-death) in the [cochlear nucleus](https://www.sciencedirect.com/topics/neuroscience/cochlear-nucleus) or nucleus magnocellularis in mammals or chick, respectively (83) |  | enzyme modulator (Serin protease inhibitor) | Tbio |  | |
| 69 | TLR3 | Toll-like receptor 3 | E9PGH4 | Key component of innate and adaptive immunity. (Q:Pharos) Not detectable in organ of corti cells under physiological condition, but increased TLR3 protein expression is associated with sensory cell damage. Increased TLR protein expression is an early event during cell degeneration. (84,85) | Expressed in human endolymphatic sac fibroblasts (86) | defense/immunity protein  receptor  nucleic acid binding | Tbio |  | |
| 70 | BCAP31 | B-cell receptor-associated protein 31 | P51572 | chaperone protein, multi-pass transmembrane protein of the endoplasmic reticulum that is involved in the anterograde transport of membrane proteins from the endoplasmic reticulum to the Golgi. | Highly expressed immune/inflammation-related gene in the normal cochlear sensory epithelium (85) | membrane traffic protein  enzyme modulator  chaperone | Tbio | Mutations can be associated with deafness, dystonia and cerebral hypomyelination (case report) (87) | |
| 74 | HNRNPK | Heterogeneous nuclear ribonucleoprotein K | Q5T6W5 | Case report: There were persistent middle ear effusions during childhood, and he currently has mixed conductive and sensorineural hearing loss + underdeveloped helices (88)  Pre-RNA binding, also transcriptional coactivator (Q:Pharos) |  | transcription factor  **nucleic acid binding** | Tbio | ID-> no entry in Pharos, UniProt: obsolete, Information through protein name | |
| 75 | FTL | Ferritin light chain | P02792 | Present in normal cochlea, downregulated after exposure with ototoxic agent ouabain (89)  Indentified from mouse inner ear (72) | Expressed in low levels in cuticulosomes (=iron rich organelles in bird cochleas) (90) Iron ions are stocked in the stria vascularis (ferritin depots).  The expression of ferritin (91) and the concomitant apical expression of DMT1 suggest that the stria vascularis has an active role in restoration and deposition of iron, with particular attention to reducing the endolymphatic concentration of divalent ions (92) | Iron storage protein | Tbio |  | |
| 76 | GSTM2 | Glutathione S-transferase Mu 2 | E9PHN7 | Female CBA/CaJ mice displayed higher mRNA expression in inner ear tissue of nine genes involved in Phase II detoxification, including Gsta4, Gstm2, and Gstt1, compared to males, which is probably otoprotective. One of 25 gene downregulated in phase II detoxification after ovariectomy.  🡪 Production is östrogen dependant (93) |  | transferase | Tchem | ID->no entry in Pharos, but through gene name | |
| 79 | PLP2 | Proteolipid protein 2 | Q04941 | Evaluation of hearing thresholds revealed a moderate pantonal hearing loss across all frequencies in PLP null*M6Bnull -mice, auditory signal propagation was delayed. (94) Upregulated 1 day after noise exposure (95) | Expressed in surrounding cells (27)  Enriched in type II spiral ganglion neurons (96) | membrane traffic protein | Tbio | In endoplasmatic reticulum, may function as an ion channel | |
| 80 | LAMB1 | Laminin subunit ß-1 | P07942 | Candidate gene (differentially expressed by >1,5-fold) for nonsyndromic human deafness interval for which no causative gene has been identified to date: **DFNB14** (36) | Expressed by surrounding cells (27) | cell adhesion molecule | Tbio | Alport syndrome features ectopic deposition of LAMA2**, LAMB1**, and COL4A112 in the GBM, which could be compensatory and/or pathogenic (97)  Required for the integrity of the basement membrane. (Q:Pharos) | |
| 81 | SERPINB8 | Serpin B8 (Fragment) | H7BXK7 | Upregulated one day after noise exposure in Cochlea (95) |  | enzyme modulator (Serin protease inhibitor) | Tbio | ID->no entry in Pharos, but through gene name  Cell adhesion | |
| 82 | PEBP4 | Phosphatidyl-ethanolamine-binding protein 4 | Q96S96 | MiR-15b, directly targeting phosphatidylethanolamine-binding protein 4(PEBP4), can both promote Cisplatin resistance and epithelial-mesenchymal transition. (98,99) (Especially in cancer cells, significance for ototoxicity?) |  | enzyme modulator (Serin protease inhibitor) | Tbio | Promotes cellular resistance to TNF-induced apoptosis (Q:Pharos) | |
| 83 | PLP1 | Myelin proteolipid protein | P60201 | Major myelin protein from the central nervous system (Q:Pharos). Transient loss of cochlear Schwann cells results in permanent auditory deficits characteristic of Hidden hearing loss (100)  The spiral ganglion comprises the cell bodies of cochlear afferents and Schwann cells, and only the Schwann cells are PLP1+. In mature rodents, Schwann cells myelinate the peripheral processes of the bipolar spiral ganglion neurons, their cell bodies and the initial portions of their central axons ([Hurley et al., 2007](https://www.ncbi.nlm.nih.gov/pmc/articles/PMC5201044/#DEV139840C45); [Spoendlin, 1975](https://www.ncbi.nlm.nih.gov/pmc/articles/PMC5201044/#DEV139840C93); [Toesca, 1996](https://www.ncbi.nlm.nih.gov/pmc/articles/PMC5201044/#DEV139840C94)) up to the glia limitans. PLP1 is a marker for spiral ganglion progenitors (101) Auditory signal propagation was delayed in PLPnull*M6Bnull-mice. (94) | In the neonatal mouse cochlea (P1–P15) Plp1 is expressed by Schwann cells, satellite cells, as well as inner phalangeal and inner border cells (IPhC/IBCs), the supporting cells surrounding IHCs. (100) | membrane traffic protein | Tbio |  | |
| 84 | CAND1 | Cullin-associated NEDD8-dissociated protein 1 | Q86VP6 | Exclusively expressed in P3 in mice cochlear sensory epithelia (top 25 proteins with the largest mean normalized spectral counts for this age) (102) |  | enzyme modulator | Tbio | involved in ubiquitinylation of proteins degraded by the Ub proteasome system (Q:Pharos) | |
| 85 | TAGLN2 | Transgelin 2 | P37802 | Upregulated one day after noise exposure (95) | Expressed in surrounding cells (27) | Unknown | Tbio | Function not determined, thought to be a tumor supressor | |
| 86 | GPRC5B | G-protein coupled receptor family C group 5 member B | Q9NZH0 |  | Enriched in vestibular ganglion in comparison with spiral ganglion on embryonic day 12 (103) | transmembrane receptor | Tbio | Function not determined | |
| 88 | MNDA | Myeloid cell nuclear differentiation antigen | P41218 |  | Expressed as a Damage-associated molecular pattern (DAMP) in inner and outer hair cells (104) | transcription factor  nucleic acid binding | Tbio | a transcriptional activator/repressor in the myeloid lineage (Q:Pharos) | |
| 89 | FN3K | Fructosamine-3-kinase | Q9H479 | Catalyzes the phosphorylation of fructosamines | Expressed in embryonic and postnatal hair cells. (27)  Enriched gene in type-I spiral ganglion cells. (96) Marker gene of inner ear hair cells. (105) | regulatory/ adaptor protein kinase  Transferase/ kinase | Tbio |  | |
| 90 | LDB1 | LIM domain-binding protein 1 (Isoform 2) | Q86U70-3 | The prosensory-formation potential within the ventral cochlear epithelium was controlled through LDB1-mediated interaction of ISL1and GATA3. CoIP experiments showed that LDB1 did not act as a bridge between ISL1 and GATA3 to form a protein complex. (106)  LDB1 is a known target or binding partner of LMO4 (107), which is downregulated by cisplatin (108), but LDB1 shows no concentration change induced by cisplatin (107). | From E12.5 to E14.5, Ldb1 was ubiquitously expressed in the cochlea and the surrounding mesenchyme. (106) | Developmental protein | Tbio | No isoformspecific information | |
| 91 | HSPA4 | Heat shock 70 kDa protein 4 | P34932 | HSP70 is secreted by supporting cells. Together our data indicate that supporting cells mediate the protective effect of HSP70 against hair cell death, and they suggest a major role for supporting cells in determining the fate of hair cells exposed to stress. (109) We have shown that HSP induction via heat shock inhibits aminoglycoside-induced hair cell death in organ cultures of utricles from adult mice (110)  HSP70 is required for this protective effect, and HSP70 overexpression inhibits ototoxic hair cell death (111)  GA induced HSP70 in the auditory sensory cells and partially protected them from toxicity of gentamicin.  (112)  HSP70 antibodies have been reported in many inflammatory diseases and may mark the early onset of hearing loss. Indeed, several studies have shown that HSP70 antibodies are less prevalent in individuals with inactive or stable disease (113) | HSP70-like immunoreactivity was observed in Claudius' cells and in the interdental cells of the spiral limbus. In the organ of Corti, immunoreactivity was confined to pillar cells, as well as Hensen's and Deiters' cells. (114) | Stress response | Tbio | Because of the high prevalence of anti‐HSP70 antibodies in healthy subjects and the very limited association of anti‐HSP70 antibody status with clinical features or course of Meniere's disease, we conclude that, at present, the detection of anti‐HSP70 antibodies by Western blotting offers little clinically useful information in Meniere's disease. (115)  HSP70 subtype 4 (detected in the perilymph of one patient) and Alpha-crystalline B chain (detected in two patients) were rarely present in the perilymph on the patient cohort investigated in the present work. (116)  Information is about HSP70!!  Same as HSPA4?? | |
| 92 | HMGB1 | High mobility group protein B1 | Q5T7C4 | Spatiotemporal expression of HMGB1 in the developing mouse cochlea suggests that it may play an important role in cochlear development and hearing (Q: Expression and Distribution of High Mobility Group Box 1 (HMGB1) during Cochlear Development in Postnatal Mice)  HMGB1 can positively influence the survival of SGNs following ototoxic exposure via both its extracellular and intranuclear functions (117) In stressful conditions, Deiters cells liberate HMGB 1 to regulate the epithelial reorganization of the injured organ of Corti through engagement of RAGE in neighbouring epithelial cells. (118) HMGB1 is over-expressed in chronic middle-ear pathologies (119) | Localization of HMGB1 was observed in the nucleus throughout postnatal development, widely distributed in multiple types of cells, including sensory hair cells and supporting cells in the organ of Corti, the stria vascularis, spiral ligament, microvessels within the cochlear lateral wall, spiral limbus, spiral ganglion neurons and glial cells (Q: Expression and Distribution of High Mobility Group Box 1 (HMGB1) during Cochlear Development in Postnatal Mice) | defense/ immunity protein  signaling molecule  nucleic acid binding | Tbio | The concentrations of HMGB-1 and IL-10 in the hydrogen-saturated saline group were significantly higher than in those in the normal saline group immediately and at 7 d after noise exposure 🡪 protective effects of hydrogen-saturated saline on noise-induced hearing loss (NIHL) are related to both the anti-oxidative activity and anti-inflammatory activity. (120)  Expressed in cholesteatomas. (121) | |
| 96 | CDH11 | Cadherin 11 (Isoform 2) | P55287-2 | Cadherin-11/Cdh11 is expressed through early development and strongly during inner ear development (otic placode and vesicle). Inner ear defect resulting from cadherin-11 gene (cdh11) loss-of-function: reduced and absent otoliths. (122) CDH11 knockout mice showed reduced acoustic startle responses and increased thresholds for auditory brainstem responses, indicating moderate hearing loss, conductive, CDH11 might be involved in middle ear cavitation (123) | Present in supporting cells of the basal papilla, involved in the formation of chicken tectorial membrane (Q: Molecular cloning and expression analysis of ADAMs and cadherins during chicken embryonic development) | calcium-binding protein  cell adhesion molecule | Tbio | Elsahy Waters syndrome (inter- and intrafamilial variability regarding the presence of hearing loss) (Q:Pharos/ OMIM)  Information not isoform specific | |
| 97 | NID2 | Nidogen-2 (Isoform 2) | Q14112-2 | NID2 is also a good candidate for DFNA23. NID2 has also been shown to bind to collagen I. Mutations in COL1A1 have been shown to cause osteogenesis imperfecta congenita with sensorineural and/or conductive hearing loss. (124) may have a role in maintaining the structure of the epithelia in close proximity to the attachment site of the tectorial membrane. (125) | Expressed in surrounding cells (27)  Immunostaining for nidogen-2, a basement membrane component encoded by *NID2*, was most prominent in the epithelial lining of the inner spiral sulcus between the tectorial membrane and the inner hair cell as well as localizing to nerve fibers and blood vessel basement membranes. (125) | cell adhesion molecule | Tbio | Information is not isoform specific | |
| 99 | CYBB | Cytochrome b-245 heavy chain  Alternative name:  NADPH OXIDASE 2; NOX2 | F5GWD2 | We demonstrate that Ginkgolide B decreases ROS generation through reducing NOX2 expression and enhancing activity through Akt-Nrf2-HO-1 pathway, resulting in inhibition of mitochondrial apoptosis and final reduction of cisplatin-induced ototoxicity in vitro and in vivo (126) | p91phox/NOX2 mRNA, typically expressed in phagocytes, could also be detected from inner ear by RT-PCR  (127) | membrane traffic protein (ion channel)  oxidoreductase | Tchem | ID-> no entry in Pharos, UniProt: obsolete, Information through protein name | |
| 101 | IDH1 | Isocitrate dehydrogenase [NADP] cytoplasmic | O75874 | We postulate that the IDH family participates in transporting K+ ions in the cochlea and functions to protect proteins in the inner ear from oxidative stress during K+ recycling. (128) Downregulated in age related hearing loss mice (129) IDH1 is downregulated in cochlea by lead exposure. These changes collectively suggest that chronic exposure to lead could weaken the cochlear anti- oxidant defense machinery and thereby enhance the susceptibility of the inner ear to oxidative damage. (130) | IDH1 was detected throughout the cochlea (128) | oxidoreductase | Tclin | In a mouse inner ear cell line, knockdown of *Idh1*, but not *G6pd*, decreased cell growth rates, cytosolic NADPH levels, and thioredoxin reductase activities. Therefore, under normal physiological conditions, *G6pd* deficiency does not affect the cytosolic glutathione or thioredoxin antioxidant defense in mouse cochlea. Under *G6pd* deficiency conditions, isocitrate dehydrogenase 1 likely functions as the principal source of NADPH for cytosolic antioxidant defense in the cochlea. (131) | |
| 106 | RAC2 | Ras-related C3 botulinum toxin substrate 2 (Fragment) | B1AH78 | Upregulated 1 day after noise exposure (95) |  | signaling molecule  small GTPase | Tbio | ID-> no entry in Pharos, but through gene name  RAC2-Mutation: absence of sensorineural hearing loss. In murine models, RAC1 and RAC3 (but not RAC2) are involved in the development of the inner ear (132); however, this has yet to be confirmed in human studies. (133) | |
| 108 | ACLY | ATP-citrate synthase | B4E3P0 | ATP-citrate synthase is the primary enzyme responsible for the synthesis of cytosolic acetyl-CoA in many tissues. . In nervous tissue it may be involved in the biosynthesis of acetylcholine. (Q:Pharos) | Either hair cell specific or strongly upregulated in hair cells compared to non-sensory cells. (30) | transferase | Tclin |  | |
| 109 | ARF1 | ADP ribosylation factor 1 | P84077 | One vesicle pool associates with ADP-ribosylation factor 1 (Arf1)-positive vesicles, colocalizes with the endosomal GTPase, rab5, and is trafficked to the apical aspect of cochlear hair cells. (134) | In mouse utricle extracts, we detected the ARF family members ARF1-4 (135) | Transporter | Tchem | Vesicle transport and protein trafficking (Q:Pharos) | |
| 110 | UCHL1 | Ubiquitin carboxyl-terminal hydrolase isozyme L1 | D6RE83 | Downregulated after gentamycin exposure. A deficit in Uchl1 accelerated GM-induced ototoxicity by showing a decreased number of SGCs and nerve fibers in organotypic cochlear cultures and HEI-OC1 cells. UCHL1-dependent autophagic flux may have a potential as an otoprotective target for the treatment of GM-induced auditory cell death. (136) | Cochlear tissue showed a predominant expression of UCHL1. UCHL1 was predominantly expressed in SGCs, as well as efferent nerve terminal and nerve fibers connected to the HCs from the SGCs. (136)  Enriched in supporting cells on postnatal day 1 and 6 mice. (70) | protease  hydrolase | Tchem | ID-> no entry in Pharos, but through gene name  UCHL1 may modify the aging process in the auditory cortex by regulating UPS- related proteins. (137) Enriched in Atoh1-expressing hair cells, GO analysis suggests it is involved in biological process associated with sensory organ development (138) | |
| 111 | ACTR3 | Actin-related protein 3 | F5H3P5 | ATP-binding component of the Arp2/3 complex, a multiprotein complex that mediates actin polymerization (Q:Pharos). 400 copies per stereocilium from purified chick vestibular hair bundle (139) | Expressed in non-sensory cells (incl. surrounding cells) but not in vestibular hair cells (30) | cytoskeletal protein | Tbio | ID-> no entry in Pharos, UniProt: obsolete, Information through protein name | |
| 112 | TIMP1 | Metalloproteinase inhibitor 1 | Q5H9A7 | Biphasic expression change in the Timp1 protein with an early downregulation and a subsequent upregulation during sensory cell degeneration. Inhibits MMP, which might participate in cochlear response to acoustic overstimulation and can serve as a novel therapeutic target. (140)  Thus it is evident that hearing loss due to several causes— HHCY or noise, antibiotics or infection/inflammation—is mediated through altered expression of the MMPs specifically 2 and 9 (Fig. 2). Hence, it could be modified by administration of MMP inhibitors (in case of noise in the first month after exposure). At the same time, we must keep in mind that administering these inhibitors in the normal state (without HHCY) can also cause hair cell death in the cochlea. This would require further studies on the optimum concentration of MMPs required for normal functioning in the cochlear endolymph and the specific MMP inhibitors that may be used to attenuate hearing loss. (141) | In the normal cochlea, Timp1 immunoreactivity was weakly present in the cytoplasm of the sensory cells and the supporting cells (140)  Expressed in healthy stria vascularis | signaling molecule  enzyme modulator (Inhibitor) | Tbio | Balance of TIMP-1 and MMP-9 may influence inflammation and corticosteroid response in autoimmune inner ear disease (AIED) patients. How to enhance expression of TIMP-1 may confer a protective phenotype which correlates with clinical corticosteroid responsiveness. (142) | |
| 113 | PSMA2 | Proteasome subunit alpha type 2 | P25787 | “cochlear signature gene” detected on macaque or human array chip platform (=expression levels more than 2-fold greater than in the other tissues) (5)  Component of the proteasome (Q:Pharos) |  | protease  hydrolase | Tbio |  | |
| 114 | LMNB1 | Lamin-B1 | P20700 | LaminB1 was expressed in the developing cochlea and developmentally regulated in cochlear tissues, suggesting a possible role of LaminB1 in cochlear development. (143) | outer hair cells, inner hair cells, Kolliker's organ, Reissner's membrane, Spiral ganglion cells (SGC), stria vascularis (STV), and spiral ligament. (143) | Maintain nuclear shape and mechanical integrity | Tbio | Component of nuclear lamina, may interact with chromatin (Q:Pharos) | |
| 115 | SET | Protein SET, Isoform 3 | Q01105-3 | In the interactome of chicken cochlea one of the 8 major hubs, containing a central protein connected to six or more partners, some of which are linked to the larger global network. (144)  Downregulated in inner ear of Atlantic salmon Salmo salar following exposure to a seismic airgun (145) |  | nucleic acid binding  chaperone | Tbio | Information not isoform specific. Multitasking protein, involved in apoptosis, transcription, nucleosome assembly and histone chaperoning. (Q:Pharos) | |
| 116 | ASAH1 | Acid ceramidase | E7ERV9 | A specific form of ASAH1-related disorder is called spinal muscular atrophy with progressive myoclonic epilepsy (SMA-PME), some patients present sensorineural hearing loss, which is hearing loss that occurs when the nerves within the ear cannot properly send sensory input (sound) to the brain, and is not caused by problems with the ear itself. (Q: rarediseases.org) (146,147) |  | hydrolase | Tchem | ID-> no entry in Pharos, UniProt: obsolete, Information through protein name  Hydrolyzes the sphingolipid ceramide into sphingosine and free fatty acid (Q:Pharos) | |
| 117 | RAB11A | Ras-related protein Rab-11A (Fragment) | H3BMH2 | The GTPase Rab11a that has been shown to mark the basal region of primary cilia and to be critical for primary ciliogenesis (148) suppressed trauma-induced, autophagy-mediated nerve injury in vitro. (149) | We found strong, condensed Rab11a expression in the base of hair cell kinocilia, similar as reported in primary cilia of other cell types.  (148) | membrane traffic protein  transporter | Tbio | ID-> no entry in Pharos, but through gene name  Vesicle transport | |
| 118 | CKMT1A | Creatine-kinase U-Type mitochondrial | P12532 |  | Expressed by embryonic and postnatal hair cells (27) | regulatory/  adaptor protein kinase  Transferase/ Kinase | Tbio | Candidate gene for phenotype of three families segregating an autosomal recessive contiguous gene deletion syndrome characterised by deafness and sperm dysmotility, although not an obvious phenotype (150) | |
| 119 | MMP8 | Neutrophil collagenase | P22894 | Not up or downregulated after noise exposure (140) Increased protein level in cholesteatoma (151)  MMP-8 was found in its active form in middle ear fluid (MEF) of children with otitis media with effusion (OME). New therapeutic strategies with MMP inhibitors targeting MMP-8, but allowing MMP-8 to carry out the protective action, may play a role in the future treatment of otitis media and OME. (152) | In normal cochlear sensory epithelium, more abundant in basal sample than in the apical (140) sample High expression in healthy stria vascularis (153) | protease  hydrolase | Tchem | Breakdown of extracellular matrix (Q:Pharos) | |
| 120 | SORD | Sorbitol dehydrogenase | Q00796 | Galactose rich-diet leads to diabetic-like changes through accumulation of polyols in the inner ear. SORD is rate-limitating in the polyol pathway. (154) |  | oxidoreductase | Tchem | Converts sorbitol to fructose. Part of the polyol pathway (Q:Pharos) | |
| 121 | CENPF | Centromere protein F | P49454 | Required for kinetochore function and chromosome segregation in mitosis | Enriched in cochlear and utricular hair cells retinoblastoma gene (Rb1) null mice (155) | Developmental protein | Tbio |  | |
| 123 | SORCS1 | VPS10 domain-containing receptor SorCS1 (Isoform 4) | Q8WY21-4 | One of 25 most-abundant transcripts encoding γ-secretase substrates that are present (but not necessarily differentially expressed) in the utricular sensory epithelium. (156) |  | Receptor (neuropeptide receptor activity) | Tbio |  | |
| 124 | PYGB | Glycogen phosphorylase, brain form | P11216 |  | Enriched in utricular epithelium of P5 mice, not in hair bundles (157) | transferase | Tbio | regulates glycogen mobilization | |
| 125 | THBS4 | Throbospondin-4 | P35443 | Thrombospondin 3,4,5 KO-mice do not show an auditory phenotype, only 1&2 (158)  Upregulated in rat cochleae on day after noise exposure (95)  Downregulated after gentamycin exposure in rat model (159) |  | calcium-binding protein  signaling molecule? (growth factor) | Tbio | mediates cell-to-cell and cell-to-matrix interactions, involved in various processes including cellular proliferation, migration, adhesion,… (Q:Pharos) | |
| 126 | MPST | 3-mercaptopyruvate sulfurtransferase | B1AH49 | Metabolizes homocysteine -> Hyperhomocysteinemia (HHcy) is associated with cognitive decline and hearing loss due to vascular dysfunction. (160) |  | transferase | Tchem | Contributes to the catabolism of cysteine and is an important producer of hydrogen sulfide in the brain, retina and vascular endothelial cells. Hydrogen sulfide H(2)S is an important synaptic modulator, signaling molecule, smooth muscle contractor and neuroprotectant. (Q:Pharos) | |
| 127 | PKP3 | Plakophilin 3 | Q9Y446 | Enriched in P1 Lfng-GFP^+^ supporting cells compared with P6 (70) |  | cell adhesion molecule | Tbio | May act in cellular desmosome-dependent adhesion and signaling pathways (Q:Pharos) | |
| 128 | PHGDH | D-3-phosphoglycerate dehydrogenase | Q5SZU1 | Catalyzes the reversible oxidation of 3-phospho-D-glycerate to 3-phosphonooxypyruvate, the first step of the phosphorylated L-serine biosynthesis pathway. (Q:Pharos) | Enriched gene in mice postnatal cochlear hair cells (P0) (Quelle: Positive Selection and Adaptation of Novel Inner Ear Genes in the Mammalian Lineage) | oxidoreductase | Tbio | ID-> no entry in Pharos, UniProt: obsolete, Information through protein name | |
| 129 | PSMD1 | 26S proteasome non-ATPase regulatory subunit 1 (Isoform 2) | Q99460-2 | Presents stable expression after noise exposure (50) Component of the 26s proteasome |  | enzyme modulator?? | Tbio | No isoform specific information | |
| 132 | APRT | Adenine phosphoribosyl-transferase | H3BQZ9 | SAM supplementation is proven to be an effective therapy in slowing down the progression and onset of both neurological and audiologic symptoms in patients with Arts syndrome. (associated with hearing loss). One step in SAM metabolism: Adenosine can be converted into AMP via adenine phosphoribosyl-transferase (APRT) (161) |  | transferase | Tbio | ID-> no entry in Pharos, but through gene name.  PRPP is also used for salvaging purines by adenine phosphoribosyl transferase (APRT) and hypoxanthine guanine phosphoribosyl transferase (HGPRT). Mutations in Phosporibosylpyrophosphate (PRPP) synthetase 1 (PRPS1) are associated with a spectrum of non-syndromic to syndromic hearing loss (162) | |
| 133 | HIP1R | Huntingtin-interacting protein 1-related protein | O75146 | Component of clathrin-coated pits and vesicles, that may link the endocytic machinery to the actin cytoskeleton (Q:Pharos) | Expressed in HC only, not in inner ear or sensory epithelium samples (49) | Actin-binding | Tbio |  | |
| 134 | SERBP1 | Plasminogen activator inhibitor 1 RNA-binding protein (Isoform 4) | Q8NC51-4 | Enriched 3h after combined irradiation and cisplatin treatment (not 24 or 72h, not only irradiation, not only cisplatin) (163) |  | nucleic acid binding | Tbio | Regulation of mRNA stability (Q:Pharos) | |
| 135 | RPL8 | 60S ribosomal protein L8 | E9PP36 | Highly expressed in Atoh1-induced, new, mostly immature Hair cells compared to native hair cells (164) |  | nucleic acid binding  Ribonucleoprotein | Tbio | ID-> no entry in Pharos, but through gene name. | |
| 136 | HNRNPA1 | Heterogeneous nuclear ribonucleoprotein A1 (Fragment) | H0YH80 | Might be involved in exon skipping in gene SYNE4, associated with hearing loss (165) |  | nucleic acid binding | Tchem | ID-> no entry in Pharos, but through gene name. | |
| 137 | GAA | Lysosomal alpha-glucosidase | P10253 | Essential for the degradation of glycogen in lysosomes (Q:Pharos).  Case report: Cochlear pathology is the most likely cause of hearing loss in infantile Pompe's disease and possibly a characteristic feature of this clinical subtype. (166) |  | Hydrolase  Glycosidase | Tclin | 21% of patients had a clinically relevant hearing loss (16% slight, 3% moderate, 2% profound). Though this suggests that hearing loss occurs in a considerable number of patients with Pompe disease, this prevalence is similar to that in the general population. Therefore, we conclude that hearing loss is not a specific feature of Pompe disease in adults. (167) | |
| 138 | CDH1 | Cadherin-1 | H3BNC6 | Site-specific upregulation in Cdh1 expression is spatially correlated with sensory cell degeneration. Upregulated in basal but not apical sample of the sensory epithelia after noise exposure (168) Seems to be involved in planar cell polarity (PCP) establishment, which is essential for inner ear function (169)  Cdh1 was present exclusively on P30 relative to P14, and plays a role in cell-cell adhesions, mobility and proliferation of epithelial cells (102) | In Intercellular junctions among supporting cells, including Deiters cells, pillar cells and Hensen cells, while staining in the junctions around sensory cells was weak or undetectable (168) | calcium-binding protein  cell adhesion molecule | Tbio | ID-> no entry in Pharos, but through gene name. | |
| 139 | LAMC1 | Laminin subunit gamma-1 | P11047 | Expressed in human perilymph samples, apparently upregulated by reduced expression of brain derived neurotrophic factor (BDNF). (BDNF has been shown to support spiral ganglion neurons and to improve implant function in animal models. (170) Downregulated 2h post-noise-exposure in lateral wall tissues (168) | Expressed by surrounding cells (27) | cell adhesion molecule | Tbio | Found in the basement membranes laminin is thought to mediate the attachment, migration and organization of cells (Q:Pharos) | |
| 140 | ADH5 | Alcohol dehydrogenase class-3 | P11766 | Part of the significantly enriched pathways Glycolysis/ Gluconeogenesis and fatty acid metabolism in cochlear tissues of CMP-Neu5Ac hydroxylase (Cmah)-null mice (associated with hearing loss, possibly via oxidative stress?) (171) |  | oxidoreductase | Tchem |  | |
| 142 | MYL12A | Myosin regulatory light chain 12A | P19105 | Upregulated in cochlear tissues of CMP-Neu5Ac hydroxylase (Cmah)-null mice, part of focal adhesion and platelet activation pathways. (172) |  | calcium-binding protein | Tbio |  | |
| 145 | HNRNPA3 | Heterogeneous nuclear ribonucleoprotein A3 | E7EWI9 | Plays a role in cytoplasmic trafficking of RNA. (Q:Pharos) Expressed in the chicken otocyst (The otocyst plays a pivotal role during inner ear development: otic progenitor cells sub-compartmentalize into non-sensory and prosensory domains, giving rise to individual vestibular and auditory organs and their associated ganglia.) (173)  Detected in human middle-ear epithelial cell (HMEEC) exosomes. (174) |  | nucleic acid binding | Tbio | ID-> no entry in Pharos, but through gene name. | |
| 146 | NBN | Nibrin | O60934 | Case report: Boy with Nijmegen breakage syndrome showed severe dysplasia of the cochlea and severe hearing impairment (without NBS1 mutation) |  | nucleic acid binding (damaged DNA-binding)  Cell cycle | Tbio | DNA repair | |
| 148 | RAB21 | Ras-related protein Rab-21 | Q9UL25 | Colocalizes with Vangl2 (Van gogh-like 2), which is essential in planar cell polarity (PCP) pathway in the inner ear. (175) |  | Transporter (Protein transport) | Tbio | Regulates integrin internalization and recycling (Q:Pharos) | |
| 149 | HPRT1 | Hypoxanthine-guanine phosphoribosyl-transferase | P00492 | Highly expressed in normal and noise damaged cochlear sensory epithelia (140) |  | transferase | Tchem | Often used as a stable control or reference gene (176) | |
| 155 | PRDX5 | Peroxiredoxin-5, mitochondrial (Isoform Cytoplasmic+peroxisomal) | P30044-2 | Higly expressed in young rat cochlea (2 month) (177)  Expression changes 72h after combined treatment of irradiation and cisplatin. (163) |  | oxidoreductase | Tbio | No isoform specific information  Cell protection against oxidative stress (Q:Pharos) | |
| 156 | ACAT2 | Acetyl-CoA acetyltransferase, cytosolic | Q9BWD1 | Downregulated in cochlear tissues of CMP-Neu5Ac hydroxylase (Cmah) null mice compared with wild type in different pathways, e.g. Carbon metabolism (172) |  | Transferase | Tchem | Lipid metabolism, utilization of ketone bodies (Q:Pharos/OMIM) | |
| 157 | YME1L1 | ATP-dependent zinc metalloprotease YME1L1 (Isoform 3) | Q96TA2-3 | Highly enriched in Hair cells (vestibular) vs. supporting cells. (Several genes highly expressed in hair cells are associated with various neurologic diseases (Spg7, Yme1l1 and Elovl4) and consequently may play a role in vestibular dysfunction.) (178) Case report: 4 affected children with a homozygous mutation in the YME1L1 gene develop an infantile-onset mitochondriopathy with hearing loss (179) |  | protease  hydrolase | Tbio | No isoform specific information  ATP-dependent Metalloprotease (Q:Pharos) | |
| 158 | ANXA4 | Annexin A4 | B4DDF9 | ANXA4 is a specific marker for type II utricular hair cells (180)  Upregulated in Retinoblastoma gene (Rb1) knockout mice in cochlear and untricular hair cells (181)  completely downregulated in hair cells 24 h after deletion of Atoh1. (138)  Myc also regulates Anxa4, which is upregulated in Micro-RNA mir-96 mutated mice, which present deafness (182) | Expressed by embryonic and postnatal hair cells (27)  Expressed in cochlear hair cells (183)  In cochlear hair cells expressed more strongly in the base than the apex (138) | calcium-binding protein | Tbio | Calcium/phospholipid-binding protein which promotes membrane fusion and is involved in exocytosis (Q:Pharos) | |
| 159 | IFITM2 | Interferon-induced transmembrane protein 2 | Q01629 | IFN-induced antiviral protein which inhibits the entry of viruses to the host cell cytoplasm (Q:Pharos) | Expressed in surrounding cells (27) Highly expressed immune/ inflammation-related gene in the normal cochlear sensory epithelium (85) | defense/immunity protein | Tbio |  | |
| 160 | NID1 | Nidogen-1 (Isoform2) | P14543-2 | Exhibits large fold changes in expression in only the saccules compared with cochleae and utricles, possibly useful al saccule signature gene (36) | Expressed by surrounding cells (27) | cell adhesion molecule | Tbio |  | |
| 163 | RAB10 | Ras-related protein Rab-10 | P61026 | Putative interaction partner of prestin (Q:Dissertation) | Identified in Cochlear membranes of rat and mouse, otoferlin interaction partner (184) | hydrolase | Tbio | Small GTPase, mainly involved in transport of proteins (Q:Pharos) | |
| 165 | AKR1A1 | Alcohol dehydrogenase [NADP(+)] | P14550 |  | Highly enriched non-sensory cell protein. (30) | oxidoreductase | Tchem |  | |
| 166 | JAK1 | Janus Kinase 1 | P23458 | STAT3 signaling-related gene (important role of the STAT3 signaling during mouse cochlear hair cell differentiation) (185) JAK1/STAT3 pathway was active within 1h after HC loss (186,187), during zebrafish inner ear regeneration: Possibly, Jak1/Stat3 signaling regulates proliferation in support cells in the first few hours after hair cell death, whereas Wnt/β-catenin signaling is required for proliferation during later stages of regeneration. cell cycle is activated immediately, possibly via cytokine stimulation of the Jak1/Stat3 pathway. (186)  JAK1 interacts with LMO4 and is downregulated by cisplatin treatment (107)  Knockdown of Interferon lambda receptor 1 (IFNLR1) in zebrafish causes hearing loss and upregulation in of JAK1 (u.a.) (188) | Little expression in hair cells, non-hair cells epithelia and mesenchymal cells (185) | regulatory/ adaptor protein kinase | Tclin |  | |
| 167 | ALDH2 | Aldehyde dehydrogenase, mitochondrial | F8W0A9 |  | Expressed by surrounding cells (27) | oxidoreductase | Tclin | ID-> no entry in Pharos, UniProt: obsolete, Information through protein name | |
| 168 | GYPC | Glycophorin-C (Isoform Glycophorin-D) | P04921-2 |  | Expressed in surrounding cells (27)  Expressed in mouse early postnatal auditory and vestibular sensory epithelia (189) | defense/immunity protein?? (Blood group antigen) | Tbio |  | |
| 170 | PTPRS | Receptor-type tyrosine-protein phosphatase S (Isoform 2) | Q13332-6 | Primary axonogenesis, and axon guidance during embryogenesis. This PTP has been also implicated in the molecular control of adult nerve repair. (Q:Pharos) | Expressed by surrounding cells (27) | hydrolase  receptor  phosphatase | Tchem | No isoform specific information | |
| 171 | PSMB9 | Proteasome subunit beta type-9 (Fragment) | B0V0T2 | Upregulated in rat cochlear tissue one day after noise exposure. (95)  proteasomopathies that include PSMB9 (U.a.) linked to loss of function mutations in inner ear, lens and central nervous system development. (190) |  | protease  hydrolase | Tchem | ID-> no entry in Pharos, UniProt: obsolete, Information through protein name.  Proteasome | |
| 172 | DNPH1 | 2'-deoxynucleoside 5'-phosphate N-hydrolase 1 (Isoform 2) | O43598-2 | Independent single-nucleotide polymorphism (SNP) in this gene are associated with the two phenotypes (Age related hearing loss: subjective hearing difficulty vs hearing aid use) regarding hearing ability in the UK Biobank Discovery Sample (125) |  | Hydrolase, glycosidase | Tchem | No isoform specific information | |
| 173 | NAMPT | Nicotinamide phosphoribosyltransferase | P43490 | In this study, we report that NAD+ levels are reduced in the cochlea of CSBm/m mice (Cockayne syndrome) and that short-term treatment (10 days) with the NAD+ precursor nicotinamide riboside (NR), prevents hearing loss, restores outer hair cell loss, and improuchl1  ves cochlear health in CSBm/m mice. Similar, but more modest effects were observed in CSA−/− mice.  (191)  We find that administration of Nicotamide Riboside, an NAD+ precursor, even after noise exposure, prevents [noise-induced hearing loss](https://www.sciencedirect.com/topics/medicine-and-dentistry/noise-induced-hearing-loss) (NIHL) and spiral ganglia neurite degeneration. (192)  Neuroprotective Nampt Inhibitor P7C3 Demonstrates Otoprotection in an Age-Related Hearing Loss Model and may represent a novel therapeutic strategy for presbycusis. (Q: Conference Paper) |  | signaling molecule (cytokine)  **transferase** | Tchem |  | |
| 174 | CDC42 | Cell division control protein 42 homolog | P60953 | Cdc42 influenced the maintenance of stable actin structures through elaborate tuning of actin turnover, and maintained function and viability of cochlear hair cells. (193)  Hair cells of Atoh1–Cre;Cdc42flox/flox mice developed normally but progressively degenerated after maturation, resulting in progressive hearing loss particularly at high frequencies (193)  After normal morphological maturation, the Cdc42-KO mice showed progressive sensorineural hearing loss (SNHL), particularly at high frequencies, and HC loss accompanied by various stereociliary abnormalities starting at postnatal day 14 (P14) (scattered, short, long, and fused) predominantly at the IHCs of the basal turn. (194)  CDC42 is part of hair cell polarity establishment and required for stereociliogenensis (195) Levels of active CDC42 and RAC1 were dramatically decreased in the Arhgef6 knockdown mice, suggesting that ARHGEF6 regulates stereocilia maintenance through RAC1/CDC42. (196) | At E18.5: Cdc42 was localized to the OHC apex. Expression was found in the region around the basal body from where microtubules radiate at the cell surface. Weaker expression was found in the apical cytoplasm, on the medial side of the bundle. In addition, stereocilia showed strong expression as well as the contact sites between supporting cells and OHCs at the level of adherens junctions. Cdc42 was also expressed in the apices of IHCs and supporting cells. (195) | hydrolase | Tchem | Takenouchi-kosaki syndrome (some patients present sensorineural hearing loss) (Q:Pharos/OMIM) | |
| 175 | MTPN | Myotrophin | C9JL85 | Expression changed 3h after combined irradiation and cisplatin exposure (163) | Expressed in OHCs (39)  One of the 10 most abundantly expressed transcription factor in OHCs and IHCs (197)  Identified in mouse or chick hair-bundle proteome data (198) | transcription factor?? | Tbio | ID-> no entry in Pharos, but through gene name. | |
| 176 | ANPEP | Aminopeptidase N | P15144 | Broad specificity aminopeptidase which plays a role in the final digestion of peptides (Q:Pharos) | Expressed by surrounding cells (27) | Hydrolase protease receptor | Tchem |  | |
| 177 | COL6A3 | Collagen alpha-3(VI) chain (Isoform 3) | P12111-3 |  | Expressed in otosclerotic bone, but not in normal otic capsule (199)  Expressed along the tonotopic axis of the mouse cochlea, downregulated at P8 compared with P0 (4) | cell adhesion molecule | Tbio | No isoform specific information | |
| 178 | RAB1B | Ras-related protein Rab-1B | E9PLD0 | RAB1B regulates vesicular transport between the endoplasmic reticulum and successive Golgi compartments. (Q:Pharos) | Detected in hair-bundle proteomes of wild type mice, but not in Anxa5-/--mice (200) | transporter | Tbio | ID-> no entry in Pharos, but through gene name. | |
| 180 | GRHPR | Glyoxylate reductase/hydroxypyruvate reductase | Q9UBQ7 | Enzyme with hydroxy-pyruvate reductase, glyoxylate reductase and D-glycerate dehydrogenase enzymatic activities | Identified in hair cell sample, but not in inner ear or sensory epithelia datasets (less sample complexity in MS) (49) | oxidoreductase | Tbio |  | |
| 181 | HMGCS1 | Hydroxymethylglutaryl-CoA synthase, cytoplasmic | Q01581 | Osbpl2/osbpl2b-KO associated with autosomal dominant nonsyndromic hearing loss, Hmgcs1/hmgcs1 upregulated (201) | IHC transcription factor (202) | transferase  transcription factor? | Tchem | This enzyme condenses acetyl-CoA with acetoacetyl-CoA to form HMG-CoA, which is the substrate for HMG-CoA reductase. (Q:Pharos) | |
| 182 | RPS9 | 40S ribosomal protein S9 | P46781 | Highly expressed in Atoh1-induced “new” hair cells, but not in native hair cells (164) | Expressed in an organ of corti cell line (203) | nucleic acid binding (ribonucleo-protein) | Tbio |  | |
| 183 | PSMB3 | Proteasome subunit beta type-3 | P49720 | Expressed in Atoh1-induced “new” hair cells, but not in native HCs (164)  Differentially expressed in cmah-null-mice (171) |  | Protease hydrolase | Tdark | Component of the 20S core proteasome complex (Q:Pharos) | |
| 184 | ELOVL4 | Elongation of very long chain fatty acids protein 4 | Q9GZR5 | Catalyzes the first and rate-limiting reaction of the four that constitute the long-chain fatty acids elongation cycle. (Q:Pharos) | Expressed by postnatal hair cells (27) | Transferase | Tbio |  | |
| 185 | TGFBR2 | TGF-beta receptor type-2 | TGFBR2 | Tgfb3 and its receptor Tgfbr2, which are associated with scar-less wound healing, were down-regulated in electrode analog insertion trauma (EIT, e.g. following cochlea implantation in cochlear tissues, representing a possible therapeutic target for prevention of fibrotic scar in future studies. (204)  Decreased expression in cochlear samples after noise exposure (205) | High level of expression in the rat cochlear regions, also reported in the human fetal cochlear cDNA library (206)  Expressed by surrounding cells (27) | transferase  receptor  regulatory/adaptor protein kinase?? | Tchem |  | |
| 186 | NDRG1 | Protein NDRG1 | E5RJY1 | Upregulated 2 days after noise exposure and growth factor injection in zebrafish inner ear (Q: Thesis “Next Generation Sequencing Reveals Gene Expression Patterns in the Zebrafish Inner Ear Following Growth Hormone Injection”) | Expressed by surrounding cells (27)  Enriched in spiral ganglion at E12 compared with vestibular ganglion. (103) | signaling molecule | Tbio | ID-> no entry in Pharos, but through gene name. | |
| 188 | RNASE4 | Ribonuclease 4 | P34096 |  | Expressed by surrounding cells (27) | Hydrolase (nuclease) | Tbio | RNAse | |
| 189 | ITGAM | Integrin alpha-M | P11215 | Upregulated one day after noise exposure (95)  Increased expression after cochlear injury, molecules known as hematopoietic and macrophage/microglial cell makers were also found to increase in the injured ANs. (207) | Expressed in normal rat cochlea (168)  Weakly expressed immune/inflammation-related gene in normal cochlear sensory epithelia (85) | receptor  cell adhesion molecule | Tbio | Integrin ITGAM/ITGB2 is implicated in various adhesive interactions of monocytes, macrophages and granulocytes as well as in mediating the uptake of complement-coated particles. It is identical with CR-3, the receptor for the iC3b fragment of the third complement component. (Q:Pharos) | |
| 190 | PSMC6 | 26S protease regulatory subunit 10B (Fragment) | H0YJS8 | Stable gene (no expression changes) after acoustic overstimulation in proteasome pathway (50) |  | enzyme modulator | Tbio | ID-> no entry in Pharos, but through gene name. | |
| 191 | GJA4 | Gap junction alpha-4 protein | Q5JW71 | Upregulated one day after noise exposure (95) |  | cell junction protein | Tbio | ID-> no entry in Pharos, but through gene name. | |
| 192 | COPB2 | Coatomer subunit beta' | B4DZI8 | The coatomer is a cytosolic protein complex that binds to dilysine motifs and reversibly associates with Golgi non-clathrin-coated vesicles. (Q:Pharos) | Expressed in an organ of corti cell line (203)  Expressed in utricular epithelium at P28 (157) | transporter | Tbio | No isoform specific information | |
| 195 | IL1RN | Interleukin-1 receptor antagonist protein (Isoform 4) | P18510-4 | IL-1 blockade with anakinra in patients with NOMID can reverse organ inflammation imaged on magnetic resonance imaging, including CNS leptomeningitis and cochlear inflammation (208) keine unmittelbare Bedeutung für IL1RN, da dieses zwar bei der Krankheit DIRA mutiert ist, dies aber keine ZNS Beteiligung aufweist. Nur interessant, weil IL1RN ja auch die Aktivität von IL-1 beeinflusst.  Proposed gene to be involved in pathogenesis of Otitis media, significantly associated (209) | Detected in cochlear sensory epithelia, but not in organ of Corti cell samples of mice. (85) | enzyme modulator  signaling molecule (cytokine activity) | Tchem, but 1 approved drug | No isoform specific information  Inhibits the activity of interleukin-1 by binding to receptor IL1R1 and preventing its association with the coreceptor IL1RAP for signaling. (Q:Pharos) | |
| 196 | PTMS | Parathymosin | P20962 |  | Expressed in an organ of corti cell line (203) | defense/immunity protein | Tbio |  | |
| 197 | NANS | Sialic acid synthase | Q9NR45 |  | Exclusively indentified in non-sensory cells compared with vestibular hair cells of chicken embryos (30) | Transferase | Tbio | in the biosynthetic pathways of sialic acids (Q:Pharos) | |
| 199 | PDCD6IP | Programmed cell death 6-interacting protein | Q8WUM4 | Upregulated in cochleae with mtDNA mutations (210) | Expressed in E15 chicken utricles (211)  Expressed in small extracellular vesicles from auditory cells (212) | Transporter  Cell cycle protein | Tbio | By interacting with F-actin, PARD3 and TJP1 secures the proper assembly and positioning of actomyosin-tight junction complex at the apical sides of adjacent epithelial cells that defines a spatial membrane domain essential for the maintenance of epithelial cell polarity and barrier. Protection against cell death. (Q:Pharos) | |
| 201 | ARPC3 | Actin-related protein 2/3 complex subunit 3 (Fragment) | C9JZD1 |  | Expressed in an organ of Corti cell line (203) | cytoskeletal protein | Tbio | ID-> no entry in Pharos, but through gene name.  Component of the Arp2/3 complex, a multiprotein complex that mediates actin polymerization upon stimulation by nucleation-promoting factor (NPF) (Q:Pharos) | |
| 205 | CMPK1 | UMP-CMP kinase | E9PGI8 | Catalyzes the phosphorylation of pyrimidine nucleoside monophosphates at the expense of ATP. Plays an important role in de novo pyrimidine nucleotide biosynthesis. (Q:Pharos) | Expressed in an organ of Corti cell line (203) | Transferase/ kinase  regulatory/adaptor protein kinase | Tbio |  | |

**Druggability:**

Tclin:

| # | Gene | Protein | ID | Information about Cochlea? | Protein Group | Further information/ Literature |
| --- | --- | --- | --- | --- | --- | --- |
| 51 | CA3 | Carbonic anhydrase 3 | P07451 | Yes | lyase | 5 approved drugs: acetazolamide, ...  11 active ligands |
| 63 | TYMP | Thymidine phosphorylase | C9JGI3 | Yes | transferase  signaling molecule | 1 approved drug: Tipiracil,  21 active ligands |
| 65 | GBA | Glucosylceramidase | J3KQG4 | Yes | Hydrolase  transferase | 1 approved drug: Ambroxol,  169 active ligands |
| 101 | IDH1 | Isocitrate dehydrogenase [NADP] cytoplasmic | O75874 | Yes | oxidoreductase | 1 approved drug: Ivosidenib  706 active ligands |
| 108 | ACLY | ATP-citrate synthase | B4E3P0 | Yes | transferase | 1 approved drug: bempedoic acid  18 active ligands |
| 137 | GAA | Lysosomal alpha-glucosidase | P10253 | Yes | Hydrolase  Glycosidase | 4 approved drugs: miglustat, miglitol, migalastat, voglibose  9 active ligands |
| 147 | ADH1B | Alcohol dehydrogenase 1B | P00325 | No | oxidoreductase | Alcohol metabolism  1 approved drug: fomepizole  2 active ligands |
| 166 | JAK1 | Janus Kinase 1 | P23458 | Yes | regulatory/ adaptor protein kinase | 7 approved drugs: sunitinib, nintedanib, tofacitinib, ruxolitinib, fedratinib, baricitinib, upadacitinib  2671 active ligands |
| 167 | ALDH2 | Aldehyde dehydrogenase, mitochondrial | F8W0A9 | Yes | oxidoreductase | 1 approved drug: disulfiram  82 active ligands |

Tchem:

| # | Gene | Protein | ID | Information about Cochlea? | Protein Group | Further Information/ Literature |
| --- | --- | --- | --- | --- | --- | --- |
| 15 | DNASE1 | Deoxyribonuclease 1 | P24855 | Yes, aber wenig und ohne gut gesicherte Quellen | Hydrolase | 1 active ligand |
| 19 | PRSS8 | Prostasin | Q16651 | Yes | Protease | 16 active ligands |
| 23 | GLUL | Glutamine synthetase | P15104 | Yes | Ligase/ transferase | 2 approved drugs: prednisolone, prednisolone tebutate  8 active ligands |
| 31 | TGM2 | Protein-glutamine gamma-glutamyltransferase 2 | B4DIT7  P21980 | Yes | transferase | 181 active ligands |
| 32 | MDH2 | Malate dehydrogenase | G3XAL0 | Yes | oxidoreductase | 1 active ligand |
| 46 | PTPRF | Receptor-type tyrosine-protein phosphatase F (Isoform 2) | P10586-2 | no | cell junction protein, transmembrane receptor, phosphatase | 10 active ligands  No isoformsprecific information. |
| 72 | PTPA | Serine/threonine-protein phosphatase 2A activator (Isoform 3) | Q15257-3 | No, but noise induced increase of serine threonine protein phosphatase 2A/B in modiolus (213) (Protein is activated by PTPA) | enzyme modulator | 1 active ligand  Information not isoform specific |
| 76 | GSTM2 | Glutathione S-transferase Mu 2 | E9PHN7 | Yes | transferase | 34 active ligands |
| 94 | IDE | Insulin-degrading enzyme | P14735 | No | Protease/ hydrolase/  receptor | 31 active ligands |
| 99 | CYBB | Cytochrome b-245 heavy chain  Alternative name:  NADPH OXIDASE 2; NOX2 | F5GWD2 | yes | membrane traffic protein (ion channel)  oxidoreductase | 9 active ligands |
| 109 | ARF1 | ADP ribosylation factor 1 | P84077 | Yes | Transporter | 1 active ligand |
| 110 | UCHL1 | Ubiquitin carboxyl-terminal hydrolase isozyme L1 | D6RE83 | Yes | protease  hydrolase | 6 active ligands |
| 116 | ASAH1 | Acid ceramidase | E7ERV9 | Yes | hydrolase | 46 active ligands |
| 119 | MMP8 | Neutrophil collagenase | P22894 | Yes | protease  hydrolase | 891 active ligands |
| 120 | SORD | Sorbitol dehydrogenase | Q00796 | Yes | oxidoreductase | 64 active ligands |
| 126 | MPST | 3-mercaptopyruvate sulfurtransferase | B1AH49 | Yes | transferase | 1 active ligand |
| 136 | HNRNPA1 | Heterogeneous nuclear ribonucleoprotein A1 (Fragment) | H0YH80 | Yes | nucleic acid binding | 1 active ligand |
| 140 | ADH5 | Alcohol dehydrogenase class-3 | P11766 | Yes | oxidoreductase |  |
| 149 | HPRT1 | Hypoxanthine-guanine phosphoribosyl-transferase | P00492 | Yes | transferase | 45 active ligands |
| 153 | CTSV | Cathepsin L2 | O60911 | No | protease  hydrolase | 1 approved drug: Boceprevir  62 active ligands |
| 156 | ACAT2 | Acetyl-CoA acetyltransferase, cytosolic | Q9BWD1 | Yes | Transferase | 1 active ligand |
| 165 | AKR1A1 | Alcohol dehydrogenase [NADP(+)] | P14550 | Yes | oxidoreductase | 1 active ligand |
| 170 | PTPRS | Receptor-type tyrosine-protein phosphatase S (Isoform 2) | Q13332-6 | Yes | hydrolase  receptor  phosphatase | 8 active ligands |
| 171 | PSMB9 | Proteasome subunit beta type-9 (Fragment) | B0V0T2 | Yes | protease  hydrolase | 6 active ligands |
| 172 | DNPH1 | 2'-deoxynucleoside 5'-phosphate N-hydrolase 1 (Isoform 2) | O43598-2 | Yes | Hydrolase, glycosidase | 2 active ligands |
| 173 | NAMPT | Nicotinamide phosphoribosyltransferase | P43490 | Yes | signaling molecule (cytokine)  **transferase** | 2067 active ligands |
| 174 | CDC42 | Cell division control protein 42 homolog | P60953 | Yes | hydrolase | 1 active ligand |
| 176 | ANPEP | Aminopeptidase N | P15144 | Yes | Hydrolase protease receptor | 86 active ligands |
| 181 | HMGCS1 | Hydroxymethylglutaryl-CoA synthase, cytoplasmic | Q01581 | Yes | transferase  transcription factor? | 1 active ligand |
| 185 | TGFBR2 | TGF-beta receptor type-2 | TGFBR2 | Yes | transferase  receptor  regulatory/adaptor protein kinase?? | 7 active ligands |
| 195 | IL1RN | Interleukin-1 receptor antagonist protein (Isoform 4) | P18510-4 | Yes | enzyme modulator  signaling molecule (cytokine activity) | 0 active ligands, 1 approved drug: rilonacept |

**Protein groups:**

cell adhesion molecule

| # | Gene | Protein | ID | Information about Cochlea | Druggability | Further information/ Literature |
| --- | --- | --- | --- | --- | --- | --- |
| 36 | MSLN | Mesothelin (Fragment) | H3BUX1 | Yes, but about homology | Tbio |  |
| 53 | THY1 | Thy-1 membrane glycoprotein | J3QRJ3 | No | Tbio | ID-> no entry in Pharos, through gene nam  cell surface glycoprotein and member of the immunoglobulin superfamily of proteins (Q:Pharos) defense/immunity protein  cell adhesion molecule |
| 80 | LAMB1 | Laminin subunit ß-1 | P07942 | Yes | Tbio |  |
| 96 | CDH11 | Cadherin 11 (Isoform 2) | P55287-2 | Yes | Tbio | calcium-binding protein  cell adhesion molecule |
| 97 | NID2 | Nidogen-2 (Isoform 2) | Q14112-2 | Yes | Tbio |  |
| 127 | PKP3 | Plakophilin 3 | Q9Y446 | Yes | Tbio |  |
| 138 | CDH1 | Cadherin-1 | H3BNC6 | Yes | Tbio | calcium-binding protein  cell adhesion molecule |
| 139 | LAMC1 | Laminin subunit gamma-1 | P11047 | Yes | Tbio |  |
| 160 | NID1 | Nidogen-1 (Isoform2) | P14543-2 | Yes | Tbio |  |
| 177 | COL6A3 | Collagen alpha-3(VI) chain (Isoform 3) | P12111-3 | Yes | Tbio |  |
| 189 | ITGAM | Integrin alpha-M | P11215 | Yes | Tbio |  |

cell junction protein

| # | Gene | Protein | ID | Information about Cochlea | Druggability | Further information/ Literature |
| --- | --- | --- | --- | --- | --- | --- |
| 191 | GJA4 | Gap junction alpha-4 protein | Q5JW71 | Yes | Tbio |  |
| 193 | DSG2 | Desmoglein-2 | Q14126 | No | Tbio | Component of intercellular desmosome junctions cell adhesion molecule  calcium-binding protein |

cytoskeletal protein

| # | Gene | Protein | ID | Information about Cochlea | Druggability | Further information/ Literature |
| --- | --- | --- | --- | --- | --- | --- |
| 6 | KRT87P | Putative keratin 87 protein | A6NCN2 | no | Tdark | Mögliche Kontamination? |
| 8 | KRT33A | Keratin 33A | O76009 | no | Tbio |  |
| 10 | KRT82 | Keratin 82 | Q9NSB4 | no | Tdark |  |
| 12 | MYH9 | Myosin 9 | P35579 | yes | Tbio |  |
| 14 | IVL | Involucrin | P07476 | Yes, but only about middle ear | Tbio |  |
| 21 | PPL | Periplakin | K7EKI8 | no | Tbio | ID-> no entry, only with Proteinname,  Might have a role in Protein signaling |
| 25 | EVPL | Envoplakin | Q92817 | no | Tbio | Cornified envelope of ceratinozytes |
| 41 | CAP1 | Adenylyl cyclase-associated protein 1 | Q01518 | no | Tbio | Directly regulates filament dynamics, establishment of cell polarity (Q:Pharos) |
| 44 | CEACAM16 | Carcinoembryonic antigen-related cell adhesion molecule 16 | Q2WEN9 | yes | Tbio |  |
| 57 | ARPC4 | Actin-related protein 2/3 complex subunit 4 | F8WDD7 | yes | Tbio |  |
| 58 | TPM3 | Tropomyosin alpha-3 chain | Q5VU66 | Yes | Tbio |  |
| 105 | TPM1 | Tropomyosin alpha-1 chain | D9YZV8 | No | Tbio | Actin-binding |
| 111 | ACTR3 | Actin-related protein 3 | F5H3P5 | Yes | Tbio |  |
| 201 | ARPC3 | Actin-related protein 2/3 complex subunit 3 (Fragment) | C9JZD1 | Yes | Tbio |  |

enzyme modulator

| # | Gene | Protein | ID | Information about Cochlea | Druggability | Further information/ Literature |
| --- | --- | --- | --- | --- | --- | --- |
| 5 | RARRES1 | Retinoic acid receptor responder 1 | P49788 | yes | Tbio |  |
| 30 | SUMO3 | Small ubiquitin-related modifier 3 | P55854 | yes | Tbio |  |
| 39 | LXN | Latexin | Q9BS40 | yes | Tbio |  |
| 55 | SPINT1 | Kunitz-type protease inhibitor 1 (Fragment) | H3BTQ8 | yes | Tbio |  |
| 67 | SERPINB2 | Plasminogen activator inhibitor 2 | P05120 | yes | Tbio |  |
| 72 | PTPA | Serine/threonine-protein phosphatase 2A activator (Isoform 3) | Q15257-3 | No, but noise induced increase of serine threonine protein phosphatase 2A/B in modiolus (213) (Protein is activated by PTPA) | Tchem | Information is not isoform specific |
| 81 | SERPINB8 | Serpin B8 (Fragment) | H7BXK7 | yes | Tbio | enzyme modulator (Serin protease inhibitor)  Cell adhesion |
| 82 | PEBP4 | Phosphatidyl-ethanolamine-binding protein 4 | Q96S96 | yes | Tbio | enzyme modulator (Serin protease inhibitor) |
| 84 | CAND1 | Cullin-associated NEDD8-dissociated protein 1 | Q86VP6 | yes | Tbio |  |
| 93 | CSTB | Cytstatin B | P04080 | no | Tbio | Intracellular thiol protease inhibitor |
| 104 | MED14 | Mediator of RNA polymerase II transcription subunit 14 (Fragment) | H7C017 | no | Tbio | ID-> no entry in Pharos, but through gene name  Ubiquitous. (Q:UniProt) Coactivator involved in the regulated transcription of nearly all RNA polymerase II-dependent genes. (Q:Pharos) |
| 112 | TIMP1 | Metalloproteinase inhibitor 1 | Q5H9A7 | Yes | Tbio | signaling molecule  enzyme modulator (Inhibitor) |
| 129 | PSMD1 | 26S proteasome non-ATPase regulatory subunit 1 (Isoform 2) | Q99460-2 | Yes | Tbio | Proteasome |
| 161 | FBXO07 | F-box only protein 7 (Isoform 3) | Q9Y3I1-3 | No | Tbio | No isoform specific information  Ubiquitination. |
| 179 | PSMF1 | Proteasome inhibitor PI31 subunit (Fragment) | Q5QPM9 | No | Tbio | ID-> no entry in Pharos, but through gene name  Inhibitor of proteasome |
| 190 | PSMC6 | 26S protease regulatory subunit 10B (Fragment) | H0YJS8 | Yes | Tbio |  |
| 195 | IL1RN | Interleukin-1 receptor antagonist protein (Isoform 4) | P18510-4 | Yes | Tchem | enzyme modulator  signaling molecule (cytokine activity) |
| 206 | PSMD3 | 26S proteasome non-ATPase regulatory subunit 3 | O43242 | No | Tbio | Component of the 26S proteasome |

membrane traffic protein

| # | Gene | Protein | ID | Information about Cochlea | Druggability | Further information/ Literature |
| --- | --- | --- | --- | --- | --- | --- |
| 37 | LMAN2 | Vesicular integral-membrane protein VIP36 (Fragment) | D6RIU4 | no | Tbio | ID-> no entry in Pharos, but through gene name  Ubiquitous. (Q:UniProt)  Protein transport of high mannose-type glycans between golgi, ER & PM |
| 43 | SDCBP | Synthenin (Isoform3) | O00560-3 | yes | Tbio | signaling molecule/ **membrane traffic protein**/ cell junction protein?? |
| 52 | ITSN1 | Intersectin-1 (Isoform 4) | Q15811-4 | no | Tbio | Clathrin coated vesicle transport and endocytosis, actin assembly |
| 68 | SLC9C1 | Sodium/hydrogen exchanger 10 (Isoform 2) | SLC9C1 | no | Tdark | Intracellular pH regulation of spermatozoa |
| 70 | BCAP31 | B-cell receptor-associated protein 31 | P51572 | yes | Tbio | enzyme modulator |
| 79 | PLP2 | Proteolipid protein 2 | Q04941 | yes | Tbio |  |
| 83 | PLP1 | Myelin proteolipid protein | P60201 | yes | Tbio |  |
| 99 | CYBB | Cytochrome b-245 heavy chain  Alternative name:  NADPH OXIDASE 2; NOX2 | F5GWD2 | yes | Tchem | membrane traffic protein (ion channel)  oxidoreductase |
| 117 | RAB11A | Ras-related protein Rab-11A (Fragment) | H3BMH2 | Yes | Tbio | Transporter? |

defense/immunity protein

| # | Gene | Protein | ID | Information about Cochlea | Druggability | Further information/ Literature |
| --- | --- | --- | --- | --- | --- | --- |
| 20 | BPIFB2 | BPI fold-containing family B member 2 | Q8N4F0 | no | Tdark | Almost no information at all, might bind bacterial products |
| 38 | TRIM29 | Tripartite motif-containing protein 29 (Isoform Beta) | Q14134-2 | Yes | Tbio | **defense/immunity protein**  nucleic acid binding/ transcription factor? |
| 48 | BPIFB4 | BPI fold-containing family B member 4 (Isoform 2) | P59827-2 | No | Tbio |  |
| 61 | IGHV3-30 | Immunoglobulin heavy variable 3-30 | P01769 | No | Tdark | Component of heavy chain |
| 69 | TLR3 | Toll-like receptor 3 | E9PGH4 | Yes | Tbio | receptor  nucleic acid binding |
| 73 | TESPA1 | Protein TESPA1 (Fragment) | H0YEX3 | no | Tbio | ID-> no entry in Pharos, but through gene name  Essential for Tcell maturation  defense/immunity protein  signaling molecule?? membrane traffic protein?? |
| 92 | HMGB1 | High mobility group protein B1 | Q5T7C4 | Yes | Tbio | defense/immunity protein  signaling molecule  nucleic acid binding |
| 144 | CD207 | C-type lectin domain family 4 member K | Q9UJ71 | No | Tbio | Involved in immune response, especially in Langerhans cells |
| 159 | IFITM2 | Interferon-induced transmembrane protein 2 | Q01629 | Yes | Tbio |  |
| 168 | GYPC | Glycophorin-C (Isoform Glycophorin-D) | P04921-2 | Yes | Tbio | Blood group antigen |
| 196 | PTMS | Parathymosin | P20962 | Yes | Tbio |  |

extracellular matrix protein

| # | Gene | Protein | ID | Information about Cochlea | Druggability | Further information/ Literature |
| --- | --- | --- | --- | --- | --- | --- |
| 13 | MFAP4 | Microfibril-associated glycoprotein 4 | P55083 | yes | Tbio |  |
| 40 | MUC5B | Mucin-5B | Q9HC84 | Yes, but about middle ear | Tbio |  |
| 45 | COL2A1 | Collagen alpha 1 (II) chain (Isoform1) | P02458-1 | Yes | Tbio |  |
| 49 | COL11A2 | Collagen alpha-2 (XI) chain | Q6ZNN4 | Yes | Tbio |  |
| 64 | SNED1 | Sushi, nidogen and EGF-like domain-containing protein 1 (Isoform 4) | Q8TER0-5 | No, but has a nido and EGF domain, same as alpha tectorin (TM component) (214) | Tbio | No isoform specific information  Putative metastasis factor |
| 103 | PRELP | Prolargin | P51888 | No | Tbio | Anchoring basement membrane |

surfactant

| # | Gene | Protein | ID | Information about Cochlea | Druggability | Further information/ Literature |
| --- | --- | --- | --- | --- | --- | --- |

receptor

| # | Gene | Protein | ID | Information about Cochlea | Druggability | Further information/ Literature |
| --- | --- | --- | --- | --- | --- | --- |
| 123 | SORCS1 | VPS10 domain-containing receptor SorCS1 (Isoform 4) | Q8WY21-4 | Yes | Tbio | Receptor (neuropeptide receptor activity) |
| 170 | PTPRS | Receptor-type tyrosine-protein phosphatase S (Isoform 2) | Q13332-6 | Yes | Tchem | Hydrolase receptor  phosphatase |
| 185 | TGFBR2 | TGF-beta receptor type-2 | TGFBR2 | Yes | Tchem | transferase  receptor  regulatory/adaptor protein kinase?? |
| 194 | VSIG4 | V-set and immunoglobulin domain-containing protein 4 (Isoform 2) | Q9Y279-2 | No | Tbio | receptor  defense/immunity protein: Phagocytic receptor, strong negative regulator of T-cell proliferation and IL2 production. (Q:Pharos) No isoform specific information |
| 203 | ZP2 | Zona pellucida sperm-binding protein 2 | Q4VAP1 | No, but contains ZP-domains, and Alpha and beta tectorins (components of TM) also contain zona pellucida domains (215) | Tbio | Sperm specific? |

transporter

| # | Gene | Protein | ID | Information about Cochlea | Druggability | Further information/ Literature |
| --- | --- | --- | --- | --- | --- | --- |
| 15 | SCGB2A1 | Secretoglobin family 2a member 1 | O75556 | no | Tbio | Could be receptor to, is secreted into body fluids (lung, lacrimal and salivary gland, protate, uterus, …) |
| 16 | LCN15 | Lipocalin-15 | Q6UWW0 | no | Tdark | Protein group?? |
| 26 | TCN1 | Transcobalamin-1 | P20061 | no | Tbio | Transport of Vit. B12 |
| 42 | DBI | Acyl-CoA-binding protein | P07108 | yes | Tbio |  |
| 44 | CEACAM16 | Carcinoembryonic antigen-related cell adhesion molecule 16 | Q2WEN9 | yes | Tbio |  |
| 54 | ATP5F1A | ATP synthase subunit alpha, mitochondrial (Fragment) | ATP5F1A | yes | Tbio |  |
| 98 | MELTF | Melanotransferrin | P08582 | No, but iron can independently cross the blood-brain area when chelated by MELTF (216) 40% similarity whith otolith matrix protein-1 (OMP-1) (217) | Tbio | Iron transport |
| 109 | ARF1 | ADP ribosylation factor 1 | P84077 | Yes | Tchem |  |
| 148 | RAB21 | Ras-related protein Rab-21 | Q9UL25 | Yes | Tbio |  |
| 178 | RAB1B | Ras-related protein Rab-1B | E9PLD0 | Yes | Tbio |  |
| 192 | COPB2 | Coatomer subunit beta' | B4DZI8 | Yes | Tbio |  |

protease

| # | Gene | Protein | ID | Information about Cochlea | Druggability | Further information/ Literature |
| --- | --- | --- | --- | --- | --- | --- |
| 19 | PRSS8 | Prostasin | Q16651 | Yes | Tchem |  |
| 56 | AGA | Aspartylglucosaminidase | P20933 | Yes | Tbio | Protease/ hydrolase |
| 94 | IDE | Insulin-degrading enzyme | P14735 | No | Tchem | Protease/ hydrolase/  receptor |
| 110 | UCHL1 | Ubiquitin carboxyl-terminal hydrolase isozyme L1 | D6RE83 | Yes | Tchem | Protease hydrolase |
| 113 | PSMA2 | Proteasome subunit alpha type 2 | P25787 | Yes | Tbio | Protease hydrolase |
| 119 | MMP8 | Neutrophil collagenase | P22894 | Yes | Tchem | Protease hydrolase |
| 151 | YOD1 | Ubiquitin thioesterase OTU1 (Isoform 2) | Q5VVQ6-2 | No | Tbio | Protease hydrolase |
| 153 | CTSV | Cathepsin L2 | O60911 | No | Tchem, but 1 approved drug |  |
| 157 | YME1L1 | ATP-dependent zinc metalloprotease YME1L1 (Isoform 3) | Q96TA2-3 | Yes | Tbio | Protease hydrolase |
| 162 | KLK10 | Kallikrein 10 | O43240 | No | Tbio | Protease hydrolase  Tumor suppressor |
| 171 | PSMB9 | Proteasome subunit beta type-9 (Fragment) | B0V0T2 | Yes | Tchem | protease  hydrolase |
| 176 | ANPEP | Aminopeptidase N | P15144 | Yes | Tchem | Hydrolase protease receptor |
| 183 | PSMB3 | Proteasome subunit beta type-3 | P49720 | Yes | Tdark | Protease hydrolase |

hydrolase

| # | Gene | Protein | ID | Information about Cochlea | Druggability | Further information/ Literature |
| --- | --- | --- | --- | --- | --- | --- |
| 15 | DNASE1 | Deoxyribonuclease 1 | P24855 | Yes, aber wenig und ohne gut gesicherte Quellen | Tchem |  |
| 65 | GBA | Glucosylceramidase | J3KQG4 | Yes | Tclin | Hydrolase/ transferase |
| 116 | ASAH1 | Acid ceramidase | E7ERV9 | Yes | Tchem |  |
| 137 | GAA | Lysosomal alpha-glucosidase | P10253 | Yes | Tclin | Hydrolase (Glycosidase) |
| 141 | TREX2 | Three prime repair exonuclease 2 (Isoform 2) | Q9BQ50-2 | No | Tbio | Nuclease,  No isoform specific information |
| 154 | FAH | Fumaryacetoacetase | P16930 | No | Tbio | Tyrosine catabolism |
| 163 | RAB10 | Ras-related protein Rab-10 | P61026 | Yes | Tbio |  |
| 172 | DNPH1 | 2'-deoxynucleoside 5'-phosphate N-hydrolase 1 (Isoform 2) | O43598-2 | Yes | Tchem | Hydrolase, glycosidase |
| 174 | CDC42 | Cell division control protein 42 homolog | P60953 | Yes | Tchem |  |
| 188 | RNASE4 | Ribonuclease 4 | P34096 | Yes | Tbio | Nuclease |

phosphatase

| # | Gene | Protein | ID | Information about Cochlea | Druggability | Further information/ Literature |
| --- | --- | --- | --- | --- | --- | --- |
| 46 | PTPRF | Receptor-type tyrosine-protein phosphatase F (Isoform 2) | P10586-2 | no | Tchem | cell junction protein, transmembrane receptor, phosphatase  No isoformsprecific information. |

calcium-binding protein

| # | Gene | Protein | ID | Information about Cochlea | Druggability | Further information/ Literature |
| --- | --- | --- | --- | --- | --- | --- |
| 11 | CALML3 | Calmodulin-like protein 3 | P27482 | no | Tbio |  |
| 78 | S100P | Protein S100P | P25815 | Not specific, but s100 proteins are enriched in cochlea (218) | Tbio | Calcium sensor, stimulates cell proliferation |
| 142 | MYL12A | Myosin regulatory light chain 12A | P19105 | Yes | Tbio |  |
| 158 | ANXA4 | Annexin A4 | B4DDF9 | Yes | Tbio |  |

signaling molecule

| # | Gene | Protein | ID | Information about Cochlea | Druggability | Further information/ Literature |
| --- | --- | --- | --- | --- | --- | --- |
| 60 | GPHA2 | Glycoprotein hormone alpha-2 | Q96T91 | yes | Tbio | Hormone |
| 62 | LEFTY-1 | Left-right determination factor 1 | O75610 | no | Tbio | Cytokine, essential for left-right asymmetries |
| 100 | IL36RN | Interleukin-36 receptor antagonist protein (Fragment) | C9JTH1 | no | Tbio | ID-> no entry in Pharos, but through gene name  Cytokine, associated with psoriasis |
| 106 | RAC2 | Ras-related C3 botulinum toxin substrate 2 (Fragment) | B1AH78 | Yes | Tbio |  |
| 125 | THBS4 | Throbospondin-4 | P35443 | Yes | Tbio | calcium-binding protein  signaling molecule? (growth factor) |
| 186 | NDRG1 | Protein NDRG1 | E5RJY1 | Yes | Tbio |  |

transcription factor

| # | Gene | Protein | ID | Information about Cochlea | Druggability | Further information/ Literature |
| --- | --- | --- | --- | --- | --- | --- |
| 88 | MNDA | Myeloid cell nuclear differentiation antigen | P41218 | Yes | Tbio |  |
| 122 | PURA | Transcriptional activator protein Pur-alpha | Q00577 | No | Tbio |  |
| 169 | YBX3 | Y-box-binding protein 3 (Isoform 2) | P16989-2 | No | Tbio | transcription factor? (transcription regulation)  nucleic acid binding |
| 175 | MTPN | Myotrophin | C9JL85 | Yes | Tbio | ?? |
| 204 | GATA4 | Transcription factor GATA-4 (Fragment) | E9PKS4 | No, but: In spite of different origins from the cochlea (endoderm vs. ectoderm), the mammalian heart presents several similarities during regeneration, including inefficient cell reprograming, age-dependence, immature/nonfunctional cells. In fact, several groups have succeeded in converting cardiac fibroblasts to functional cardiomyocytes with an efficiency of 7%–20% by using a combination of multiple cardiac-lineage TFs (Gata4, Hand2, Mef2c, and Tbx5 or GHMT) ([Qian et al., 2012](https://www.sciencedirect.com/science/article/pii/S037859551630260X#bib157), [Song et al., 2012](https://www.sciencedirect.com/science/article/pii/S037859551630260X#bib173))  (187) | Tbio | nucleic acid binding  transcription factor (activator)  ID-> no entry in Pharos, but through gene name |

nucleic acid binding

| # | Gene | Protein | ID | Information about Cochlea | Druggability | Further information/ Literature |
| --- | --- | --- | --- | --- | --- | --- |
| 22 | HIST1H1B | Histone H1.5 | P16401 | no | Tbio | Linker histone, class 1 |
| 35 | RPLP2 | 60S acidic ribosomal protein P2 | P05387 | No, but one article where RPLP2 is used as a housekeeping gene and internal standard for expression changes in rat cochlea (Q: Determination of the apoptosis and cell survival signal transduction in the rat cochlea following neomycin induced deafness, Poster MHH) | Tbio |  |
| 66 | RPSA | 40S ribosomal protein SA (Fragment) | C9J9K3 | No | Tbio | ID-> no entry in Pharos, but through gene name  Required for the assembly and/or stability of the 40S ribosomal subunit  receptor |
| 74 | HNRNPK | Heterogeneous nuclear ribonucleoprotein K | Q5T6W5 | Yes | Tbio | transcription factor  nucleic acid binding |
| 87 | RPS18 | 40S ribosomal protein S18 | P62269 | No | Tbio | Ribosomal protein |
| 95 | RPL6 | 60S ribosomal protein L6 | Q02878 | No | Tbio | Ribosomal protein |
| 107 | HNRNPM | Heterogeneous nuclear ribonucleoprotein M (Isoform 2) | P52272-2 | No | Tbio | hnRNPs |
| 115 | SET | Protein SET, Isoform 3 | Q01105-3 | Yes | Tbio | Chaperone |
| 134 | SERBP1 | Plasminogen activator inhibitor 1 RNA-binding protein (Isoform 4) | Q8NC51-4 | Yes | Tbio |  |
| 135 | RPL8 | 60S ribosomal protein L8 | E9PP36 | Yes | Tbio | Ribonucleoprotein |
| 136 | HNRNPA1 | Heterogeneous nuclear ribonucleoprotein A1 (Fragment) | H0YH80 | Yes | Tchem |  |
| 143 | NOL9 | Polynucleotide 5'-hydroxyl-kinase NOL9 | Q5SY16 | No | Tdark | Involved in RNA processing |
| 145 | HNRNPA3 | Heterogeneous nuclear ribonucleoprotein A3 | E7EWI9 | Yes | Tbio |  |
| 146 | NBN | Nibrin | O60934 | Yes | Tbio | DNA repair |
| 152 | RPL29 | 60S ribosomal protein L29 | P47914 | No, but RPL29 is a lysine rich protein: Lysyl-tRNA synthetase 1 [KARS1 (MIM 601421)] encodes the enzyme that charges tRNALys with lysine in both the cytoplasm and the mitochondria [74]. Interestingly, bi-allelic KARS1 variants have been associated with recessive, nonsyndromic hearing impairment [73], suggesting that inner ear cells may be particularly sensitive to impairments in tRNA charging with lysine. (219) | Tbio | Ribosomal Protein |
| 182 | RPS9 | 40S ribosomal protein S9 | P46781 | Yes | Tbio | ribonucleoprotein |
| 187 | PCBP1 | Poly(rC)-binding protein 1 | Q15365 | No | Tbio | multifunctional |
| 198 | EIF3B | Eukaryotic translation initiation factor 3 subunit B | P55884 | No | Tbio |  |

transmembrane receptor

| # | Gene | Protein | ID | Information about Cochlea | Druggability | Further information/ Literature |
| --- | --- | --- | --- | --- | --- | --- |
| 7 | LPR2 | Low-density lipoprotein receptor related protein 2  (Megalin) | P98164 | Yes | Tbio |  |
| 27 | CKAP4 | Cytoskeleton-associated protein 4  (CLIMP63 | Q07065 | Yes | Tbio |  |
| 34 | CD9 | CD9 antigen | A6NNI4 | Yes | Tbio |  |
| 86 | GPRC5B | G-protein coupled receptor family C group 5 member B | Q9NZH0 | Yes | Tbio |  |

regulatory/adaptor protein kinase

| # | Gene | Protein | ID | Information about Cochlea | Druggability | Further information/ Literature |
| --- | --- | --- | --- | --- | --- | --- |
| 89 | FN3K | Fructosamine-3-kinase | Q9H479 | yes | Tbio |  |
| 118 | CKMT1A | Creatine-kinase U-Type mitochondrial | P12532 | Yes | Tbio |  |
| 164 | NAGK | N-acetyl-D-glucosamine kinase | Q9UJ70 | No | Tbio | Creates GlcNAc 6-phosphate |
| 166 | JAK1 | Janus Kinase 1 | P23458 | Yes | Tclin | transferase |
| 205 | CMPK1 | UMP-CMP kinase | E9PGI8 | Yes | Tbio | transferase |

ligase

| # | Gene | Protein | ID | Information about Cochlea | Druggability | Further information/ Literature |
| --- | --- | --- | --- | --- | --- | --- |
| 23 | GLUL | Glutamine synthetase | P15104 | Yes | Tchem | Ligase/ transferase |

transferase

| # | Gene | Protein | ID | Information about Cochlea | Druggability | Further information/ Literature |
| --- | --- | --- | --- | --- | --- | --- |
| 9 | TTN | Titin  (Isoform 5) | Q8WZ42-5 | yes | Tbio |  |
| 31 | TGM2 | Protein-glutamine gamma-glutamyltransferase 2 | B4DIT7  P21980 | Yes | Tchem |  |
| 47 | CKM | Creatine kinase M-type | P06732 | Yes | Tbio |  |
| 63 | TYMP | Thymidine phosphorylase | C9JGI3 | Yes | Tclin | transferase  signaling molecule |
| 76 | GSTM2 | Glutathione S-transferase Mu 2 | E9PHN7 | Yes | Tchem |  |
| 108 | ACLY | ATP-citrate synthase | B4E3P0 | Yes | Tclin |  |
| 124 | PYGB | Glycogen phosphorylase, brain form | P11216 | Yes | Tbio |  |
| 126 | MPST | 3-mercaptopyruvate sulfurtransferase | B1AH49 | Yes | Tchem |  |
| 130 | TGM5 | Protein-glutamine gamma-glutamyltransferase 5 (Isoform Short) | O43548-2 | No | Tbio | No isoform specific information  Catalyzes the cross-linking of proteins and the conjugation of polyamines to proteins (Q:Pharos) |
| 132 | APRT | Adenine phosphoribosyl-transferase | H3BQZ9 | Yes | Tbio |  |
| 149 | HPRT1 | Hypoxanthine-guanine phosphoribosyl-transferase | P00492 | Yes | Tchem |  |
| 156 | ACAT2 | Acetyl-CoA acetyltransferase, cytosolic | Q9BWD1 | Yes | Tchem |  |
| 173 | NAMPT | Nicotinamide phosphoribosyltransferase | P43490 | Yes | Tchem | signaling molecule (cytokine)  **transferase** |
| 181 | HMGCS1 | Hydroxymethylglutaryl-CoA synthase, cytoplasmic | Q01581 | Yes | Tchem | transferase  transcription factor? |
| 184 | ELOVL4 | Elongation of very long chain fatty acids protein 4 | Q9GZR5 | Yes | Tbio |  |
| 197 | NANS | Sialic acid synthase | Q9NR45 | Yes | Tbio |  |
| 202 | SULT2B1 | Sulfotransferase family cytosolic 2B member 1 | O00204 | No | Tbio | Catalyzes the sulfate conjugation of many hormones, neurotransmitters, drugs and xenobiotic compounds (Q:Pharos) |

lyase

| # | Gene | Protein | ID | Information about Cochlea | Druggability | Further information/ Literature |
| --- | --- | --- | --- | --- | --- | --- |
| 51 | CA3 | Carbonic anhydrase 3 | P07451 | Yes | Tclin |  |

oxidoreductase

| # | Gene | Protein | ID | Information about Cochlea | Druggability | Further information/ Literature |
| --- | --- | --- | --- | --- | --- | --- |
| 4 | CRYM | Ketamin reductase mu-cristallin | Q14894 | yes | Tbio |  |
| 32 | MDH2 | Malate dehydrogenase | G3XAL0 | Yes | Tchem |  |
| 71 | BVLRA | Biliverdin reductase A | P53004 | no | Tbio | Bilirubin metabolism |
| 75 | FTL | Ferritin light chain | P02792 | yes | Tbio |  |
| 101 | IDH1 | Isocitrate dehydrogenase [NADP] cytoplasmic | O75874 | Yes | Tclin |  |
| 120 | SORD | Sorbitol dehydrogenase | Q00796 | Yes | Tchem |  |
| 128 | PHGDH | D-3-phosphoglycerate dehydrogenase | Q5SZU1 | Yes | Tbio |  |
| 140 | ADH5 | Alcohol dehydrogenase class-3 | P11766 | Yes | Tchem |  |
| 147 | ADH1B | Alcohol dehydrogenase 1B | P00325 | No | Tclin | Alcohol metabolism |
| 150 | SDR9C7 | Short-chain dehydrogenase/reductase family 9C member 7 | Q8NEX9 | No | Tbio | Displays weak conversion of all-trans-retinal to all-trans-retinol in the presence of NADH |
| 155 | PRDX5 | Peroxiredoxin-5, mitochondrial (Isoform Cytoplasmic+peroxisomal) | P30044-2 | Yes | Tbio |  |
| 165 | AKR1A1 | Alcohol dehydrogenase [NADP(+)] | P14550 | Yes | Tchem |  |
| 167 | ALDH2 | Aldehyde dehydrogenase, mitochondrial | F8W0A9 | Yes | Tclin |  |
| 180 | GRHPR | Glyoxylate reductase/hydroxypyruvate reductase | Q9UBQ7 | Yes | Tbio |  |

Unknown

| # | Gene | Protein | ID | Information about Cochlea | Druggability | Further information/ Literature |
| --- | --- | --- | --- | --- | --- | --- |
| 18 | LEG1 | Protein LEG1 homolog | Q6P5S2 | no | Tbio | Developmental protein,  Almost no information at all (no OMIM, short Pharos) |
| 24 | CRYAB | Alpha-crystallin b-chain (Fragment | E9PR44  P02511 | Yes | Tbio | Chaperone |
| 28 | PGM1 | Phosphoglucomutase 1 | P36871 | Yes | Tbio | isomerase |
| 33 | P4HB | Protein disulfide-isomerase | P07237 | yes | Tbio | Chaperone/ isomerase |
| 50 | FHL1 | Four and a half LIM domains protein 1 | Q13642-1 | yes | Tbio | unknown |
| 59 | ATP5F1B | ATP synthase subunit beta, mitochondrial | P06576 | no | Tbio | Translocase  This gene encodes the beta subunit of the catalytic core. |
| 77 | C16orf89 | UPF0764 protein C16orf89 (Isoform 2) | Q6UX73-2 | no | Tbio | Thyroid protein for function and development |
| 85 | TAGLN2 | Transgelin 2 | P37802 | Yes | Tbio | Tumor supressor |
| 90 | LDB1 | LIM domain-binding protein 1 (Isoform 2) | Q86U70-3 | Yes | Tbio | Developmental protein |
| 91 | HSPA4 | Heat shock 70 kDa protein 4 | P34932 | Yes | Tbio | Stress response |
| 102 | AFP | Alpha-fetoprotein | P02771 | No, only that rat fetal cochlea lacks estrogen receptors, and alpha fetoprotein binds estrogen. (220) | Tbio | Metal binding, fetal protein |
| 114 | LMNB1 | Lamin-B1 | P20700 | Yes | Tbio | Maintain nuclear shape and mechanical integrity |
| 121 | CENPF | Centromere protein F | P49454 | Yes | Tbio |  |
| 131 | SKA1 | Spindle and kinetochore-associated protein 1 (Fragment) | K7EPW0 | No | Tbio | Microtubuli binding Protein |
| 133 | HIP1R | Huntingtin-interacting protein 1-related protein | O75146 | Yes | Tbio | Actin-binding |
| 199 | PDCD6IP | Programmed cell death 6-interacting protein | Q8WUM4 | Yes | Tbio | Transporter  Cell cycle protein  multifunctional |
| 200 | ZNF185 | Zinc finger protein 185 (Isoform 4) | O15231-4 | No | Tbio | May be involved in the regulation of cellular proliferation and/or differentiation (Q:Pharos) |

Literature:

(1) Hosoya MM. Distinct Expression Patterns Of Causative Genes Responsible For Hereditary Progressive Hearing Loss In Non-Human Primate Cochlea. Scientific Reports 2016;6(1):22250; 22250.

(2) Oshima AA. CRYM mutations cause deafness through thyroid hormone binding properties in the fibrocytes of the cochlea. J Med Genet 2006;43(6):e25; e25.

(3) Abe SS. Identification of CRYM as a candidate responsible for nonsyndromic deafness, through cDNA microarray analysis of human cochlear and vestibular tissues. The American Journal of Human Genetics 2003;72(1):73; 73-82; 82.

(4) Son EJ, Wu L, Yoon H, Kim S, Choi JY, Bok J. Developmental gene expression profiling along the tonotopic axis of the mouse cochlea. PLoS One 2012;7(7):e40735.

(5) Mutai HH. Gene expression dataset for whole cochlea of Macaca fascicularis. Scientific Reports 2018;8(1):15554; 15554.

(6) Mizuta KK. Ultrastructural localization of megalin in the rat cochlear duct. Hear Res 1999;129(1-2):83; 83-91; 91.

(7) Tauris JJ. Cubilin and megalin co-localize in the neonatal inner ear. Audiology and Neurotology 2009;14(4):267; 267-278; 278.

(8) König OO. Estrogen and the inner ear: megalin knockout mice suffer progressive hearing loss. The FASEB Journal 2008;22(2):410; 410-417; 417.

(9) Petitpré CC. Neuronal heterogeneity and stereotyped connectivity in the auditory afferent system. Nature Communications 2018;9(1):3691; 3691.

(10) Shin SSH. Differential Protein Expression in Congenital and Acquired Cholesteatomas. PLOS ONE 2015;10(9):e0137011; e0137011.

(11) Nishio SSY. Gene expression profiles of the cochlea and vestibular endorgans: localization and function of genes causing deafness. Annals of Otology, Rhinology&Laryngology 2015;124(1_suppl):6S; 6S-48S; 48S.

(12) Mhatre, Anand N A. N. Expression of Myh9 in the mammalian cochlea: localization within the stereocilia. J Neurosci Res 2006;84(4):809; 809-818; 818.

(13) Lalwani, A K A. K. Human nonsyndromic hereditary deafness DFNA17 is due to a mutation in nonmuscle myosin MYH9. The American Journal of Human Genetics 2000;67(5):1121; 1121-1128; 1128.

(14) Mhatre, Anand N A. N. Cloning and developmental expression of nonmuscle myosin IIA (Myh9) in the mammalian inner ear. J Neurosci Res 2004;76(3):296; 296-305; 305.

(15) Grandi, Fiorella Carla F. C. Single-Cell RNA Analysis of Type I Spiral Ganglion Neurons Reveals a Population in the Cochlea. Frontiers in Molecular Neuroscience 2020;13:83; 83.

(16) Smeti II. Transcriptomic analysis of the developing and adult mouse cochlear sensory epithelia. PLoS ONE 2012;7(8):e42987; e42987.

(17) Ebeid MM. Transcriptome-wide comparison of the impact of Atoh1 and miR-183 family on pluripotent stem cells and multipotent otic progenitor cells. PLOS ONE 2017;12(7):e0180855; e0180855.

(18) Broekaert DD. Keratinization of middle ear cholesteatomas. II. A histochemical study of epidermal transglutaminase substrates. European Archives of Oto-Rhino-Laryngology 1990;247(5):318; 318-322; 322.

(19) Chao, W Y W. Y. Expression of involucrin in human middle ear cholesteatoma. Am J Otol 1989;10(5):385; 385-388; 388.

(20) Regulation of the epithelial Na channel by peptidases. Current topics in developmental biology; 2007.

(21) Szabo RR. Membrane-anchored serine proteases as regulators of epithelial function. Biochem Soc Trans 2020;48(2):517; 517-528; 528.

(22) Nordang LL. Glutamate is the afferent neurotransmitter in the human cochlea. Acta Otolaryngol 2000;120(3):359; 359-362; 362.

(23) Sun, Y W Y. W. Effects of L-glutamine, glutaminase and glutamine synthetase on CAP threshold of cochlear nerve of guinea pig. Science in China.Series B, Chemistry, life sciences & earth sciences 1991;34(2):184; 184-193; 193.

(24) Eybalin MM. Glutamine synthetase and glutamate metabolism in the guinea pig cochlea. Hear Res 1996;101(1-2):93; 93-101; 101.

(25) Cheng CC. Age-related transcriptome changes in Sox2 supporting cells in the mouse cochlea. Stem Cell Research&Therapy 2019;10(1):365; 365.

(26) Erni, Silvia T S. T. Anti-inflammatory and Oto-Protective Effect of the Small Heat Shock Protein Alpha B-Crystallin (HspB5) in Experimental Pneumococcal Meningitis. Frontiers in Neurology 2019;10:570; 570.

(27) Scheffer, Déborah I D. I. Gene Expression by Mouse Inner Ear Hair Cells during Development. Journal of Neuroscience 2015;35(16):6366; 6366-6380; 6380.

(28) Karasawa TT. CLIMP-63 is a gentamicin-binding protein that is involved in drug-induced cytotoxicity. Cell Death&Disease 2010;1(11):e102; e102.

(29) Wong, Eugene H C E. H. C. Inner ear exosomes and their potential use as biomarkers. PLOS ONE 2018;13(6):e0198029; e0198029.

(30) Herget MM. A simple method for purification of vestibular hair cells and non-sensory cells, and application for proteomic analysis. PLoS ONE 2013;8(6):e66026; e66026.

(31) Huang, R Stephanie R. S. Identification of genetic variants contributing to cisplatin-induced cytotoxicity by use of a genomewide approach. The American Journal of Human Genetics 2007;81(3):427; 427-437; 437.

(32) Nell, M J M. J. Bactericidal/permeability-increasing protein prevents mucosal damage in an experimental rat model of chronic otitis media with effusion. Infect Immun 2000;68(5):2992; 2992-2994; 2994.

(33) Nell, M J M. J. Efficacy of bactericidal/permeability-increasing protein in experimental otitis media with effusion in rats: a new therapy for mucosal infections. J Lab Clin Med 2001;137(4):303; 303-309; 309.

(34) Liu YYY. Thyroid hormone receptor isoform-specific modification by small ubiquitin-like modifier (SUMO) modulates thyroid hormone-dependent gene regulation. J Biol Chem 2012;287(43):36499; 36499-36508; 36508.

(35) Spinelli, Kateri J K. J. Distinct energy metabolism of auditory and vestibular sensory epithelia revealed by quantitative mass spectrometry using MS2 intensity. Proceedings of the National Academy of Sciences 2012;109(5):E268; E268-77; E277.

(36) Sajan, Samin A S. A. Toward a systems biology of mouse inner ear organogenesis: gene expression pathways, patterns and network analysis. Genetics 2007;177(1):631; 631-653; 653.

(37) Coling, Donald E D. E. Proteomic analysis of cisplatin-induced cochlear damage: methods and early changes in protein expression. Hear Res 2007;226(1-2):140; 140-156; 156.

(38) Hartman, Byron H B. H. Identification and characterization of mouse otic sensory lineage genes. Frontiers in Cellular Neuroscience 2015;9:79; 79.

(39) Ranum, Paul T P. T. Insights into the Biology of Hearing and Deafness Revealed by Single-Cell RNA Sequencing. Cell Reports 2019;26(11):3160; 3160-3171.e3.

(40) Zwaenepoel II. Otoancorin, an inner ear protein restricted to the interface between the apical surface of sensory epithelia and their overlying acellular gels, is defective in autosomal recessive deafness DFNB22. Proceedings of the National Academy of Sciences 2002;99(9):6240; 6240-6245; 6245.

(41) Sathyanarayana, Bangalore K B. K. Mesothelin, Stereocilin, and Otoancorin are predicted to have superhelical structures with ARM-type repeats. BMC Structural Biology 2009;9(1):1; 1.

(42) Wang YY. Blast-induced hearing impairment in rats is associated with structural and molecular changes of the inner ear. Scientific Reports 2020;10(1):10652; 10652.

(43) Chen ZZ. Identification of nasopharyngeal carcinoma metastasis-related biomarkers by iTRAQ combined with 2D-LC-MS/MS. Oncotarget 2016;7(23):34022; 34022-34037; 34037.

(44) Samuels, Tina L T. L. Association of Gel-Forming Mucins and Aquaporin Gene Expression With Hearing Loss, Effusion Viscosity, and Inflammation in Otitis Media With Effusion. JAMA Otolaryngology–Head&Neck Surgery 2017;143(8):810; 810-817; 817.

(45) Roy, Michelle G M. G. Muc5b is required for airway defence. Nature 2014;505(7483):412; 412-416; 416.

(46) Kawano HH. Identification of MUC5B mucin gene in human middle ear with chronic otitis media. Laryngoscope 2000;110(4):668; 668-673; 673.

(47) Lin JJ. Expression of mucins in mucoid otitis media. JARO - Journal of the Association for Research in Otolaryngology 2003;4(3):384; 384-393; 393.

(48) Gong, Tzy-Wen L T. W. Age-related changes in cochlear gene expression in normal and shaker 2 mice. Journal of the Association for Research in Otolaryngology 2006;7(3):317; 317-328; 328.

(49) Hickox, Ann E A. E. Global Analysis of Protein Expression of Inner Ear Hair Cells. The Journal of Neuroscience 2017;37(5):1320; 1320-1339; 1339.

(50) Yang SS. Variation analysis of transcriptome changes reveals cochlear genes and their associated functions in cochlear susceptibility to acoustic overstimulation. Hear Res 2015;330(Pt A):78; 78-89; 89.

(51) Zheng JJ. Carcinoembryonic antigen-related cell adhesion molecule 16 interacts with alpha-tectorin and is mutated in autosomal dominant hearing loss (DFNA4). Proceedings of the National Academy of Sciences 2011;108(10):4218; 4218-4223; 4223.

(52) Cheatham, Mary Ann M. A. Loss of the tectorial membrane protein CEACAM16 enhances spontaneous, stimulus-frequency, and transiently evoked otoacoustic emissions. Journal of Neuroscience 2014;34(31):10325; 10325-10338; 10338.

(53) Kammerer RR. Loss of mammal-specific tectorial membrane component carcinoembryonic antigen cell adhesion molecule 16 (CEACAM16) leads to hearing impairment at low and high frequencies. J Biol Chem 2012;287(26):21584; 21584-21598; 21598.

(54) Goodyear, Richard J R. J. Extracellular matrices associated with the apical surfaces of sensory epithelia in the inner ear: molecular and structural diversity. J Neurobiol 2002;53(2):212; 212-227; 227.

(55) Khetarpal UU. Expression and localization of COL2A1 mRNA and type II collagen in human fetal cochlea. Hear Res 1994;79(1-2):59; 59-73; 73.

(56) Acke, Frederic R E F. R. Hearing impairment in Stickler syndrome: a systematic review. Orphanet Journal of Rare Diseases 2012;7(1):84; 84.

(57) Spicer, S S S. S. Creatine kinase in epithelium of the inner ear. Journal of Histochemistry&Cytochemistry 1992;40(2):185; 185-192; 192.

(58) Wong, Ann Chi Yan A. C. Expression and distribution of creatine transporter and creatine kinase (brain isoform) in developing and mature rat cochlear tissues. Histochem Cell Biol 2012;137(5):599; 599-613; 613.

(59) Shin JB, Streijger F, Beynon A, Peters T, Gadzala L, McMillen D, et al. Hair bundles are specialized for ATP delivery via creatine kinase. Neuron 2007 February 01;53(3):371-386.

(60) Wallimann TT. The creatine kinase system and pleiotropic effects of creatine. Amino Acids 2011;40(5):1271; 1271-1296; 1296.

(61) Minami SB, Yamashita D, Ogawa K, Schacht J, Miller JM. Creatine and tempol attenuate noise-induced hearing loss. Brain Res 2007 May 07;1148:83-89.

(62) Shpargel, Karl B K. B. Col11a1 and Col11a2 mRNA expression in the developing mouse cochlea: implications for the correlation of hearing loss phenotype with mutant type XI collagen genotype. Acta Otolaryngol 2004;124(3):242; 242-248; 248.

(63) McGuirt, W T W. T. Mutations in COL11A2 cause non-syndromic hearing loss (DFNA13). Nat Genet 1999;23(4):413; 413-419; 419.

(64) Hawkins RD, Bashiardes S, Powder KE, Sajan SA, Bhonagiri V, Alvarado DM, et al. Large scale gene expression profiles of regenerating inner ear sensory epithelia. PLoS One 2007 June 13;2(6):e525.

(65) Wu LL. A systematic survey of carbonic anhydrase mRNA expression during mammalian inner ear development. Developmental Dynamics 2013;242(3):269; 269-280; 280.

(66) Ikeda KK. Early effects of acetazolamide on anionic activities of the guinea pig endolymph: evidence for active function of carbonic anhydrase in the cochlea. Hear Res 1987;31(3):211; 211-216; 216.

(67) Prazma JJ. Carbonic anhydrase in the generation of cochlear potentials. American Journal of Physiology-Renal Physiology 1978;235(4):F317; F317-20; F320.

(68) Weber, P C P. C. Potassium recycling pathways in the human cochlea. Laryngoscope 2001;111(7):1156; 1156-1165; 1165.

(69) Du TTT. LMO7 deficiency reveals the significance of the cuticular plate for hearing function. Nature Communications 2019;10(1):1117; 1117.

(70) Maass, Juan C J. C. Transcriptomic Analysis of Mouse Cochlear Supporting Cell Maturation Reveals Large-Scale Changes in Notch Responsiveness Prior to the Onset of Hearing. PLOS ONE 2016;11(12):e0167286; e0167286.

(71) Gonzalez-Gomez II. Progressive neurodegeneration in aspartylglycosaminuria mice. The American Journal of Pathology 1998;153(4):1293; 1293-1300; 1300.

(72) Klockars TT. In silico analyses of mouse inner-ear transcripts. JARO - Journal of the Association for Research in Otolaryngology 2003;4(1):24; 24-40; 40.

(73) Munnamalai VV. Wnt9a Can Influence Cell Fates and Neural Connectivity across the Radial Axis of the Developing Cochlea. The Journal of Neuroscience 2017;37(37):8975; 8975-8988; 8988.

(74) Beisel, K W K. W. Identification of novel alternatively spliced isoforms of the tropomyosin-encoding gene, TMnm, in the rat cochlea. Gene 1994;143(2):251; 251-256; 256.

(75) Slepecky NN. Tropomyosin co-localizes with actin microfilaments and microtubules within supporting cells of the inner ear. Cell Tissue Res 1987;248(1):63; 63-66; 66.

(76) Maeda YY. Microarray analysis of the effect of dexamethasone on murine cochlear explants. Acta Otolaryngol 2010;130(12):1329; 1329-1334; 1334.

(77) Li JJN. Successful cochlear implantation in a patient with MNGIE syndrome. Acta Otolaryngol 2011;131(9):1012; 1012-1016; 1016.

(78) Nishino II. Thymidine phosphorylase gene mutations in MNGIE, a human mitochondrial disorder. Science 1999;283(5402):689; 689-692; 692.

(79) Hiraki NN. Mitochondrial neurogastrointestinal encephalomyopathy associated with progressive hearing loss. The Journal of Laryngology&Otology 2010;124(9):1007; 1007-1009; 1009.

(80) Yadak RR. Mitochondrial Neurogastrointestinal Encephalomyopathy Caused by Thymidine Phosphorylase Enzyme Deficiency: From Pathogenesis to Emerging Therapeutic Options. Frontiers in Cellular Neuroscience 2017;11:31; 31.

(81) Endo SS. A case of improved hearing with cochlear implantation in Gaucher disease type 1. Auris Nasus Larynx 2018;45(3):603; 603-607; 607.

(82) Wang PP. Identification of sequence variants associated with severe microtia-astresia by targeted sequencing. BMC Medical Genomics 2019;12(1):28; 28.

(83) Bas-Orth CC. The divergence-convergence model of acquired neuroprotection. Mech Dev 2013;130(6-8):396; 396-401; 401.

(84) Hashimoto S, Billings P, Harris JP, Firestein GS, Keithley EM. Innate immunity contributes to cochlear adaptive immune responses. Audiol Neurootol 2005 February 01;10(1):35-43.

(85) Cai Q, Vethanayagam RR, Yang S, Bard J, Jamison J, Cartwright D, et al. Molecular profile of cochlear immunity in the resident cells of the organ of Corti. J Neuroinflammation 2014 October 14;11:173-8.

(86) Yamada T, Ogi K, Sakashita M, Kanno M, Kubo S, Ito Y, et al. Toll-like receptor ligands induce cytokine and chemokine production in human inner ear endolymphatic sac fibroblasts. Auris Nasus Larynx 2017 August 01;44(4):398-403.

(87) Vittal PP. BCAP31 Mutation Causing a Syndrome of Congenital Dystonia, Facial Dysorphism and Central Hypomyelination Discovered Using Exome Sequencing. Movement Disorders Clinical Practice 2016;3(2):197; 197-199; 199.

(88) Au, P Y Billie P. Y. B. GeneMatcher aids in the identification of a new malformation syndrome with intellectual disability, unique facial dysmorphisms, and skeletal and connective tissue abnormalities caused by de novo variants in HNRNPK. Hum Mutat 2015;36(10):1009; 1009-1014; 1014.

(89) Noble, Kenyaria V K. V. Use of Proteomic Imaging Coupled With Transcriptomic Analysis to Identify Biomolecules Responsive to Cochlear Injury. Frontiers in Molecular Neuroscience 2018;11:243; 243.

(90) Nimpf SS. Subcellular analysis of pigeon hair cells implicates vesicular trafficking in cuticulosome formation and maintenance. eLife 2017;6.

(91) Santos-Sacchi JJ. A ferritin-containing cell type in the stria vascularis of the mouse inner ear. Acta Otolaryngol 1985;100(1-2):26; 26-32; 32.

(92) Castiglione AA. Sudden sensorineural hearing loss and polymorphisms in iron homeostasis genes: new insights from a case-control study. BioMed Research International 2015;2015:834736; 1.

(93) Park HHJ. GSTA4 mediates reduction of cisplatin ototoxicity in female mice. Nature Communications 2019;10(1):4150; 4150.

(94) Werner, Hauke B H. B. A critical role for the cholesterol-associated proteolipids PLP and M6B in myelination of the central nervous system. Glia 2013;61(4):567; 567-586; 586.

(95) Yang SS. Immune defense is the primary function associated with the differentially expressed genes in the cochlea following acoustic trauma. Hear Res 2016;333:283; 283-294; 294.

(96) Shrestha, Brikha R B. R. Sensory Neuron Diversity in the Inner Ear Is Shaped by Activity. Cell 2018;174(5):1229; 1229-1246.e17.

(97) Funk, Steven D S. D. Pathogenicity of a Human Laminin 2 Mutation Revealed in Models of Alport Syndrome. Journal of the American Society of Nephrology 2018;29(3):949; ASN.2017090997-960; 960.

(98) Zang HH. Roles of microRNAs in the resistance to platinum based chemotherapy in the non-small cell lung cancer. Journal of Cancer 2017;8(18):3856; 3856-3861; 3861.

(99) Zhao ZZ. miR-15b regulates cisplatin resistance and metastasis by targeting PEBP4 in human lung adenocarcinoma cells. Cancer Gene Ther 2015;22(3):108; 108-114; 114.

(100) Wan GG. Transient auditory nerve demyelination as a new mechanism for hidden hearing loss. Nature Communications 2017;8(1):14487; 14487.

(101) McLean, Will J W. J. Distinct capacity for differentiation to inner ear cell types by progenitor cells of the cochlea and vestibular organs. Development 2016;143(23):4381; 4381-4393; 4393.

(102) Darville, Lancia N F L. N. F. Label-free quantitative mass spectrometry analysis of differential protein expression in the developing cochlear sensory epithelium. Proteome Science 2018;16(1):15; 15.

(103) Lu, Cindy C C. C. Developmental profiling of spiral ganglion neurons reveals insights into auditory circuit assembly. Journal of Neuroscience 2011;31(30):10903; 10903-10918; 10918.

(104) Frye, Mitchell D M. D. Inflammation associated with noise-induced hearing loss. J Acoust Soc Am 2019;146(5):4020; 4020.

(105) Elkon RR. RFX transcription factors are essential for hearing in mice. Nature Communications 2015;6(1):8549; 8549.

(106) He DD. Transcription factor Isl1 is dispensable for the development of the mouse prosensory region. Cytotechnology 2020;72(3):407; 407-414; 414.

(107) Jamesdaniel SS. Downstream targets of Lmo4 are modulated by cisplatin in the inner ear of Wistar rats. PLoS ONE 2014;9(12):e115263; e115263.

(108) Jamesdaniel SS. Targeting nitrative stress for attenuating cisplatin-induced downregulation of cochlear LIM domain only 4 and ototoxicity. Redox Biology 2016;10:257; 257-265; 265.

(109) May, Lindsey A L. A. Inner ear supporting cells protect hair cells by secreting HSP70. J Clin Invest 2013;123(8):3577; 3577-3587; 3587.

(110) Cunningham, Lisa L L. L. Heat shock inhibits both aminoglycoside- and cisplatin-induced sensory hair cell death. Journal of the Association for Research in Otolaryngology 2006;7(3):299; 299-307; 307.

(111) Taleb MM. Hsp70 inhibits aminoglycoside-induced hair cell death and is necessary for the protective effect of heat shock. Journal of the Association for Research in Otolaryngology 2008;9(3):277; 277-289; 289.

(112) Yu YY. Geldanamycin induces production of heat shock protein 70 and partially attenuates ototoxicity caused by gentamicin in the organ of Corti explants. J Biomed Sci 2009;16(1):79; 79.

(113) Tebo, A E A. E. Antibody reactivity to heat shock protein 70 and inner ear-specific proteins in patients with idiopathic sensorineural hearing loss. Clin Exp Immunol 2006;146(3):427; 427-432; 432.

(114) Neely, J G J. G. Detection and localization of heat shock protein 70 in the normal guinea pig cochlea. Hear Res 1991;52(2):403; 403-406; 406.

(115) Rauch, S D S. D. Anti-heat shock protein 70 antibodies in Meniere's disease. Laryngoscope 2000;110(9):1516; 1516-1521; 1521.

(116) Schmitt HH. Heat Shock Proteins in Human Perilymph: Implications for Cochlear Implantation. Otology&Neurotology 2018;39(1):37; 37-44; 44.

(117) Ladrech SS. High mobility group box 1 (HMGB1): dual functions in the cochlear auditory neurons in response to stress? Histochem Cell Biol 2017;147(3):307; 307-316; 316.

(118) Ladrech SS. Supporting cells regulate the remodelling of aminoglycoside-injured organ of Corti, through the release of high mobility group box 1. Eur J Neurosci 2013;38(6):2962; n/a-2972; 2972.

(119) Bellussi, Luisa Maria L. M. High-mobility group box protein 1 expression in inflammatory diseases of the middle ear. Int J Immunopathol Pharmacol 2017;30(2):168; 168-173; 173.

(120) Chen LL. Molecular mechanisms underlying the protective effects of hydrogen-saturated saline on noise-induced hearing loss. Acta Otolaryngol 2017;137(10):1063; 1063-1068; 1068.

(121) Szczepanski, Miroslaw J M. J. Molecular signaling of the HMGB1/RAGE axis contributes to cholesteatoma pathogenesis. Journal of Molecular Medicine 2015;93(3):305; 305-314; 314.

(122) Clendenon, Sherry G S. G. Cadherin-11 controls otolith assembly: evidence for extracellular cadherin activity. Developmental Dynamics 2009;238(8):1909; 1909-1922; 1922.

(123) Kiyama YY. The adhesion molecule cadherin 11 is essential for acquisition of normal hearing ability through middle ear development in the mouse. Laboratory Investigation 2018;98(11):1364; 1364-1374; 1374.

(124) Salam, A A A. A. A novel locus (DFNA23) for prelingual autosomal dominant nonsyndromic hearing loss maps to 14q21-q22 in a Swiss German kindred. The American Journal of Human Genetics 2000;66(6):1984; 1984-1988; 1988.

(125) Wells, Helena R R H. R. R. GWAS Identifies 44 Independent Associated Genomic Loci for Self-Reported Adult Hearing Difficulty in UK Biobank. The American Journal of Human Genetics 2019;105(4):788; 788-802; 802.

(126) Ma WW. Ginkgolide B protects against cisplatin-induced ototoxicity: enhancement of Akt-Nrf2-HO-1 signaling and reduction of NADPH oxidase. Cancer Chemother Pharmacol 2015;75(5):949; 949-959; 959.

(127) Bánfi BB. NOX3, a superoxide-generating NADPH oxidase of the inner ear. J Biol Chem 2004;279(44):46065; 46065-46072; 46072.

(128) Kim YYR. Expression patterns of members of the isocitrate dehydrogenase gene family in murine inner ear. Biotechnic&Histochemistry 2017;92(7):536; 536-544; 544.

(129) Tadros, Sherif F S. F. Gene expression changes for antioxidants pathways in the mouse cochlea: relations to age-related hearing deficits. PLoS ONE 2014;9(2):e90279; e90279.

(130) Jamesdaniel SS. Chronic lead exposure induces cochlear oxidative stress and potentiates noise-induced hearing loss. Toxicol Lett 2018;292:175; 175-180; 180.

(131) White KK. Deficiency Does Not Affect the Cytosolic Glutathione or Thioredoxin Antioxidant Defense in Mouse Cochlea. The Journal of Neuroscience 2017;37(23):5770; 5770-5781; 5781.

(132) Grimsley-Myers, Cynthia M C. M. Redundant functions of Rac GTPases in inner ear morphogenesis. Dev Biol 2012;362(2):172; 172-186; 186.

(133) Lagresle-Peyrou CC. A gain-of-function RAC2 mutation is associated with bone-marrow hypoplasia and an autosomal dominant form of severe combined immunodeficiency. Haematologica 2020:haematol.2019.230250.

(134) Zallocchi MM. Regulated vesicular trafficking of specific PCDH15 and VLGR1 variants in auditory hair cells. Journal of Neuroscience 2012;32(40):13841; 13841-13859; 13859.

(135) Krey, Jocelyn F J. F. ELMOD1 Stimulates ARF6-GTP Hydrolysis to Stabilize Apical Structures in Developing Vestibular Hair Cells. The Journal of Neuroscience 2018;38(4):843; 843-857; 857.

(136) Kim, Yeon Ju Y. J. Downregulated UCHL1 Accelerates Gentamicin-Induced Auditory Cell Death via Autophagy. Mol Neurobiol 2019;56(11):7433; 7433-7447; 7447.

(137) Zhang YY. Role of the Ubiquitin C-Terminal Hydrolase L1-Modulated Ubiquitin Proteasome System in Auditory Cortex Senescence. ORL 2017;79(3):153; 153-163; 163.

(138) Cai TT. Characterization of the transcriptome of nascent hair cells and identification of direct targets of the Atoh1 transcription factor. Journal of Neuroscience 2015;35(14):5870; 5870-5883; 5883.

(139) Shin JJB. Molecular architecture of the chick vestibular hair bundle. Nat Neurosci 2013;16(3):365; 365-374; 374.

(140) Hu, Bo Hua B. H. Metalloproteinases and their associated genes contribute to the functional integrity and noise-induced damage in the cochlear sensory epithelium. Journal of Neuroscience 2012;32(43):14927; 14927-14941; 14941.

(141) Bhargava SS. Hyperhomocysteinemia, MMPs and Cochlear Function: A Short Review. Indian Journal of Clinical Biochemistry 2016;31(2):148; 148-151; 151.

(142) Eisner LL. The Balance of Tissue Inhibitor of Metalloproteinase-1 and Matrix Metalloproteinase-9 in the Autoimmune Inner Ear Disease Patients. Journal of Interferon&Cytokine Research 2017;37(8):354; 354-361; 361.

(143) Du ZZ. Differential Expression of LaminB1 in the Developing Rat Cochlea. The Journal of International Advanced Otology 2019;15(1):106; 106-111; 111.

(144) Sokolowski BB. Conserved BK channel-protein interactions reveal signals relevant to cell death and survival. PLoS ONE 2011;6(12):e28532; e28532.

(145) Andrews, C D C. D. Identification of a gene set to evaluate the potential effects of loud sounds from seismic surveys on the ears of fishes: a study with Salmo salar. J Fish Biol 2014;84(6):1793; 1793-1819; 1819.

(146) Yu, Fabian P S F. P. S. Acid ceramidase deficiency: Farber disease and SMA-PME. Orphanet Journal of Rare Diseases 2018;13(1):121; 121.

(147) Gan, Joanna J J. J. Acid ceramidase deficiency associated with spinal muscular atrophy with progressive myoclonic epilepsy. Neuromuscular Disorders 2015;25(12):959; 959-963; 963.

(148) Kirjavainen AA. The Rho GTPase Cdc42 regulates hair cell planar polarity and cellular patterning in the developing cochlea. Biology Open 2015;4(4):516; 516-526; 526.

(149) Rink JJ. Rab conversion as a mechanism of progression from early to late endosomes. Cell 2005;122(5):735; 735-749; 749.

(150) Zhang YY. Sensorineural deafness and male infertility: a contiguous gene deletion syndrome. J Med Genet 2007;44(4):233; 233-240; 240.

(151) Rezende, Carlos Eduardo Borges C. E. Cholesteatoma gene expression of matrix metalloproteinases and their inhibitors by RT-PCR. Brazilian journal of otorhinolaryngology 2012;78(3):116; 116-121; 121.

(152) Lauhio AA. Matrix metalloproteinase-8/collagenase-2 in childhood otitis media with effusion. Ann Med 2012;44(1):93; 93-99; 99.

(153) Wu JJ. Matrix metalloproteinase-2 and -9 contribute to functional integrity and noise‑induced damage to the blood-labyrinth-barrier. Molecular Medicine Reports 2017;16(2):1731; 1731-1738; 1738.

(154) SCHWARTZ EE. Consumption of a high-galactose diet induces diabetic-like changes in the inner ear. Otolaryngology - Head and Neck Surgery 1995;113(6):748; 748-754; 754.

(155) Huang MM. Overlapping and distinct pRb pathways in the mammalian auditory and vestibular organs. Cell Cycle 2011;10(2):337; 337-351; 351.

(156) Warchol, Mark E M. E. ADAM10 and γ-secretase regulate sensory regeneration in the avian vestibular organs. Dev Biol 2017;428(1):39; 39-51; 51.

(157) Ebrahim SS. Stereocilia-staircase spacing is influenced by myosin III motors and their cargos espin-1 and espin-like. Nature Communications 2016;7(1):10833; 10833.

(158) Mendus DD. Thrombospondins 1 and 2 are important for afferent synapse formation and function in the inner ear. Eur J Neurosci 2014;39(8):1256; 1256-1267; 1267.

(159) Hu JJG. Altered gene expression profile in a rat model of gentamicin-induced ototoxicity and nephrotoxicity, and the potential role of upregulated Ifi44 expression. Molecular Medicine Reports 2017;16(4):4650; 4650-4658; 4658.

(160) Bhargava SS. MMP-9 gene ablation mitigates hyperhomocystenemia-induced cognition and hearing dysfunction. Mol Biol Rep 2014;41(8):4889; 4889-4898; 4898.

(161) Mittal RR. Association of PRPS1 Mutations with Disease Phenotypes. Dis Markers 2015;2015:127013; 1.

(162) Liu, Xue Zhong X. Z. Hearing loss and PRPS1 mutations: Wide spectrum of phenotypes and potential therapy. International Journal of Audiology 2013;52(1):23; 23-28; 28.

(163) Low WWK. Ototoxicity from combined Cisplatin and radiation treatment: an in vitro study. International Journal of Otolaryngology 2010;2010:523976; 1.

(164) Liu LLM. Characterization of the transcriptomes of -induced hair cells in the mouse cochlea. American journal of stem cells 2020;9(1):1; 1-15; 15.

(165) Horn, Henning F H. F. The LINC complex is essential for hearing. J Clin Invest 2013;123(2):740; 740-750; 750.

(166) Kamphoven, Joep H J J. H. Hearing loss in infantile Pompe's disease and determination of underlying pathology in the knockout mouse. Neurobiol Dis 2004;16(1):14; 14-20; 20.

(167) van der Beek, Nadine A M E N. A. Hearing in adults with Pompe disease. J Inherit Metab Dis 2012;35(2):335; 335-341; 341.

(168) Cai QQ. Transcriptional changes in adhesion-related genes are site-specific during noise-induced cochlear pathogenesis. Neurobiol Dis 2012;45(2):723; 723-732; 732.

(169) Weber UU. APC/C-Dependent Regulation of Planar Cell Polarity Establishment via Nek2 Kinase Acting on Dishevelled. Developmental Cell 2017;40(1):53; 53-66; 66.

(170) de Vries II. Detection of BDNF-Related Proteins in Human Perilymph in Patients With Hearing Loss. Frontiers in Neuroscience 2019;13:214; 214.

(171) Kwon DDN. Oxidative stress and ROS metabolism via down-regulation of sirtuin 3 expression in Cmah-null mice affect hearing loss. Aging 2015;7(8):579; 579-594; 594.

(172) Zhang JJ. Cmah deficiency may lead to age-related hearing loss by influencing miRNA-PPAR mediated signaling pathway. PeerJ 2019;7:e6856; e6856.

(173) Sinkkonen ST, Starlinger V, Galaiya DJ, Laske RD, Myllykangas S, Oshima K, et al. Serial analysis of gene expression in the chicken otocyst. J Assoc Res Otolaryngol 2011 December 01;12(6):697-710.

(174) Val S, Krueger A, Poley M, Cohen A, Brown K, Panigrahi A, et al. Nontypeable Haemophilus influenzae lysates increase heterogeneous nuclear ribonucleoprotein secretion and exosome release in human middle-ear epithelial cells. FASEB J 2018 April 01;32(4):1855-1867.

(175) Tower-Gilchrist C, Zlatic SA, Yu D, Chang Q, Wu H, Lin X, et al. Adaptor protein-3 complex is required for Vangl2 trafficking and planar cell polarity of the inner ear. Mol Biol Cell 2019 August 15;30(18):2422-2434.

(176) Melgar-Rojas P, Alvarado JC, Fuentes-Santamaria V, Gabaldon-Ull MC, Juiz JM. Validation of Reference Genes for RT-qPCR Analysis in Noise-Induced Hearing Loss: A Study in Wistar Rat. PLoS One 2015 September 14;10(9):e0138027.

(177) Tanaka C, Coling DE, Manohar S, Chen GD, Hu BH, Salvi R, et al. Expression pattern of oxidative stress and antioxidant defense-related genes in the aging Fischer 344/NHsd rat cochlea. Neurobiol Aging 2012 August 01;33(8):1842.e1-1842.14.

(178) Cristobal RR. Assessment of differential gene expression in vestibular epithelial cell types using microarray analysis. Mol Brain Res 2005;133(1):19; 19-36; 36.

(179) Hartmann B, Wai T, Hu H, MacVicar T, Musante L, Fischer-Zirnsak B, et al. Homozygous YME1L1 mutation causes mitochondriopathy with optic atrophy and mitochondrial network fragmentation. Elife 2016 August 06;5:10.7554/eLife.16078.

(180) McInturff S, Burns JC, Kelley MW. Characterization of spatial and temporal development of Type I and Type II hair cells in the mouse utricle using new cell-type-specific markers. Biol Open 2018 November 19;7(11):10.1242/bio.038083.

(181) Huang M, Sage C, Tang Y, Lee SG, Petrillo M, Hinds PW, et al. Overlapping and distinct pRb pathways in the mammalian auditory and vestibular organs. Cell Cycle 2011 January 15;10(2):337-351.

(182) Lewis MA, Buniello A, Hilton JM, Zhu F, Zhang WI, Evans S, et al. Exploring regulatory networks of miR-96 in the developing inner ear. Sci Rep 2016 March 18;6:23363.

(183) Burns JC, Kelly MC, Hoa M, Morell RJ, Kelley MW. Single-cell RNA-Seq resolves cellular complexity in sensory organs from the neonatal inner ear. Nat Commun 2015 October 15;6:8557.

(184) Duncker SV, Franz C, Kuhn S, Schulte U, Campanelli D, Brandt N, et al. Otoferlin couples to clathrin-mediated endocytosis in mature cochlear inner hair cells. J Neurosci 2013 May 29;33(22):9508-9519.

(185) Chen Q, Quan Y, Wang N, Xie C, Ji Z, He H, et al. Inactivation of STAT3 Signaling Impairs Hair Cell Differentiation in the Developing Mouse Cochlea. Stem Cell Reports 2017 July 11;9(1):231-246.

(186) Jiang L, Romero-Carvajal A, Haug JS, Seidel CW, Piotrowski T. Gene-expression analysis of hair cell regeneration in the zebrafish lateral line. Proc Natl Acad Sci U S A 2014 April 08;111(14):1383.

(187) Zheng F, Zuo J. Cochlear hair cell regeneration after noise-induced hearing loss: Does regeneration follow development? Hear Res 2017 June 01;349:182-196.

(188) Gao X, Yuan YY, Lin QF, Xu JC, Wang WQ, Qiao YH, et al. Mutation of IFNLR1, an interferon lambda receptor 1, is associated with autosomal-dominant non-syndromic hearing loss. J Med Genet 2018 May 01;55(5):298-306.

(189) Hertzano R, Puligilla C, Chan SL, Timothy C, Depireux DA, Ahmed Z, et al. CD44 is a marker for the outer pillar cells in the early postnatal mouse inner ear. J Assoc Res Otolaryngol 2010 September 01;11(3):407-418.

(190) Kroll-Hermi A, Ebstein F, Stoetzel C, Geoffroy V, Schaefer E, Scheidecker S, et al. Proteasome subunit PSMC3 variants cause neurosensory syndrome combining deafness and cataract due to proteotoxic stress. EMBO Mol Med 2020 July 07;12(7):e11861.

(191) Okur MN, Mao B, Kimura R, Haraczy S, Fitzgerald T, Edwards-Hollingsworth K, et al. Short-term NAD(+) supplementation prevents hearing loss in mouse models of Cockayne syndrome. NPJ Aging Mech Dis 2020 January 07;6:1-z. eCollection 2020.

(192) Brown KD, Maqsood S, Huang JY, Pan Y, Harkcom W, Li W, et al. Activation of SIRT3 by the NAD(+) precursor nicotinamide riboside protects from noise-induced hearing loss. Cell Metab 2014 December 02;20(6):1059-1068.

(193) Ueyama T, Sakaguchi H, Nakamura T, Goto A, Morioka S, Shimizu A, et al. Maintenance of stereocilia and apical junctional complexes by Cdc42 in cochlear hair cells. J Cell Sci 2014 May 01;127(Pt 9):2040-2052.

(194) Ueyama T. Rho-Family Small GTPases: From Highly Polarized Sensory Neurons to Cancer Cells. Cells 2019 January 28;8(2):10.3390/cells8020092.

(195) Anttonen T, Kirjavainen A, Belevich I, Laos M, Richardson WD, Jokitalo E, et al. Cdc42-dependent structural development of auditory supporting cells is required for wound healing at adulthood. Sci Rep 2012;2:978.

(196) Zhu C, Cheng C, Wang Y, Muhammad W, Liu S, Zhu W, et al. Loss of ARHGEF6 Causes Hair Cell Stereocilia Deficits and Hearing Loss in Mice. Front Mol Neurosci 2018 October 02;11:362.

(197) Liu H, Pecka JL, Zhang Q, Soukup GA, Beisel KW, He DZ. Characterization of transcriptomes of cochlear inner and outer hair cells. J Neurosci 2014 August 13;34(33):11085-11095.

(198) Avenarius MR, Krey JF, Dumont RA, Morgan CP, Benson CB, Vijayakumar S, et al. Heterodimeric capping protein is required for stereocilia length and width regulation. J Cell Biol 2017 November 06;216(11):3861-3881.

(199) Richard C, Doherty JK, Fayad JN, Cordero A, Linthicum FH. Identification of target proteins involved in cochlear otosclerosis. Otol Neurotol 2015 June 01;36(5):923-931.

(200) Krey JF, Drummond M, Foster S, Porsov E, Vijayakumar S, Choi D, et al. Annexin A5 is the Most Abundant Membrane-Associated Protein in Stereocilia but is Dispensable for Hair-Bundle Development and Function. Sci Rep 2016 June 02;6:27221.

(201) Wang H, Lin C, Yao J, Shi H, Zhang C, Wei Q, et al. Deletion of OSBPL2 in auditory cells increases cholesterol biosynthesis and drives reactive oxygen species production by inhibiting AMPK activity. Cell Death Dis 2019 August 19;10(9):627-9.

(202) Li Y, Liu H, Barta CL, Judge PD, Zhao L, Zhang WJ, et al. Transcription Factors Expressed in Mouse Cochlear Inner and Outer Hair Cells. PLoS One 2016 March 14;11(3):e0151291.

(203) Basappa J, Turcan S, Vetter DE. Corticotropin-releasing factor-2 activation prevents gentamicin-induced oxidative stress in cells derived from the inner ear. J Neurosci Res 2010 October 01;88(13):2976-2990.

(204) Bas E, Goncalves S, Adams M, Dinh CT, Bas JM, Van De Water, T R, et al. Spiral ganglion cells and macrophages initiate neuro-inflammation and scarring following cochlear implantation. Front Cell Neurosci 2015 August 12;9:303.

(205) Murillo-Cuesta S, Rodriguez-de la Rosa L, Contreras J, Celaya AM, Camarero G, Rivera T, et al. Transforming growth factor beta1 inhibition protects from noise-induced hearing loss. Front Aging Neurosci 2015 March 20;7:32.

(206) Cho Y, Gong TW, Stover T, Lomax MI, Altschuler RA. Gene expression profiles of the rat cochlea, cochlear nucleus, and inferior colliculus. J Assoc Res Otolaryngol 2002 March 01;3(1):54-67.

(207) Lang H, Nishimoto E, Xing Y, Brown LN, Noble KV, Barth JL, et al. Contributions of Mouse and Human Hematopoietic Cells to Remodeling of the Adult Auditory Nerve After Neuron Loss. Mol Ther 2016 November 01;24(11):2000-2011.

(208) Goldbach-Mansky R, Kastner DL. Autoinflammation: the prominent role of IL-1 in monogenic autoinflammatory diseases and implications for common illnesses. J Allergy Clin Immunol 2009 December 01;124(6):1141-1.

(209) van Ingen G, Li J, Goedegebure A, Pandey R, Li YR, March ME, et al. Genome-wide association study for acute otitis media in children identifies FNDC1 as disease contributing gene. Nat Commun 2016 September 28;7:12792.

(210) Someya S, Yamasoba T, Kujoth GC, Pugh TD, Weindruch R, Tanokura M, et al. The role of mtDNA mutations in the pathogenesis of age-related hearing loss in mice carrying a mutator DNA polymerase gamma. Neurobiol Aging 2008 July 01;29(7):1080-1092.

(211) Zhu Y, Scheibinger M, Ellwanger DC, Krey JF, Choi D, Kelly RT, et al. Single-cell proteomics reveals changes in expression during hair-cell development. Elife 2019 November 04;8:10.7554/eLife.50777.

(212) Kalinec GM, Gao L, Cohn W, Whitelegge JP, Faull KF, Kalinec F. Extracellular Vesicles From Auditory Cells as Nanocarriers for Anti-inflammatory Drugs and Pro-resolving Mediators. Front Cell Neurosci 2019 November 29;13:530.

(213) Jamesdaniel SS. Noise induced changes in the expression of p38/MAPK signaling proteins in the sensory epithelium of the inner ear. Journal of Proteomics 2011;75(2):410; 410-424; 424.

(214) Cohen, Jennifer D J. D. Epithelial Shaping by Diverse Apical Extracellular Matrices Requires the Nidogen Domain Protein DEX-1 in . Genetics 2019;211(1):185; 185-200; 200.

(215) Yang CCH. Zona pellucida domain-containing protein β-tectorin is crucial for zebrafish proper inner ear development. PLoS ONE 2011;6(8):e23078; e23078.

(216) Zucca, Fabio A F. A. Interactions of iron, dopamine and neuromelanin pathways in brain aging and Parkinson's disease. Prog Neurobiol 2017;155:96; 96-119; 119.

(217) Murayama EE. Otolith matrix proteins OMP-1 and Otolin-1 are necessary for normal otolith growth and their correct anchoring onto the sensory maculae. Mech Dev 2005;122(6):791; 791-803; 803.

(218) Hermann AA. S100 calcium binding proteins and ion channels. Frontiers in Pharmacology 2012;3:67; 67.

(219) Kuo ME, Antonellis A. Ubiquitously Expressed Proteins and Restricted Phenotypes: Exploring Cell-Specific Sensitivities to Impaired tRNA Charging. Trends Genet 2020 February 01;36(2):105-117.

(220) Simonoska RR. Estrogen receptors in the inner ear during different stages of pregnancy and development in the rat. Acta Otolaryngol 2009;129(11):1175; 1175-1181; 1181.

stylefix

stylefix
